# Endosome restricted human TLR1 polymorphism highlights a role for MyD88 in Pam_3_CSK_4_ detection

**DOI:** 10.1371/journal.pone.0358252

**Published:** 2026-10-05

**Authors:** Christopher C. Wells, Jeffrey S. Bourgeois, Morgan A. Williams, Klemen Strle, Linden T. Hu, Tanja Petnicki-Ocwieja

**Affiliations:** 1 Graduate Program in Immunology, Tufts Graduate School of Biomedical Sciences, Boston, Massachusetts, United States of America; 2 Tufts Lyme Disease Initiative, Tufts University School of Medicine, Boston, Massachusetts, United States of America; 3 Department of Biology and Biotechnology, Worcester Polytechnic Institute, Worcester, Massachusetts, United States of America; 4 Department of Molecular Biology and Microbiology, Tufts University School of Medicine, Boston, Massachusetts, United States of America; Universidad Miguel Hernández de Elche, SPAIN

## Abstract

Toll-like receptor 2/1 (TLR2/1) heterodimers detect triacylated lipoproteins. Although TLR2/1 complexes localize to both the plasma membrane and endosome, the functional contributions of compartment-specific signaling are not completely defined. A common human TLR1 polymorphism (rs5743618), at which one allele substitutes isoleucine for serine adjacent to the transmembrane domain, restricts TLR1 intracellularly and is associated with an increased risk of antibiotic-refractory Lyme arthritis. This mis-localization provides a model to examine how receptor positioning influences downstream signaling. Here, we investigated the rs5743618 polymorphism’s effects on TLR1 localization and compartment-specific NF-κB activation. Using a CD14+ TLR2+ HEK cell reporter system that enables transient expression of both allelic variants, we confirmed that the polymorphism is excluded from the cell surface and retained intracellularly, whereas the ancestral allele localizes to both compartments. Functionally, the ancestral allele signaled from both compartments, while the derived allele signaled exclusively from endosomes. Spatially restricted signaling exhibited influence on IkBα degradation and NF-κB activation compared with the ancestral allele. This activation was dependent on MyD88 localized at ligand-containing endosomes, establishing a role for this adapter in intracellular TLR2/1 signaling. These findings provide insight into how this common polymorphism impacts inflammatory outcomes.

## Introduction

Innate immune detection of pathogen associated molecular patterns (PAMPs) by Toll-like receptors (TLRs) is an important first step in host defense against infectious microorganisms [1–3]. TLRs are expressed both at the plasma membrane and within subcellular compartments, enabling surveillance of pathogens in distinct cellular locations. They are widely expressed in cells of the immune system but also in other cell types, including epithelial cells at barrier tissues such as the skin, ensuring pathogen detection outside of classical lymphoid and myeloid populations [4,5].

Among the TLRs expressed by mammals is TLR1, which is crucial in the initial detection and subsequent triggering of an inflammatory cascade in response to bacterial infection. TLR1 pairs with TLR2 to form a TLR2/1 heterodimer which detects triacylated lipopeptides expressed by both pathogenic and commensal bacteria. Previous studies have shown that TLR2/1 complexes localize both to the cell surface and intracellular endosomal compartments, and initiate signaling cascades that activate pro-inflammatory and type I IFN signaling [2,6–11]. Although considerable work has examined the trafficking of ligand-containing vesicles and associated signaling molecules, it has been difficult to strictly isolate endosomal TLR2/1 signaling from that of the plasma membrane [2,12]. As a result, the precise composition and functional distinction of TLR2/1 signaling complexes at these two locations remained incompletely defined. Studies have shown that adaptors such as TRAM and TRIF contribute to intracellular TLR2/1 signaling, particularly in promoting type I IFN activation. MyD88 has also been observed to localize to ligand containing endosomes [9,13], however, it remains unclear if it participates in transducing NF-κB signals from the endosome or whether this is mostly controlled by the cell surface activated signaling cascade [9,10].

Triacylated lipopeptides are a broad category of PAMPs and are expressed by many pathogens that critically affect the human immune response through their interaction with TLR2/1. Mycobacterial infection with pathogens such as *Mycobacterium tuberculosis* and *Mycobacterium leprae* interface with TLR2/1 heterodimers during transmission as well as intracellular infection of alveolar and cutaneous macrophages [14,15]. This interaction with TLR2/1 and other TLRs is crucial to the characteristics of the immune response that forms against *M. tuberculosis* and *M. leprae*, which meaningfully influences the progress of disease [16–18]. In the case of infection with *Borrelia burgdorferi* (*Bb*), a Lyme disease-causing spirochete, human immune cells as well as somatic cells within the dermis encounters *Bb* PAMPs following a tick bite. As *Bb* replicates and disseminates through its host, the scope of TLR mediated immune detection expands to other organs and tissues including the synovium of major joints of the lower extremities, resulting in Lyme arthritis [19–21]. It is well established that lipopeptide components of *Bb’s* bacterial membrane can be detected by TLR2/1 complexes localized both to the cell surface and intracellular endosomal compartments [1,22,23], where they sample ligands and initiate signaling cascades that activate pro-inflammatory and type I IFN signaling.

A common single nucleotide polymorphism in *TLR1* (rs5743618, Major Alleles = T or A, Minor Allele = G or C), alters an amino acid residue adjacent to TLR1’s transmembrane domain (Isoleucine to Serine), resulting in a nearly complete restriction of TLR1 to intracellular compartments [17,24,25]. This variant has a high minor allele frequency in individuals of European descent (European Allele Frequency = 0.508) [17,18,25]. This polymorphism is associated with an increased risk of antibiotic refractory Lyme arthritis following antibiotic treatment of *Bb* infection [26]. Interestingly, *in vitro* stimulations of the refractory inflammation allelic variant with synthetic ligands as well as whole mycobacterium and mycobacterial membranes do not confer enhanced TLR1 signaling and instead exhibit diminished inflammatory responses [17,18,25] while stimulations with other bacteria like *Bb* produces mixed responses in different investigations [27–29]. In contrast, the ancestral TLR1 allele, which allows TLR1 localization to both the plasma membrane and intracellular compartments, produces stronger inflammatory responses *in vitro* and is associated with a lower incidence of refractory Lyme arthritis [26]. These findings suggest that altered receptor localization may influence the quantity, quality, and timing of immune signaling. We hypothesize that compartment-specific differences in TLR1 signaling affect antibacterial priming and the subsequent engagement of regulatory pathways required for resolution of inflammation.

In this study we sought to define how the rs5743618 polymorphism alters TLR1 localization and compartment-specific signaling. We modified an existing HEK cell reporter system where we enabled transient overexpression of both allelic variants to assess receptor localization and NF-κB activation. Consistent with prior reports, we confirm that the TLR1 polymorphism is restricted to intracellular compartments and does not localize to the cell surface. We further demonstrate that ligand trafficking is sufficient to enable TLR1 activity in both allelic variants and that the ancestral receptor signals from both the cell membrane and intracellular spaces. In contrast, cells expressing the derived allelic variant signal from intracellularly restricted locations. Using size-exclusion beads to restrict ligand access, we identify spatial differences in NF-κB activation between the two variants that are influenced by cellular compartmentalization. Finally, using siRNA knockdowns, we show that the MyD88 adaptor is required for NF-κB activation from ligand-containing endosomes, supporting its functional role in intracellular TLR2/1 signaling.

## Results

### Cells expressing TLR1 602S retain functional signaling capacity

To investigate the mechanistic basis underlying the association between rs5743618 and altered inflammatory responses, we adapted a previously described HEK293 reporter system (Invivogen) by transiently expressing either the plasma membrane and intracellularly localized TLR1 (602I) or the exclusively intracellular TLR1 (602S) variants (Fig 1A and 1B). HEK Blue NF-κB reporter cells, deficient in TLR1 and TLR6 (HEK^SEAP+/CD14+/TLR2+/TLR1-/TLR6-^) were used as a parental cell line to express both TLR1 variants. These cells link NF-κB activity to secreted embryonic alkaline phosphatase transcription, detectable by cleavage and color conversion of a soluble substrate in media. Transfected HEK Blue 1/6KO cells expressed equivalent levels of TLR1 protein regardless of the transfected TLR1 variant (Fig 1C). Cells were stimulated for 18 hours with the TLR2/1 specific ligand Pam_3_CSK_4_ and the percent change in NF-κB activity was quantified. The data showed that both variants induced NF-κB activation, but the TLR1 602S variant had significantly reduced activity (Fig 1D). This was consistent with previous findings and validated our HEK cell system [17,18].

**Fig 1:**
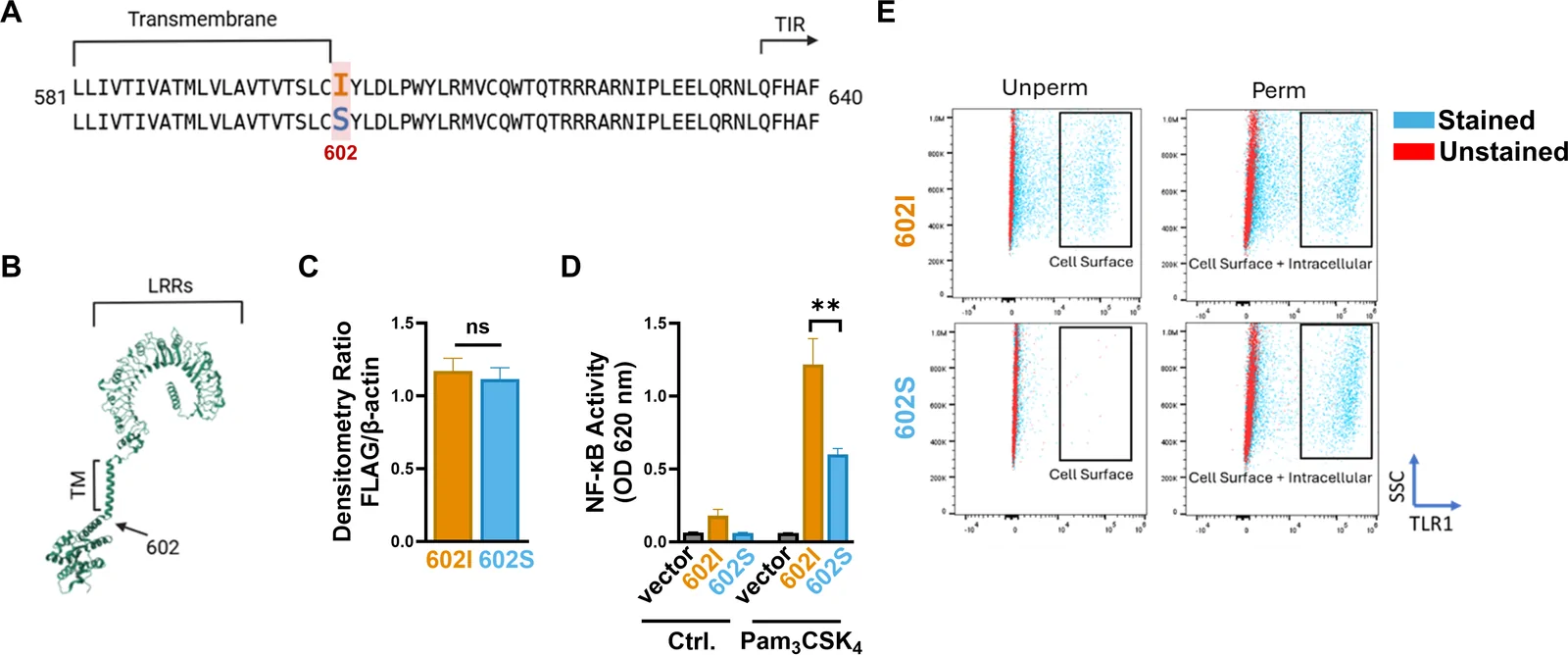
Overexpression constructs for TLR1 variants recapitulate previously defined phenotypes. (A) Translation of the transmembrane domain, loci of the polymorphism, and the beginning of TIR signaling domain. (B) Crystal structure of TLR1 with leucine rich repeat (LRR), transmembrane (TM), and polymorphic residue (602) labeled (structure from AlphaFold [30]). (C) WB densitometry ratio of FLAG and β-actin n = 3. (D) HEK Blue SEAP in HEK Blue TLR1/6KO cells transfected with 602I and 602S, stimulated with 100 ng/mL Pam_3_CSK_4_ for 18 hours n = 3 (C, D two-way ANOVA). (E) Flow cytometry of TLR1 staining. HEK Blue TLR1/6KO cells transfected with TLR1 allelic variants. Stained in unpermeabilized and permeabilized conditions.

The localization of TLR1 in the constructs was assessed by flow cytometry using fluorescent anti-TLR1 labeling of unpermeabilized and permeabilized cells. Cells transfected with TLR1 602I showed TLR1 localization in both permeabilized and unpermeabilized conditions, while cells transfected with TLR1 602S only stained positively for TLR1 when permeabilized (Fig 1E). This was consistent with previous observations of TLR1 602S receptor localization [17,18].

### The TLR1 602S variant signals from endosomal compartments

The precise intracellular localization of TLR1 602S has not been fully defined. To determine whether the receptor can exit the endoplasmic reticulum (ER) and reach endosomes containing internalized ligand, we developed a size-restricted ligand delivery system. Pam_3_CSK_4_-biotin was conjugated to fluorescent streptavidin beads that were either small enough to be internalized by non-professional phagocytic HEK cells (0.5 µm diameter) or too large for the HEK cells to capably internalize (5 µm diameter) (Fig 2A and Fig 2B). Cells expressing TLR1 602I responded to both bead sizes. In contrast, cells expressing TLR1 602S failed to respond to 5 µm beads but responded to 0.5 µm beads (Fig 2C). These findings indicate that TLR1 602S signals only from intracellular compartments. This conclusion was further supported by using streptavidin-coated plates presenting immobilized Pam_3_CSK_4_, which cannot be removed from the plate surface and internalized (Fig 2D). Under these conditions, TLR1 602S expressing cells failed to activate NF-κB, whereas TLR1 602I expressing cells responded normally (Fig 2E). These data suggest that HEK cells expressing TLR1 602S can access ligand containing endosomes and activate NF-κB, but are unable to trigger NF-κB activity to extracellularly restricted ligands.

**Fig 2:**
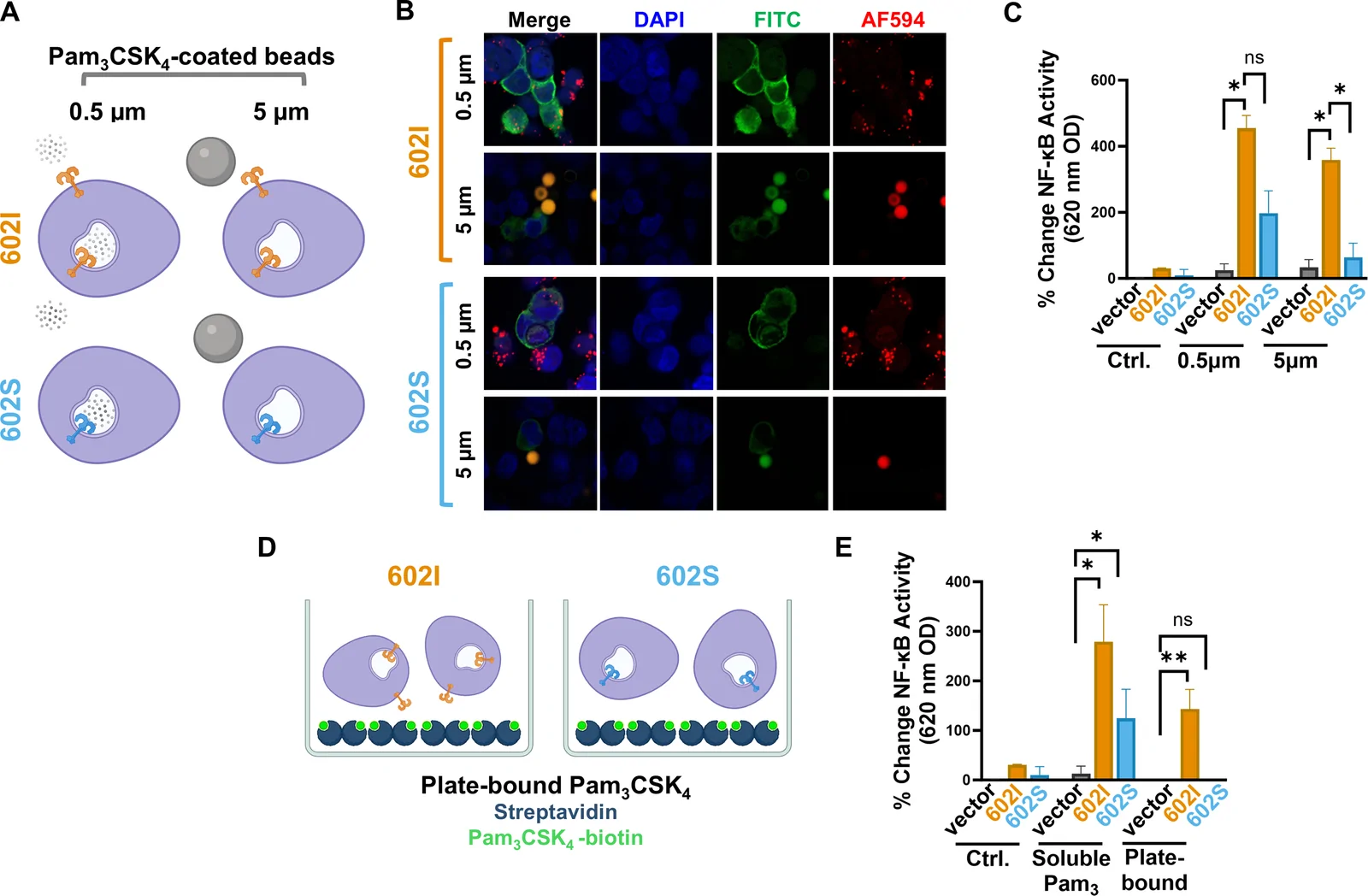
TLR1 variant constructs are activated in a spatially dependent manner. (A) Schematic for Pam_3_CSK_4_-bead and TLR1 variant receptor localization (B) Confocal microscopy of TLR1 602I and 602S expressing HEK Blue cells co-incubated with Pam_3_CSK_4_-biotin conjugated 0.5 µm (red) or 5 µm (yellow) beads for 1 hour. Cell nuclei stained with DAPI, anti-FLAG stained with anti-mouse AF488 (C) HEK Blue SEAP Assay in HEK Blue TLR1/6KO transfected with TLR1 variants. Stimulated 1 µg/mL Pam_3_CSK_4_-biotin conjugated to 0.5 µm streptavidin beads, 1 µg/mL Pam_3_CSK_4_-biotin conjugated to 5 µm streptavidin beads n = 3. (D) Schematic for plate-bound Pam_3_CSK_4_ and TLR1 variant localization (E) HEK Blue SEAP Assay in HEK Blue TLR1/6KO transfected with TLR1 variants. Stimulated with 100 ng/mL soluble Pam_3_CSK_4_-biotin and streptavidin coated plates conjugated with 10 µg/mL Pam_3_CSK_4_-biotin n = 3 (C two-way ANOVA, E one sample t-test).

### TLR1 602I and 602S signaling is spatially and temporally distinct

To determine how TLR1 localization and compartmentalization effects signaling kinetics, we measured the nuclear translocation of the NF-κB transcription factor over a 3-hour stimulation with soluble Pam_3_CSK_4_.

Stimulation of cells expressing TLR1 602I with soluble Pam_3_CSK_4_ induced a rapid degradation of NF-κB’s negative regulator, IkBα, at 30 minutes and subsequent large increase of IkBα band density at 120 minutes, approximately back to basal levels (Fig 3A and 3C). We observed biphasic activity of NF-κB p65 translocation when stimulating TLR1 602I cells, with two peaks occurring at 60 and 120 minutes (60 min, 120 min; Fig 3C and 3D) In contrast, the cells transfected with TLR1 602S showed steady IkBα reduction without a rebound (Fig. 3A and 3C). In addition, NF-κB p65 translocation observed in TLR1 602S expressing cells gradually increased over the duration of the entire time course (Fig 3D and 3E).

**Fig 3:**
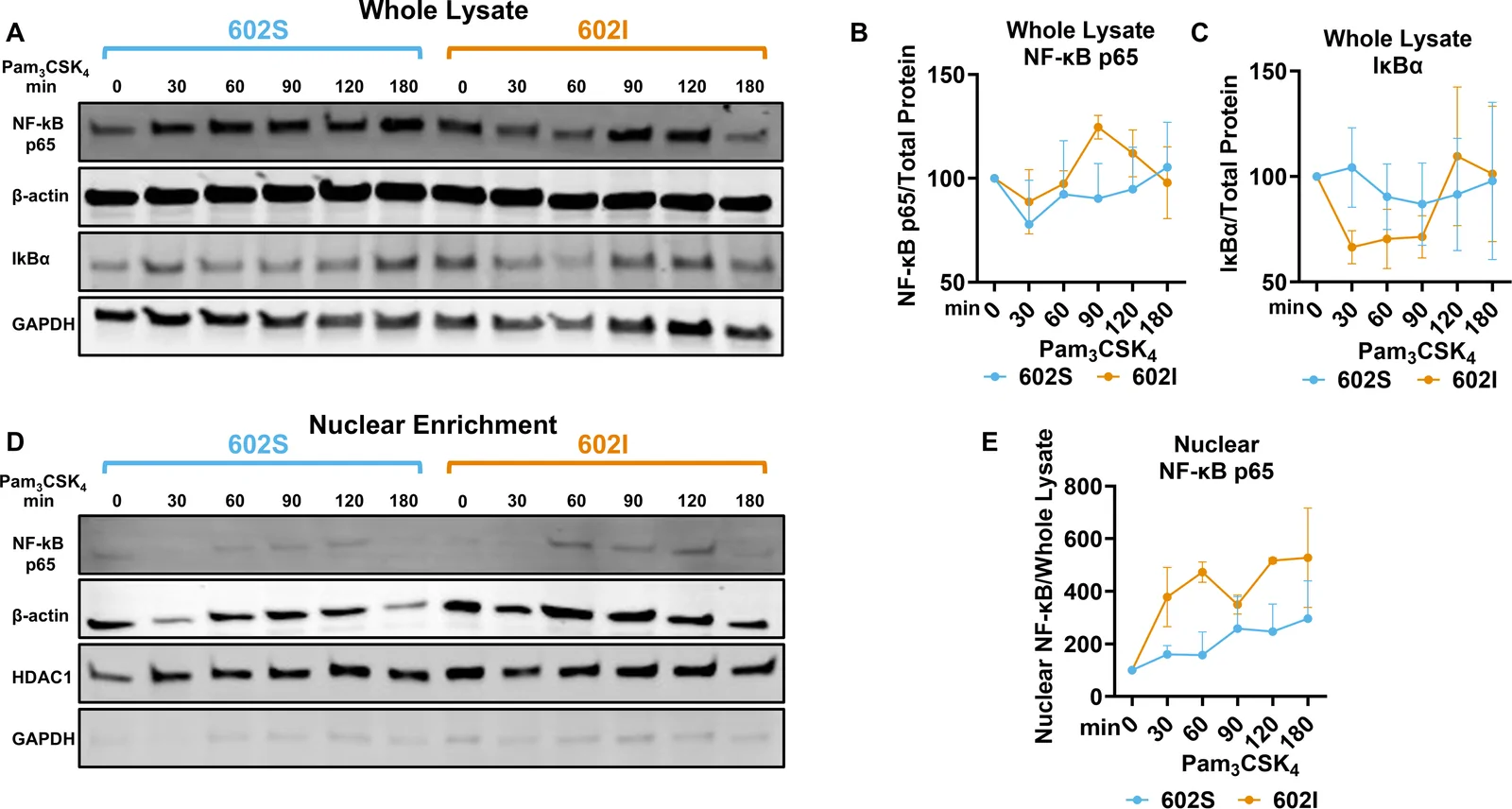
Soluble Pam3CSK4 signaling results in regulated nuclear export of NF-κB p65 in 602I. (A) Whole cell lysate of cells transfected with 602I and 602S TLR1 variants stimulated with 100 ng/mL soluble Pam3CSK4-biotin for 0, 30, 60, 90, 120, 180 minutes (B) Band density quantification of NF-κB p65 normalized to total protein measured by UV activated total-protein staining (C) Band density quantification of IkBα normalized to total protein measured by UV activated total-protein staining n = 3. (D) Nuclear enrichment of cells transfected with 602I and 602S TLR1 variants stimulated with 100 ng/mL soluble Pam3CSK4-biotin for 0, 30, 60, 90, 120, 180 minutes (E) Band density quantification of NF-κB p65 normalized to total nuclear protein measured by UV activated total-protein staining and to protein quantification from whole lysate n = 3. Full images available S1 Raw Images.

When stimulation was restricted to the plasma membrane using Pam_3_CSK_4_-biotin conjugated 5 µm streptavidin beads, which are unable to be endocytosed by HEK cells, cells with TLR1 602I displayed a similar degradation of IkBα at 30 minutes compared to soluble Pam_3_CSK_4_, but a delayed recovery of IkBα band density (Fig 4A and 4C), as well as steady increase in NF-κB translocation without the biphasic pattern seen with soluble Pam_3_CSK_4_ (Fig 4D and 4E). The cells with TLR1 602S showed minimal NF-κB p65 translocation under these conditions (Fig 4D and 4E), and IkBα dynamics mirrored these findings (Fig 4A and 4C).

**Fig 4:**
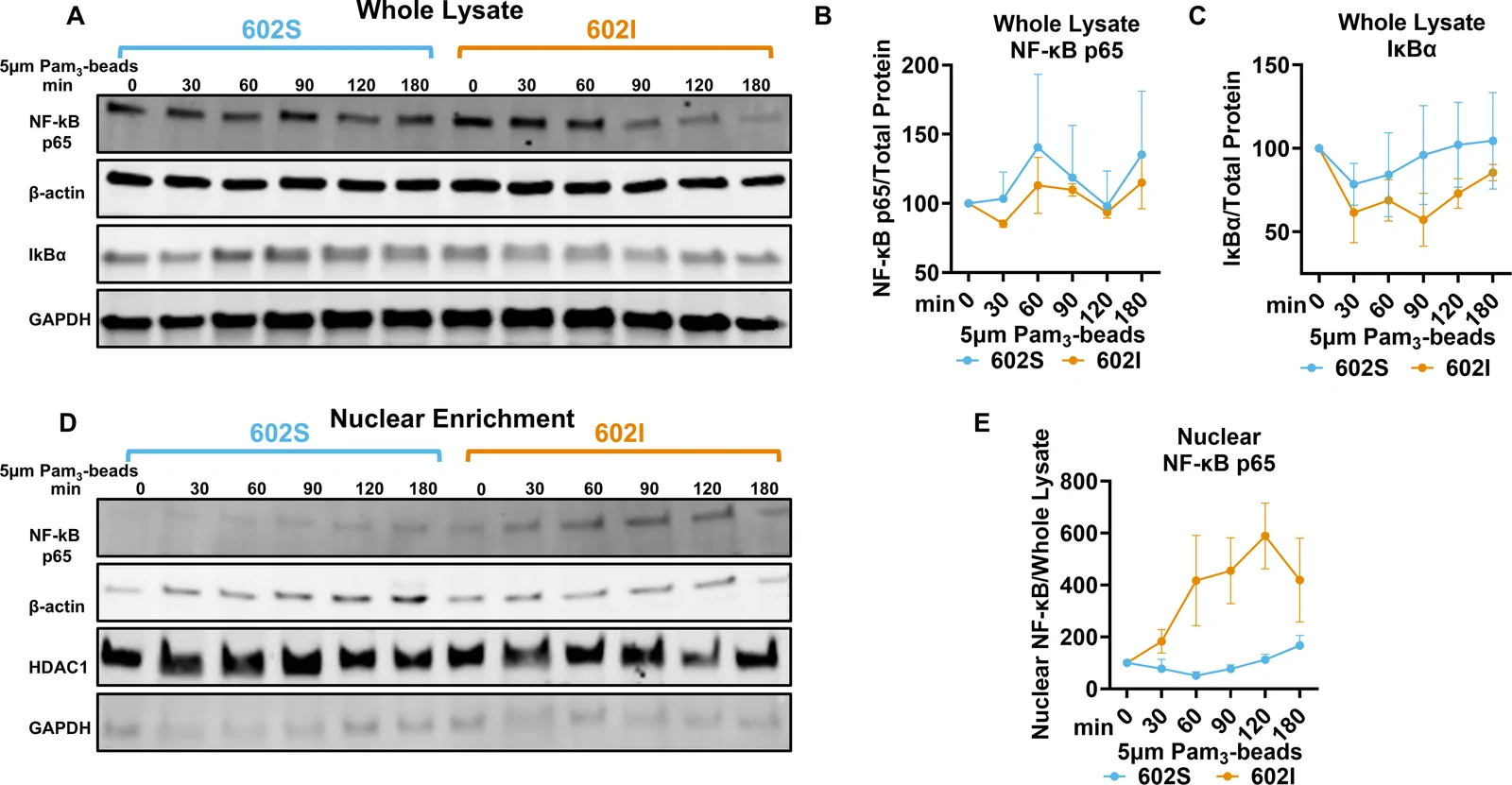
5 µm Pam3CSK4 signaling results in delayed nuclear export of NF-κB p65, prolonged degradation of IkBα in 602I. (A) Whole cell lysate of cells transfected with 602I and 602S TLR1 variants stimulated with 1 µg/mL Pam3CSK4-biotin conjugated to 5 µm streptavidin beads for 0, 30, 60, 90, 120, 180 minutes (B) Band density quantification of NF-κB p65 normalized to total protein measured by UV activated total-protein staining (C) Band density quantification of IkBα normalized to total protein measured by UV activated total-protein staining n = 3. (D) Nuclear enrichment of cells transfected with 602I and 602S TLR1 variants stimulated with 1 µg/mL Pam3CSK4-biotin conjugated to 5 µm streptavidin beads for 0, 30, 60, 90, 120, 180 minutes (E) Band density quantification of NF-κB p65 normalized to total nuclear protein measured by UV activated total-protein staining and to protein quantification from whole lysate n = 3 Full images available S1 Raw Images.

### The TLR1 602S endosomal variant utilizes MyD88 for NF-κB activation

MyD88 and its co-adaptor TIRAP/MAL are well established mediators of NF-κB activation downstream of plasma membrane TLR signaling as well as endosomal TLRs 7, 8 and 9. For receptors that signal from both plasma membrane and endosomal compartments, including TLR2, signaling has been attributed in large part to TRAM-TRIF dependent pathways for induction of IRF transcription factors and type I IFNs from the endosome [2,12,31,32]. The contribution of MyD88 to endosomal TLR2/1 signaling has not been clearly defined.

To expand our understanding of signaling diversity from TLRs located within subcellular compartments, we co-transfected each TLR1 variant with MyD88-targeting siRNA and efficient knockdown confirmed by RT-qPCR (Fig 5A). MyD88 depletion reduced NF-κB activation by approximately 50% in cells expressing 602I, while NF-κB activation in 602S expressing cells was below detection levels. (Fig 5B). These findings indicate that MyD88, alongside TRAM/TRIF, is among the signaling adaptor proteins that contribute to NF-κB activation mediated by TLR1 signaling in endosomal compartments. Because the role of TRAM-TRIF in endosomal TLR4 and TLR2 signaling leading to type I IFN activation, we wanted to assess the role of TLR1 variants in type I IFN activation. Therefore, we assessed IFNB1 and NF-κB induction comparing Pam_3_CSK_4_ and poly I:C stimulation, the latter a ligand known to induce type I IFNs through a TLR3-TRIF complex. While poly I:C robustly induced NF-κB dependent SEAP activity independently of TLR1 expression, minimal *IFNB1* expression was detected under either condition (S1 Fig). This suggested that while TRIF is functional in these cells, the type I IFN induction machinery did not exhibit transcriptional capacity, and we could not assess the role of the TLR1 variants in type I IFN induction. In addition, expression analysis indicated non-detectable levels of *TRAM*, a very low levels of *TRIF* expression, rendering the HEK cell system unsuitable for depletion of these adaptors (S2 Fig). Together, these results demonstrate that MyD88 plays a functional role in endosomal TLR2/1 activity and contributes to NF-κB activation.

**Fig 5:**
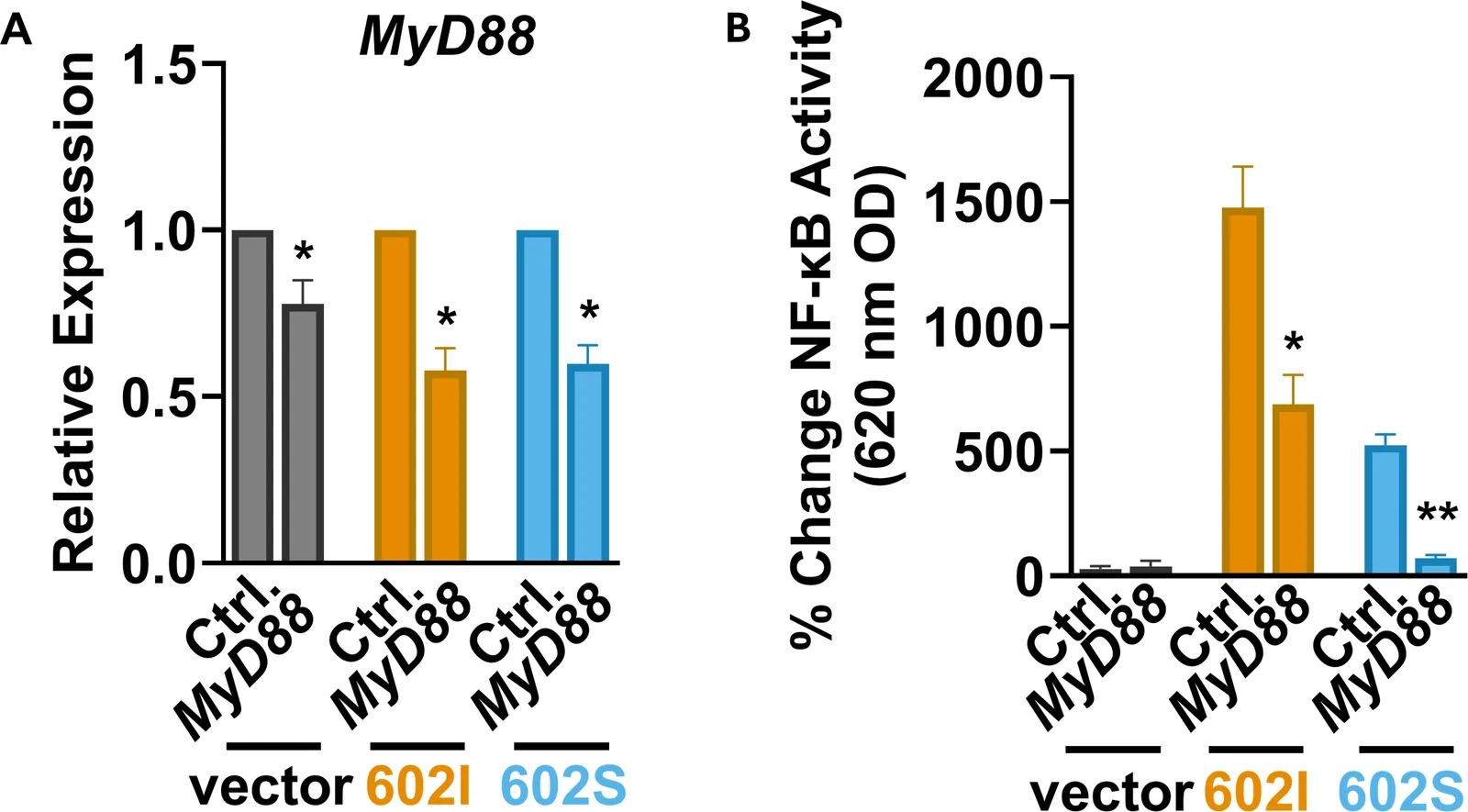
NF-κB activity is significantly reduced in 602S when co-transfected with MYD88 siRNA pool. (A) RT-qPCR relative expression of MYD88 after plasmid/siRNA co-transfection n = 6. (B) HEK Blue SEAP activity in HEK Blue TLR1/6KO transfected with TLR1 variants and siRNA. Stimulated with 100 ng/mL Pam_3_CSK_4_ for 18 hours n = 6 (A, B one-sample t-test).

## Discussion

In this study we used allele-specific overexpression constructs to investigate how the *TLR1* rs5743618 polymorphism alters signaling as a consequence of differential trafficking. We demonstrated that in a HEK Blue reporter cell system TLR1 602S signaling is affected by size-dependent ligand access.

We observed that bead-bound ligands, too large for internalization, were unable to activate TLR1 602S while the signaling capability of TLR1 602I was unaffected by size limitation and responded to all Pam_3_CSK_4_ stimulations regardless of bead diameter or plate-conjugation. Together with anti-TLR1 staining data, these signaling results suggest that the TLR1 602S variant is indeed endosomally located and unable to gain access to extracellular ligands.

By using size exclusion of ligands, we separated the activity of plasma membrane TLR1 from endosomal signaling pathways to interpret how NF-κB transduction is induced from either TLR1 location. In our whole lysate and nuclear enrichments, we observed that stimulating either TLR1 allelic variant triggered degradation of IkBα and nuclear translocation of NF-κB p65, however we only measured a strong reinduction of IkBα when stimulating both plasma membrane and endosomal TLR1 602I. Activation of solely endosomal TLR1 602S or solely plasma membrane TLR1 602I resulted in only gradual reinduction of IkBα. Novel translation and utilization of IkBα to export NF-κB p65 from the nucleus is a well understood principle of the negative feedback loop of the NF-κB transcriptional pathway [33]. TLRs are heavily involved in many aspects of infection and immunity, and faults in their activation and signaling can be both protective and harmful during infections and autoimmune disease [34–37]. Some of these aberrant pathologic outcomes are triggered by loss-of-function SNPs that damage signal induction by TLRs, producing an error in negative feedback pathways [34,35]. The results from this investigation of location-specific TLR1 activation could indicate that induction of crucial negative regulators in the NF-κB transcriptional pathway, such as IkBα, are not as robustly induced via TLR1 if signaling is not instigated from both the plasma membrane and endosome. While *TLR1* rs5743618 is indicated both as a protective and harmful SNP depending on the implicated disease or autoimmune syndrome [16,17,24,29,38], one possible hypothesis is that it may be contributing to these outcomes through dysregulation of negative feedback of NF-κB as we observed in our whole lysate and nuclear enrichment.

The HEK model in this research is not without limitations, as its function as a cell-signaling platform is somewhat restricted. While the experimental overexpression of our TLR1 construct as well as the proprietary expression of TLR2 and CD14 enables insights into specifically TLR2/1 signaling dynamics as it is carried out by MyD88, leading to eventual NF-κB activity, it is insufficient for investigating the impact of other PRRs, signaling adaptors in these pathways, and the activity of IRF transcription factors. An illustration of this limitation is the deficiency in type I IFN induction and *TRAM/TRIF* expression. While there is some signaling activity downstream of TRIF dependent TLR3 activation with PolyI:C (S1 Fig), there is no induction of *IFNB1* expression (S1 Fig). This may be due to insufficient expression of TRAM/TRIF, as relative expression of these two adaptors in the transfected HEK Blue cells was at or below the limit of quantification by RT-qPCR (S2 Fig). This limitation affected our ability to draw conclusions about the cumulative transcriptional activity of the TLR1 variants or the immunological outcomes of TLR1 602S activity compared to TLR1 602I. Therefore, developing a comprehensive understanding of this signaling pathway as it relates to disease and the genetic determinants of symptoms from a bacterial infection requires a more sophisticated platform than HEK cells. This also highlights a limitation of the use of synthetic Pam_3_CSK_4_ TLR2/1 activation. While it recapitulates specifically the triacylated lipopeptide components of bacterial membranes, whole bacteria have a much more diverse set of PAMPs that may be detected by a broad array of receptors expressed in immune and somatic cells. When taking the results of this work into account, we can conclude that there are influences of the TLR1 602S variant on transcription and negative regulation, but surely there are ways in which this feedback influences other signaling pathways separate from TLR2/1 that are insufficiently addressed with synthetic ligands of a single immune receptor pathway.

There is a wealth of investigations surrounding adaptor molecules utilized by TLR dimers for signal transduction, with a traditional paradigm for TLR2/1 that proposes that extracellular membrane structure allows for recruitment and use of MAL/MyD88 for signal induction, while the endosomal lipid structure is more traditionally associated with TRAM/TRIF mediated signaling [39–41]. Previous findings have suggested that there may be a role for MyD88 at endosomes as well [9,42–44], and have demonstrated varying degrees of permissiveness for plasma membrane located MAL with endosomal lipid membranes [45]. By utilizing our endosome restricted TLR1 602S and co-transfection of a *MYD88* siRNA pool we found that knock-down of *MYD88* expression not only reduces NF-κB activity of TLR1 602I, but also endosomal TLR1 602S. While this does not exclude a role for TRIF or TRAM/TRIF in TLR2/1 endosomal signaling, our data corroborates a growing body of evidence that MyD88 does function as a signaling adaptor for TLR2/1 at the endosome. Based on our previous findings, we propose a model of TLR2/1 endosomal signaling that includes MyD88 alongside TRAM and TRIF in cells such as macrophage.

An inquiry into *TLR1* polymorphism rs5743618 specifically in the context of *Bb* stimulation was carried out in primary human PBMCs, demonstrating that inflammatory gene induction in primary monocytes expressing TLR1 602S is amplified upon repeated or prolonged stimulation by *Bb* or Pam_3_CSK_4_. Alternatively, cells expressing TLR1 602I successfully tolerate repeated stimulations by downregulating release of cytokines and interferons such as IL6, IL1, and IFNα upon repeated stimulation [29]. The tolerant resolving phenotype exhibited by TLR1 602I PBMCs is a well reported a normal component of innate immune memory wherein innate immune cells exposed to *Bb* respond strongly upon initial contact with the bacterium, but negative regulation of inflammatory gene expression counteracts the inflammatory response, producing an innate immune tolerant phenotype to prolonged or repeated interactions with the bacterium [46–48]. It may be possible that the continued exacerbation of inflammatory genes observed in TLR1 602S human PBMCs, the utility of negative regulation inducing innate immune tolerance, and the reduced utilization of negative regulators by TLR1 602S from this research are each components of a TLR2/ TLR1 602S mediated failure to induce tolerance to *Bb.*

## Materials and methods

### Cells, constructs, and siRNA

HEK Blue TLR1/6 KO Cells (Invivogen hkb-htlr2k16)) or HEK Blue TLR2 (Invivogen hkb-htlr2) were cultured in DMEM (Corning MT10013CV) supplemented with 10% FBS (SeraPrime F31016-500). HEK Blue Selection (Invivogen hb-sel) was added to tissue culture media following the manufacturer's instructed dilution of 1:250.

pCMV-3Tag-3a (SnapGene) was utilized as the vector backbone for cloning of TLR1 allele constructs (Supp.1). siRNA pool for MyD88 (Dharmacon M-004769-01-0010) was used for knockdown during co-transfection and non-targeting siRNA (Dharmacon D-001210-05-05) was used as a control. siRNA and control were diluted in 5x siRNA buffer (Dharmacon B-0020000-UB-100) and dilution to a working concentration was measured on a nanodrop.

### Transfection

HEK Blue 1/6KO cells were plated the night before transfection at the recommended cell density for the culture vessel volume. 1.0 x 10^6^ cells per well in a 6-well dish and 1.8 x 10^5^ cells per well in a 24-well dish or chamber of a chamber slide. The day of transfection cell culture media was removed and replaced with the recommended volume of fresh 10% FBS supplemented culture media to carry out transfection at least 30 minutes prior to coincubation of lipid-DNA complexes. In a 6-well plate this is 2 mL, and in a 24-well plate this is 500 µL of media.

Lipofectamine 3000 (Thermo Fisher L30000075) was used for plasmid transfection by following the manufacturer's guidelines in the packaged protocol according to cell culture vessel volume. Dilutions of plasmid and reagent were made in Opti-MEM media (Gibco 31985−062). The following volumes and concentrations were used for a single well of a 6-well plate. In one tube 1.25 µg of vector, 1.25 µg of experimental expression vector, and 2.5 µL of P300 in 50 µL of Opti-MEM. In a second tube 2.5 µL of lipofectamine 3000 in 50 µL of Opti-MEM. The two tubes were combined and incubated at room temperature for 15 minutes. A total of 100 µL of the mixed volume was added dropwise to a well and incubated at 37°C for 24 hours. The transfection culture media was replaced with fresh supplemented media and the cells were rested at 37°C for 24 hours. For 24-well format, media was replaced with 500 µL of fresh media. The following volumes and concentrations were used for a single well of a 24-well plate or chamber of a chamber slide. In one tube 250 ng of vector, 250 ng of experimental expression vector, and 1 µL of P3000 was diluted in 25 µL of Opti-MEM. In a second tube 1.5 µL of lipofectamine 3000 was diluted in 25 µL of Opti-MEM. The two tubes were combined and incubated at room temperature for 15 minutes. 50 µL of the mixed volume was added dropwise to a well and incubated at 37°C for 24 hours. The transfection culture media was replaced with fresh supplemented media, and the cells were rested at 37°C for 24 hours.

Co-transfection of plasmid and siRNA was performed with Lipofectamine 2000 (Thermo Fisher 11668019) in 24-well plates at 1.8 x 10^5^ cells per well. The following volumes and concentrations were used for a single well of a 24-well plate. 200 ng of plasmid DNA and 6 pmol of siRNA pool were diluted in 50 µL Opti-MEM media in multiple tube. In another tube, 0.75 µL of lipofectamine 2000 was diluted in 50 µL of Opti-MEM media and incubated for 5 minutes at room temperature. 50 µL of diluted lipofectamine 2000 was then combined with each 50 µL tube of diluted DNA/siRNA and incubated for 15 minutes at room temperature. The lipofectamine-DNA/siRNA mixture was then added dropwise to cells in 500 µL of growth media. Cells were incubated at 37°C for 48 hours prior to use.

### Antibodies, reagents, and equipment

The TLR2 ligand, Pam3CSK4 (Invivogen tlrl-pms) and TLR3 ligand Poly I:C (Invivogen tlrl-picb) were used for stimulations. 5 µm streptavidin green fluorescent beads (Nanocs Si5u-FCSV-1), 0.5 µm streptavidin red fluorescent beads (Nanocs Si500-RBSV-1), and biotin Pam3CSK4 (Invivogen tlrl-bpms) were used for size exclusion stimulations. Biotin-Pam3CSK4 was conjugated to streptavidin in a 1 µg/mL solution for 1 hour at room temperature while mixing.

Anti-TLR1-PE (eBioscienc GD2.F4), fixable live/dead aqua (Invitrogen L34965), and intracellular staining kit (Invitrogen 00-5523-00) were used for flow cytometry performed on a BD Biosciences Attune. Subcellular fractionation kit (Thermo Fisher 78840), anti-FLAG (Sigma Aldrich F1804-50UG), NF-κBp65 (CST 8242S), IkBα (Biolegend 662402), GAPDH (CST 2118T), HDAC1 (CST 5356T), and β-actin (CST 3700S) were utilized for western blotting of cytoplasmic and nuclear proteins and quantification of FLAG expression.

Prolong GOLD anti-fade DAPI mount (Thermo Fisher P36935) and LAB-TEK II (Chamber Slides (Thermo Fisher 154941PK) were used for confocal microscopy on a Nikon A1R confocal microscope using a 63X oil objective with NIS elements software. Images were processed and quantified using ImageJ

### HEK Blue assay

Transfected HEK Blue cells were plated into a black-walled, clear-bottomed 96 well plate at 2.8 x 10^5^ cells per well in 180 µL of HEK Blue Detection Media (Invivogen hb-det2). For cellular stimulation 20 µL of unmodified detection media was added as a control, 20 µL of 1 µg/mL soluble Pam3CSK4 or 20uL of 0.25x 5 µm streptavidin beads conjugated with biotin-Pam3CSK4 was added for stimulating NF-κB SEAP reporter activity. Optical density (OD) at 620 nm was measured every 30 minutes for 18 hours using a BioTek II spectrophotometer at 37°C

### Western Blotting

Cells were lysed in RIPA cell lysis buffer (Santa Cruz sc-24948) or in cellular fractionation buffer. Lysate was mixed with Laemmli SDS buffer (Biorad 1610737) supplemented with 5% β-mercaptoethanol (Gibco M3148-25ML) and boiled at 95°C for 5 minutes, then cooled on ice. 13 µL of cooled sample was loaded into 15-well 4–12% PROTEAN BisTris gels (Biorad 4568086). The gel was run on a Biorad PowerPac HC at 90 volts for 40 minutes. The total protein stain was activated on a Biorad ChemiDoc XRS+ for 1 minute. The gel was transferred to methanol activated PVDF in a Biorad Turboblot with Turboblot 5x transfer buffer (Biorad 10026938).

The membrane was blocked in PBS (Thermo Fisher 10-010-049) + 5% BSA (Rockland BSA-50) blocking buffer for 30 minutes at room temperature. Primary antibody diluted 1:1000 in blocking buffer was incubated on the membrane at 4°C with oscillation overnight. The membrane was washed 3 times in PBS + 0.05% tween 20 (Sigma P1379-500mL) for 5 minutes while oscillating. Secondary antibodies for anti-mouse (Licor 926-68070) and anti-rabbit (Licor 926-32211) were diluted 1:10,000 in blocking buffer and incubated on the membrane in the dark at room temperature for 30 minutes. The membrane was washed 3 times in PBS + 0.05% tween 20 for 5 minutes while oscillating. The membrane was imaged on a Licor Odyssey, and densitometry of bands was performed using ImageJ.

### Flow Cytometry

3.0 x 10^5^ cells were resuspended in 300 µL FACS buffer (PBS + 2% FBS + 2 mM EDTA (Thermo Fisher AM9260G)) in a 5 mL FACS tube and washed 3 times. The cells were stained with fixable live/dead aqua prior to antibody staining. Cell examined for surface TLR1 were stained prior to fixation with 1:100 primary antibody in FACS buffer for 1 hour at room temperature protected from light. They were then washed twice in FACS buffer.

Cells examined for intracellular TLR1 staining were live/dead stained, washed and then taken through the intracellular staining protocol. The intracellular staining kit protocol was followed to stain intracellular TLR1 with a primary antibody incubation at 1:100 in FACS buffer for 1 hour at room temperature protected from light. The cells were then washed twice and resuspended in 300 µL FACS buffer.

### RT-qPCR

RNA was collected by homogenizing cells in 700 µL of Qiazol (Qiagen 79306). RNA was isolated using the miRNeasy isolation kit (Qiagen 217084). cDNA was synthesized from isolated RNA using the ImProm-II Reverse Transcriptase kit (Promega A3801). qPCR was completed using the iTaq Universal SYBR Green Supermix (Biorad 1725124). Reactions were performed using a Biorad CFX Connect Real-Time System. Cycling parameters were one cycle of 95°C for 15 minutes, 40 cycles of 95°C for 30 seconds, 60°C for 30 seconds, and 72°C for 30 seconds and one cycle of 95°C for 1 minute and at 55°C for 1 minute. Genes evaluated for induction or knock down were compared to human β-actin (Table 1).

**Table 1:** Oligonucleotides used in this study.

Primer Target	Forward	Reverse	Source
*IFNB1*	5’-CTTACAGGTTACCTCCGAAACTGAA-3’	5’-GGTTGAAGAATGCTTGAAGCAA- 3’	[49]
*MYD88*	5’-ATGGTGGTGGTTGTCTCTGATG-3’	5’-GACAGTGATGAACCTCAGGATGC-3’	[50]
*TICAM1*	5’-ACGCCATAGACCACTCAGCTTTCA-3’	5’-AGGTTGCTCATCATGGCTTGGTTC-3’	[50]
*TICAM2*	5’-AGATGAAGCCCTCAGAGTCCAG-3’	5’-CTGTAAATGCTGTCTGCCACATG-3’	[50]
*β-actin*	5’-CCACACCTTCTACAATGAGCTGCG-3’	5’-CGGAGTCCATCACGATGCCA-3’	[51]

## Supporting information

S1 Raw Images Dual channel images of uncropped western blots in Fig 3 and Fig 4. (PDF)

S1 Fig HEK Blue TLR1/6KO cells transfected with TLR1 variants activate NF-κB through TLR3, but do not transcribe *IFNβ.* (A) Percent change of NF-κB p65 SEAP readout from cells stimulated with 100 ng/mL Pam3CSK4 or 50 ng/mL Poly I:C for 18 hours. (B) Relative expression of *IFNB1* from cells stimulated for 6 hours.
(TIF)

S2 Fig HEK Blue TLR1/6KO cells transfected with TLR1 variants express minimal TRAM/TRIF. Relative expression of *MyD88*, *TICAM2* (TRAM), and *TICAM1* (TRIF) in HEK Blue cells transfected with plasmid vector, 602I, or 602S TLR1 variants.
(TIF)

S1 File Plasmid sequences for TLR1 variant expression constructs. Sequences include Addgene pCMV-3Tag-3a, pCMV-TLR1.602I-3FLAG, and pCMV-TLR1.602S-3FLAG.
(DOCX)
