# Supplementary material for: Endosome restricted human TLR1 polymorphism highlights a role for MyD88 in Pam3CSK4 detection
Source: PLoS One. 2026 Oct 5;21(10):e0358252. doi: 10.1371/journal.pone.0358252 (PMC13638051; doi:10.1371/journal.pone.0358252)
Supplement: Supplementary file 1 [file pone.0358252.s001.pdf]

Fig3A Representative and Fig3B n1
Channel 700

Licor Odyssey F Imager

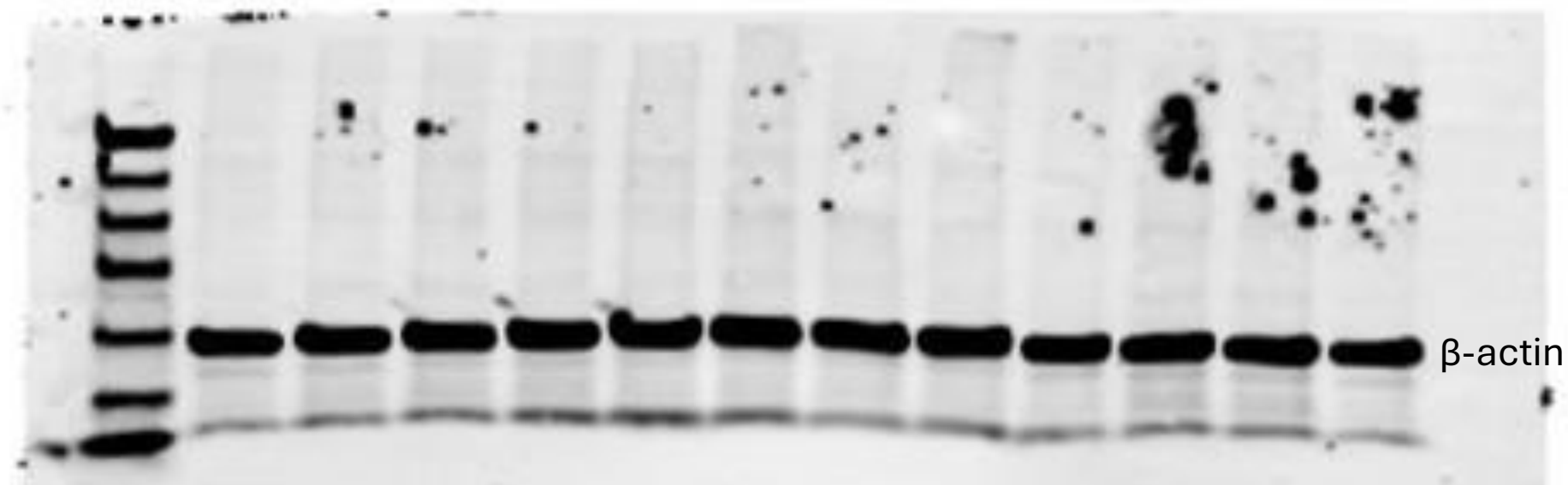

Lane #	Contents
1	Licor Chameleon Duo Ladder
2	Whole Cell Lysate HEK Blue TLR1 602S – Unstimulated n1
3	Whole Cell Lysate HEK Blue TLR1 602S – 30” 100 ng/mL Pam3CSK4 Stimulated n1
4	Whole Cell Lysate HEK Blue TLR1 602S – 60” 100 ng/mL Pam3CSK4 Stimulated n1
5	Whole Cell Lysate HEK Blue TLR1 602S – 90” 100 ng/mL Pam3CSK4 Stimulated n1
6	Whole Cell Lysate HEK Blue TLR1 602S – 120” 100 ng/mL Pam3CSK4 Stimulated n1
7	Whole Cell Lysate HEK Blue TLR1 602S – 180” 100 ng/mL Pam3CSK4 Stimulated n1
8	Whole Cell Lysate HEK Blue TLR1 602I – Unstimulated n1
9	Whole Cell Lysate HEK Blue TLR1 602I – 30” 100 ng/mL Pam3CSK4 Stimulated n1
10	Whole Cell Lysate HEK Blue TLR1 602I – 60” 100 ng/mL Pam3CSK4 Stimulated n1
11	Whole Cell Lysate HEK Blue TLR1 602I – 90” 100 ng/mL Pam3CSK4 Stimulated n1
12	Whole Cell Lysate HEK Blue TLR1 602I – 120” 100 ng/mL Pam3CSK4 Stimulated n1
13	Whole Cell Lysate HEK Blue TLR1 602I – 180” 100 ng/mL Pam3CSK4 Stimulated n1

Fig3A Representative and Fig3B n1
Channel 800

Licor Odyssey F Imager

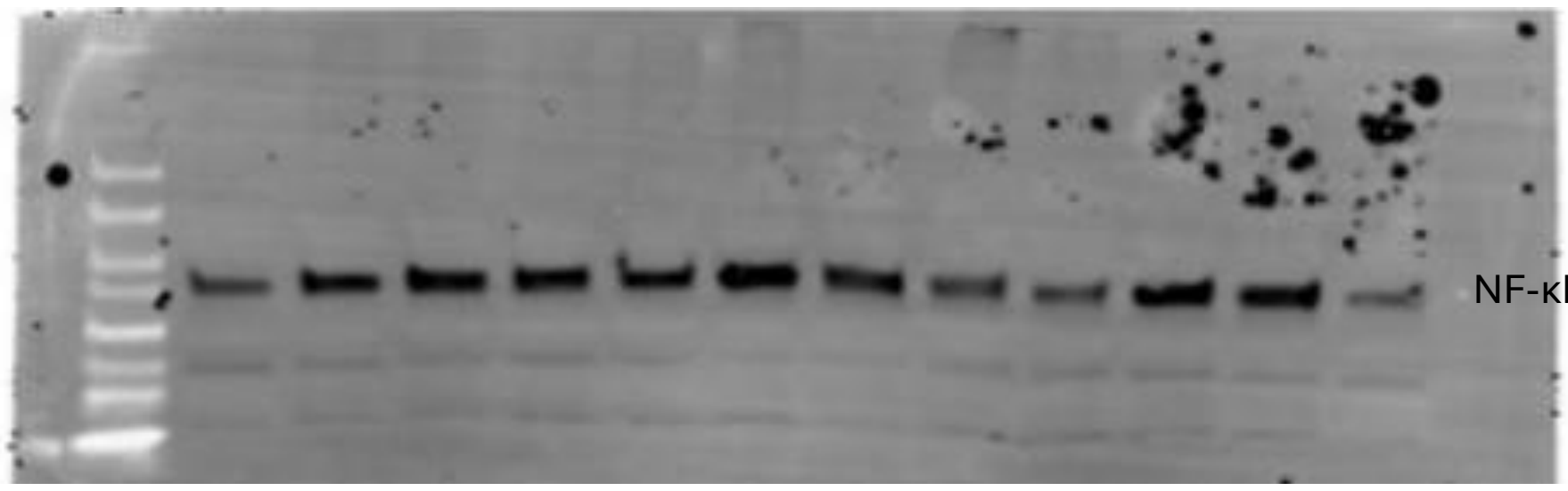

Lane #	Contents
1	Licor Chameleon Duo Ladder
2	Whole Cell Lysate HEK Blue TLR1 602S – Unstimulated n1
3	Whole Cell Lysate HEK Blue TLR1 602S – 30” 100 ng/mL Pam3CSK4 Stimulated n1
4	Whole Cell Lysate HEK Blue TLR1 602S – 60” 100 ng/mL Pam3CSK4 Stimulated n1
5	Whole Cell Lysate HEK Blue TLR1 602S – 90” 100 ng/mL Pam3CSK4 Stimulated n1
6	Whole Cell Lysate HEK Blue TLR1 602S – 120” 100 ng/mL Pam3CSK4 Stimulated n1
7	Whole Cell Lysate HEK Blue TLR1 602S – 180” 100 ng/mL Pam3CSK4 Stimulated n1
8	Whole Cell Lysate HEK Blue TLR1 602I – Unstimulated n1
9	Whole Cell Lysate HEK Blue TLR1 602I – 30” 100 ng/mL Pam3CSK4 Stimulated n1
10	Whole Cell Lysate HEK Blue TLR1 602I – 60” 100 ng/mL Pam3CSK4 Stimulated n1
11	Whole Cell Lysate HEK Blue TLR1 602I – 90” 100 ng/mL Pam3CSK4 Stimulated n1
12	Whole Cell Lysate HEK Blue TLR1 602I – 120” 100 ng/mL Pam3CSK4 Stimulated n1
13	Whole Cell Lysate HEK Blue TLR1 602I – 180” 100 ng/mL Pam3CSK4 Stimulated n1

Fig3A Representative Channel 700

Licor Odyssey F Imager

IkBα

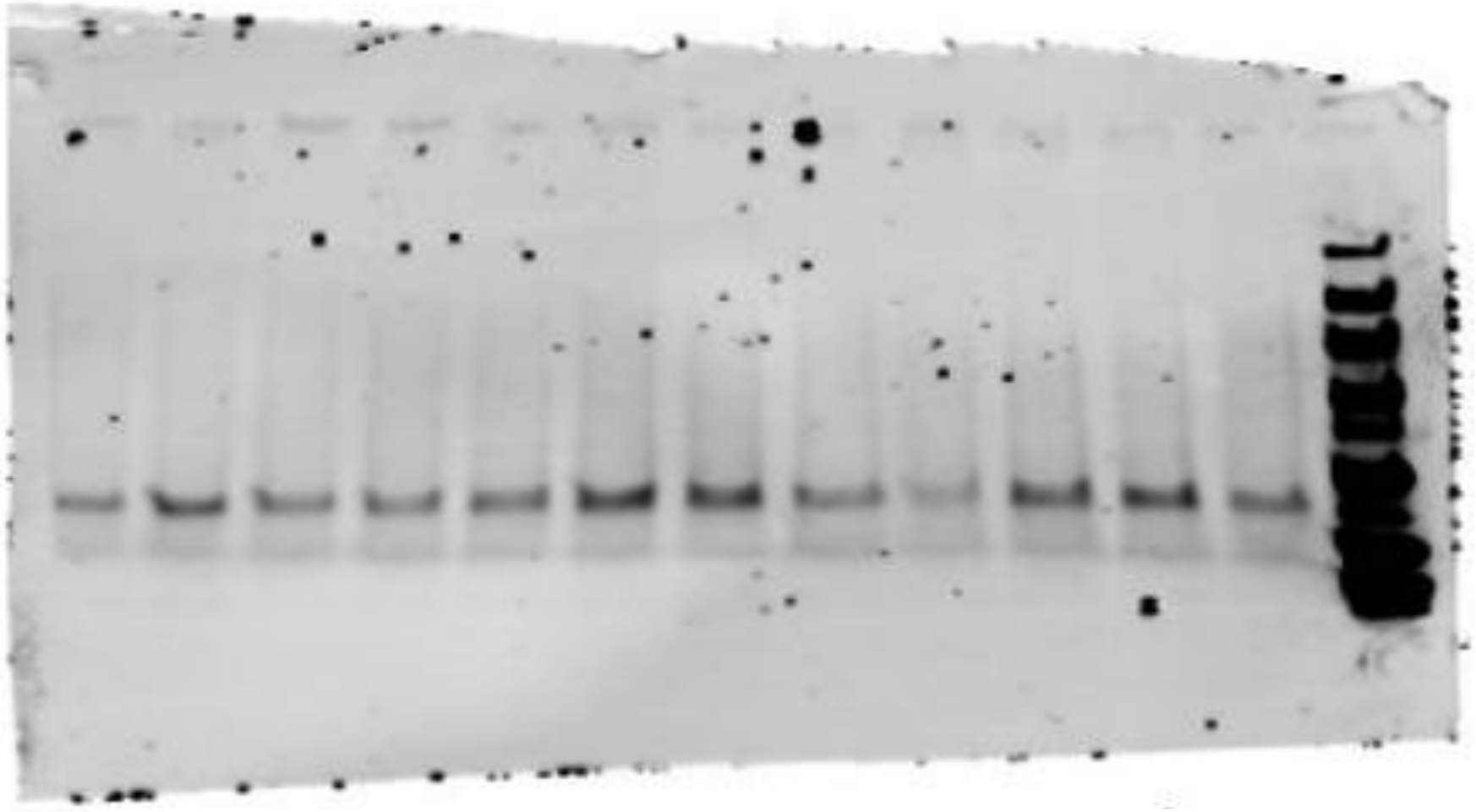

Lane #	Contents
1	Whole Cell Lysate HEK Blue TLR1 602S – Unstimulated n1
2	Whole Cell Lysate HEK Blue TLR1 602S – 30” 100 ng/mL Pam3CSK4 Stimulated n1
3	Whole Cell Lysate HEK Blue TLR1 602S – 60” 100 ng/mL Pam3CSK4 Stimulated n1
4	Whole Cell Lysate HEK Blue TLR1 602S – 90” 100 ng/mL Pam3CSK4 Stimulated n1
5	Whole Cell Lysate HEK Blue TLR1 602S – 120” 100 ng/mL Pam3CSK4 Stimulated n1
6	Whole Cell Lysate HEK Blue TLR1 602S – 180” 100 ng/mL Pam3CSK4 Stimulated n1
7	Whole Cell Lysate HEK Blue TLR1 602I - Unstimulated n1
8	Whole Cell Lysate HEK Blue TLR1 602I – 30” 100 ng/mL Pam3CSK4 Stimulated n1
9	Whole Cell Lysate HEK Blue TLR1 602I – 60” 100 ng/mL Pam3CSK4 Stimulated n1
10	Whole Cell Lysate HEK Blue TLR1 602I – 90” 100 ng/mL Pam3CSK4 Stimulated n1
11	Whole Cell Lysate HEK Blue TLR1 602I – 120” 100 ng/mL Pam3CSK4 Stimulated n1
12	Whole Cell Lysate HEK Blue TLR1 602I – 180” 100 ng/mL Pam3CSK4 Stimulated n1
13	Licor Chameleon Duo Ladder

Fig3A Representative
Channel 800

Licor Odyssey F Imager

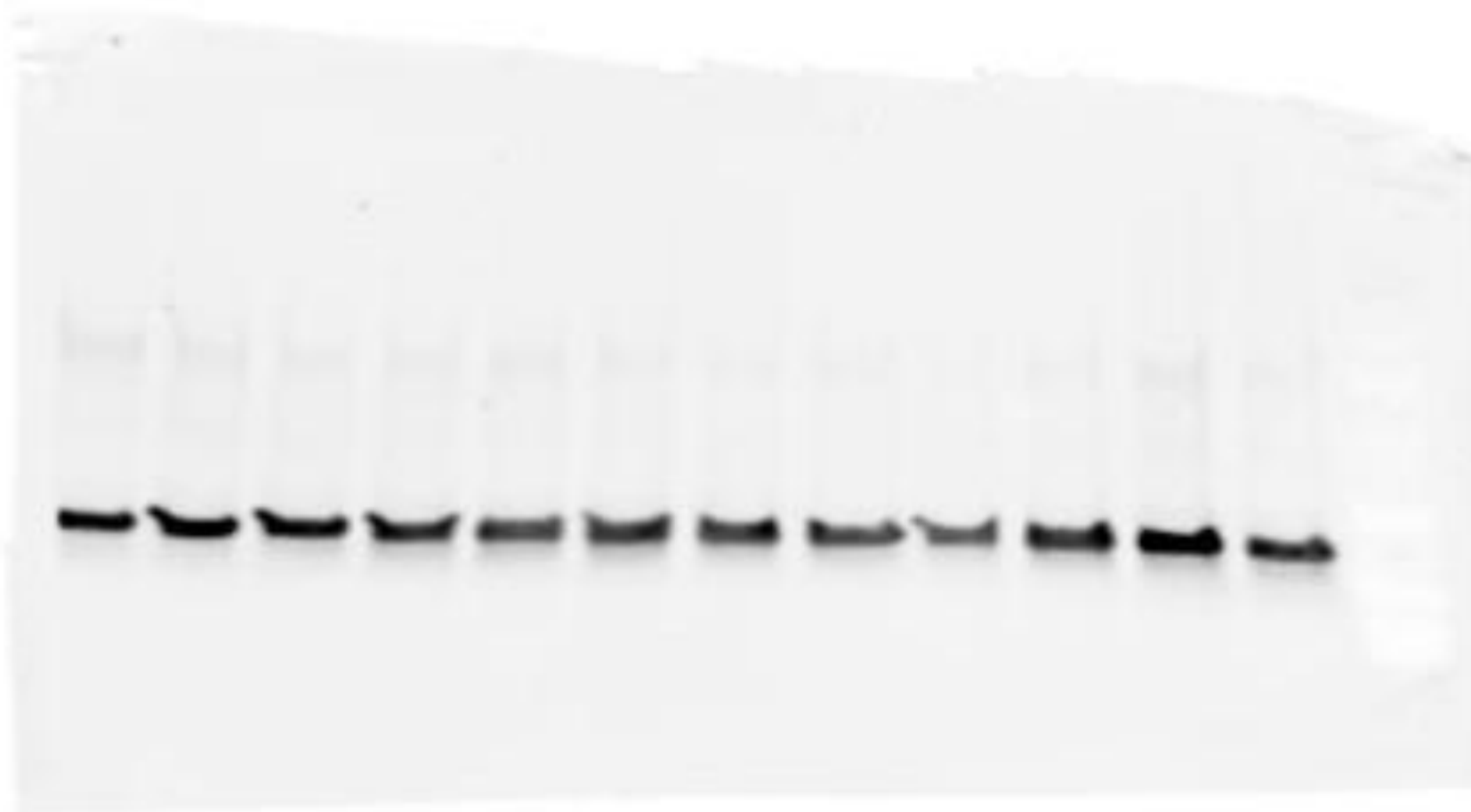

Lane #	Contents
1	Whole Cell Lysate HEK Blue TLR1 602S – Unstimulated n1
2	Whole Cell Lysate HEK Blue TLR1 602S – 30” 100 ng/mL Pam3CSK4 Stimulated n1
3	Whole Cell Lysate HEK Blue TLR1 602S – 60” 100 ng/mL Pam3CSK4 Stimulated n1
4	Whole Cell Lysate HEK Blue TLR1 602S – 90” 100 ng/mL Pam3CSK4 Stimulated n1
5	Whole Cell Lysate HEK Blue TLR1 602S – 120” 100 ng/mL Pam3CSK4 Stimulated n1
6	Whole Cell Lysate HEK Blue TLR1 602S – 180” 100 ng/mL Pam3CSK4 Stimulated n1
7	Whole Cell Lysate HEK Blue TLR1 602I - Unstimulated n1
8	Whole Cell Lysate HEK Blue TLR1 602I – 30” 100 ng/mL Pam3CSK4 Stimulated n1
9	Whole Cell Lysate HEK Blue TLR1 602I – 60” 100 ng/mL Pam3CSK4 Stimulated n1
10	Whole Cell Lysate HEK Blue TLR1 602I – 90” 100 ng/mL Pam3CSK4 Stimulated n1
11	Whole Cell Lysate HEK Blue TLR1 602I – 120” 100 ng/mL Pam3CSK4 Stimulated n1
12	Whole Cell Lysate HEK Blue TLR1 602I – 180” 100 ng/mL Pam3CSK4 Stimulated n1
13	Licor Chameleon Duo Ladder

Fig3B n2
Channel 700

Licor Odyssey F Imager

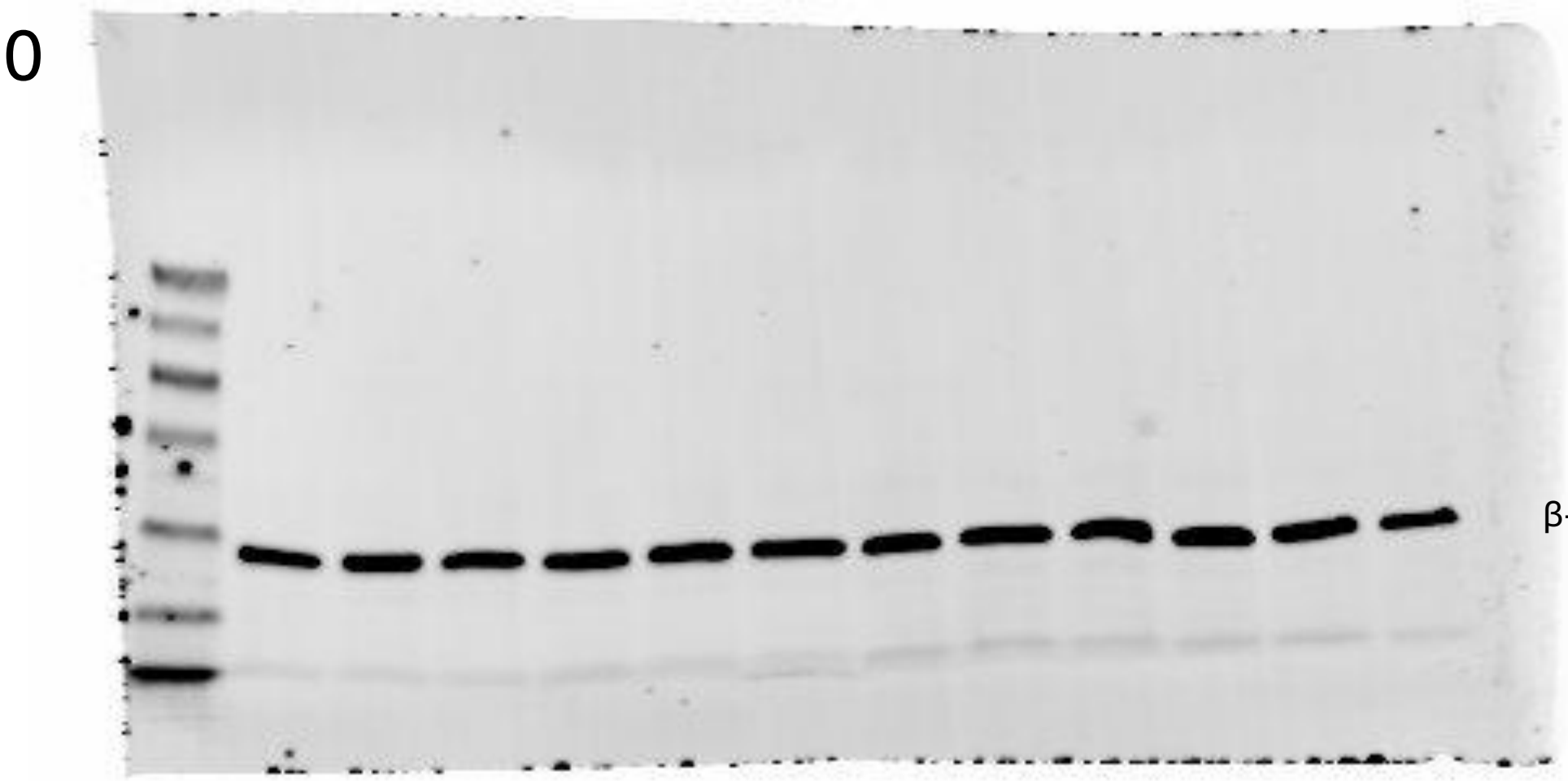

Lane #	Contents
1	Licor Chameleon Duo Ladder
2	Whole Cell Lysate HEK Blue TLR1 602S – Unstimulated n2
3	Whole Cell Lysate HEK Blue TLR1 602S – 30” 100 ng/mL Pam3CSK4 Stimulated n2
4	Whole Cell Lysate HEK Blue TLR1 602S – 60” 100 ng/mL Pam3CSK4 Stimulated n2
5	Whole Cell Lysate HEK Blue TLR1 602S – 90” 100 ng/mL Pam3CSK4 Stimulated n2
6	Whole Cell Lysate HEK Blue TLR1 602S – 120” 100 ng/mL Pam3CSK4 Stimulated n2
7	Whole Cell Lysate HEK Blue TLR1 602S – 180” 100 ng/mL Pam3CSK4 Stimulated n2
8	Whole Cell Lysate HEK Blue TLR1 602I – Unstimulated n2
9	Whole Cell Lysate HEK Blue TLR1 602I – 30” 100 ng/mL Pam3CSK4 Stimulated n2
10	Whole Cell Lysate HEK Blue TLR1 602I – 60” 100 ng/mL Pam3CSK4 Stimulated n2
11	Whole Cell Lysate HEK Blue TLR1 602I – 90” 100 ng/mL Pam3CSK4 Stimulated n2
12	Whole Cell Lysate HEK Blue TLR1 602I – 120” 100 ng/mL Pam3CSK4 Stimulated n2
13	Whole Cell Lysate HEK Blue TLR1 602I – 180” 100 ng/mL Pam3CSK4 Stimulated n2

Fig3B n2
Channel 800

Licor Odyssey F Imager

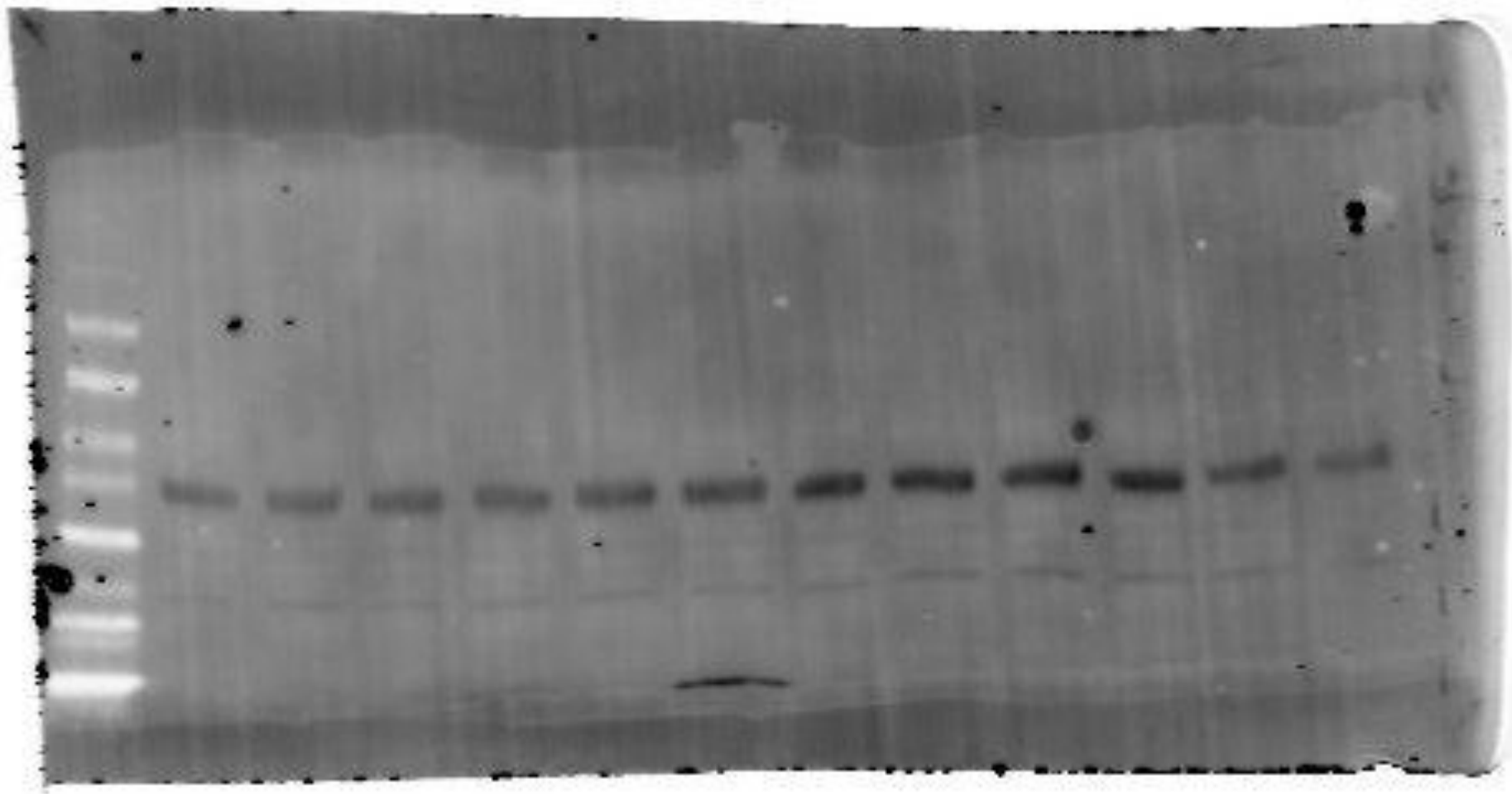

Lane #	Contents
1	Licor Chameleon Duo Ladder
2	Whole Cell Lysate HEK Blue TLR1 602S – Unstimulated n2
3	Whole Cell Lysate HEK Blue TLR1 602S – 30” 100 ng/mL Pam3CSK4 Stimulated n2
4	Whole Cell Lysate HEK Blue TLR1 602S – 60” 100 ng/mL Pam3CSK4 Stimulated n2
5	Whole Cell Lysate HEK Blue TLR1 602S – 90” 100 ng/mL Pam3CSK4 Stimulated n2
6	Whole Cell Lysate HEK Blue TLR1 602S – 120” 100 ng/mL Pam3CSK4 Stimulated n2
7	Whole Cell Lysate HEK Blue TLR1 602S – 180” 100 ng/mL Pam3CSK4 Stimulated n2
8	Whole Cell Lysate HEK Blue TLR1 602I – Unstimulated n2
9	Whole Cell Lysate HEK Blue TLR1 602I – 30” 100 ng/mL Pam3CSK4 Stimulated n2
10	Whole Cell Lysate HEK Blue TLR1 602I – 60” 100 ng/mL Pam3CSK4 Stimulated n2
11	Whole Cell Lysate HEK Blue TLR1 602I – 90” 100 ng/mL Pam3CSK4 Stimulated n2
12	Whole Cell Lysate HEK Blue TLR1 602I – 120” 100 ng/mL Pam3CSK4 Stimulated n2
13	Whole Cell Lysate HEK Blue TLR1 602I – 180” 100 ng/mL Pam3CSK4 Stimulated n2

Fig3B n3
Channel 700

Licor Odyssey F Imager

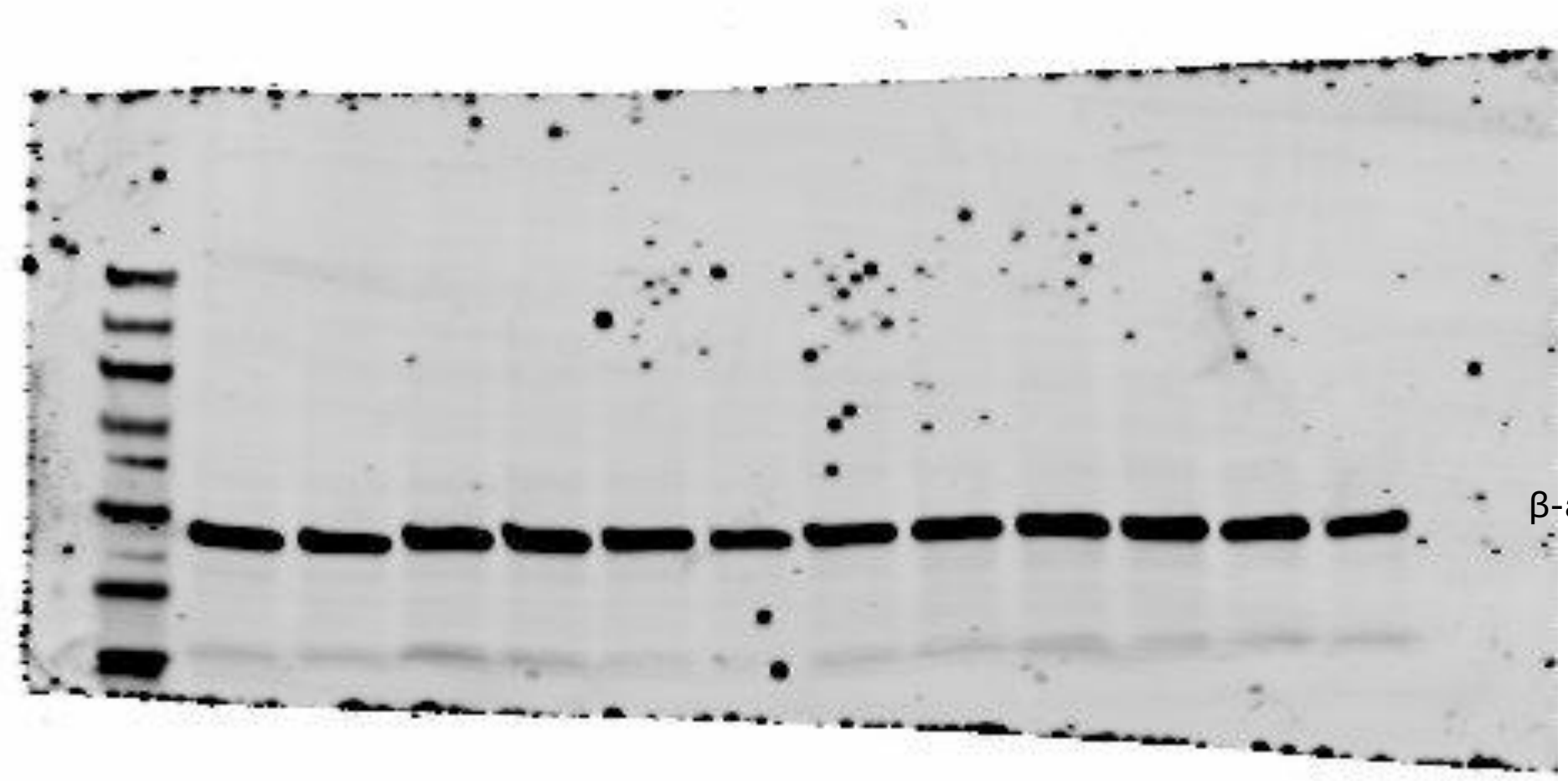

Lane #	Contents
1	Licor Chameleon Duo Ladder
2	Whole Cell Lysate HEK Blue TLR1 602S – Unstimulated n3
3	Whole Cell Lysate HEK Blue TLR1 602S – 30” 100 ng/mL Pam3CSK4 Stimulated n3
4	Whole Cell Lysate HEK Blue TLR1 602S – 60” 100 ng/mL Pam3CSK4 Stimulated n3
5	Whole Cell Lysate HEK Blue TLR1 602S – 90” 100 ng/mL Pam3CSK4 Stimulated n3
6	Whole Cell Lysate HEK Blue TLR1 602S – 120” 100 ng/mL Pam3CSK4 Stimulated n3
7	Whole Cell Lysate HEK Blue TLR1 602S – 180” 100 ng/mL Pam3CSK4 Stimulated n3
8	Whole Cell Lysate HEK Blue TLR1 602I – Unstimulated n3
9	Whole Cell Lysate HEK Blue TLR1 602I – 30” 100 ng/mL Pam3CSK4 Stimulated n3
10	Whole Cell Lysate HEK Blue TLR1 602I – 60” 100 ng/mL Pam3CSK4 Stimulated n3
11	Whole Cell Lysate HEK Blue TLR1 602I – 90” 100 ng/mL Pam3CSK4 Stimulated n3
12	Whole Cell Lysate HEK Blue TLR1 602I – 120” 100 ng/mL Pam3CSK4 Stimulated n3
13	Whole Cell Lysate HEK Blue TLR1 602I – 180” 100 ng/mL Pam3CSK4 Stimulated n3

Fig3B n3
Channel 800

Licor Odyssey F Imager

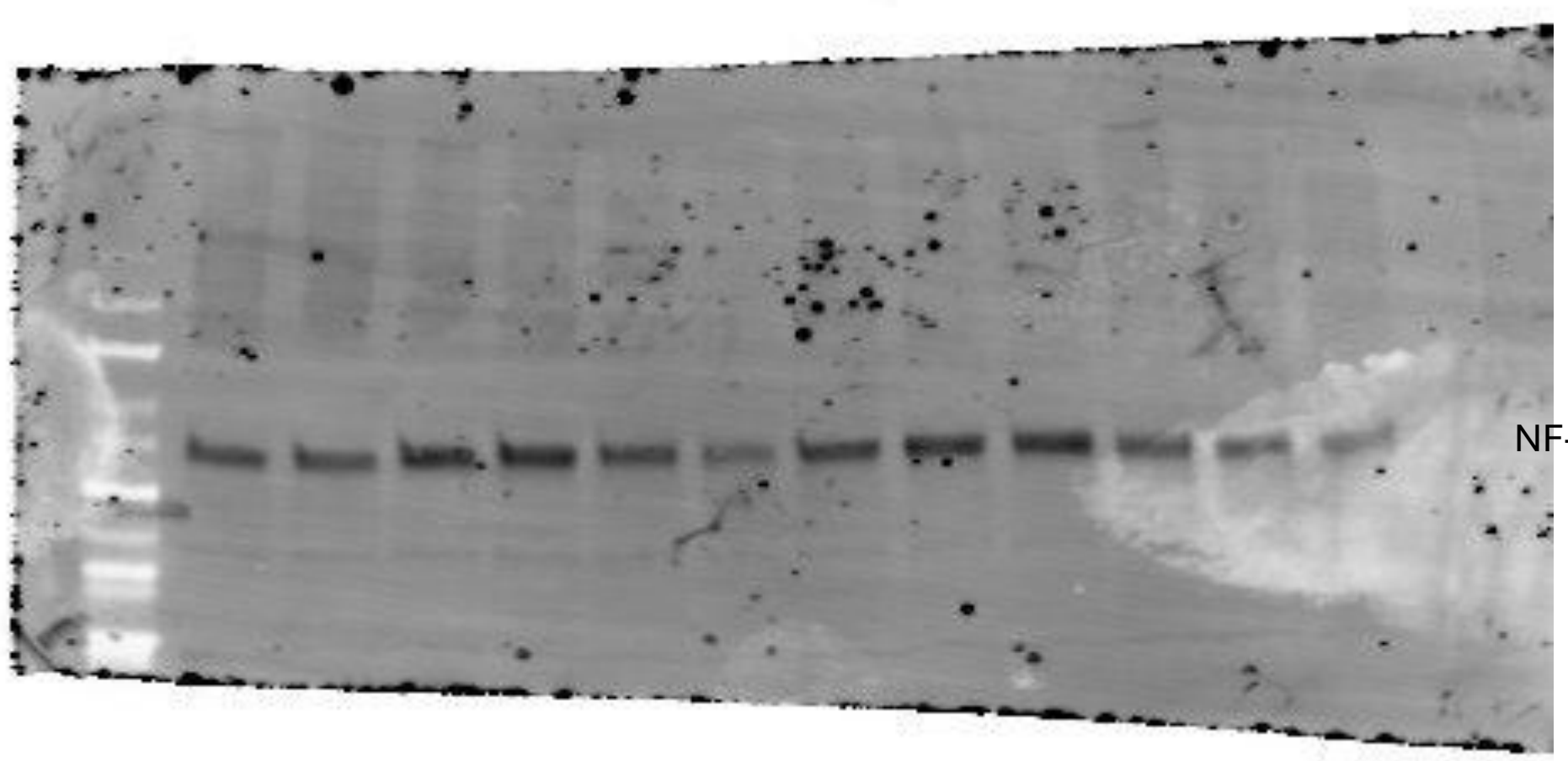

Lane #	Contents
1	Licor Chameleon Duo Ladder
2	Whole Cell Lysate HEK Blue TLR1 602S – Unstimulated n3
3	Whole Cell Lysate HEK Blue TLR1 602S – 30” 100 ng/mL Pam3CSK4 Stimulated n3
4	Whole Cell Lysate HEK Blue TLR1 602S – 60” 100 ng/mL Pam3CSK4 Stimulated n3
5	Whole Cell Lysate HEK Blue TLR1 602S – 90” 100 ng/mL Pam3CSK4 Stimulated n3
6	Whole Cell Lysate HEK Blue TLR1 602S – 120” 100 ng/mL Pam3CSK4 Stimulated n3
7	Whole Cell Lysate HEK Blue TLR1 602S – 180” 100 ng/mL Pam3CSK4 Stimulated n3
8	Whole Cell Lysate HEK Blue TLR1 602I – Unstimulated n3
9	Whole Cell Lysate HEK Blue TLR1 602I – 30” 100 ng/mL Pam3CSK4 Stimulated n3
10	Whole Cell Lysate HEK Blue TLR1 602I – 60” 100 ng/mL Pam3CSK4 Stimulated n3
11	Whole Cell Lysate HEK Blue TLR1 602I – 90” 100 ng/mL Pam3CSK4 Stimulated n3
12	Whole Cell Lysate HEK Blue TLR1 602I – 120” 100 ng/mL Pam3CSK4 Stimulated n3
13	Whole Cell Lysate HEK Blue TLR1 602I – 180” 100 ng/mL Pam3CSK4 Stimulated n3

Fig3C n2(Top) and n3 (bottom)
Channel 700

Licor Odyssey
F Imager

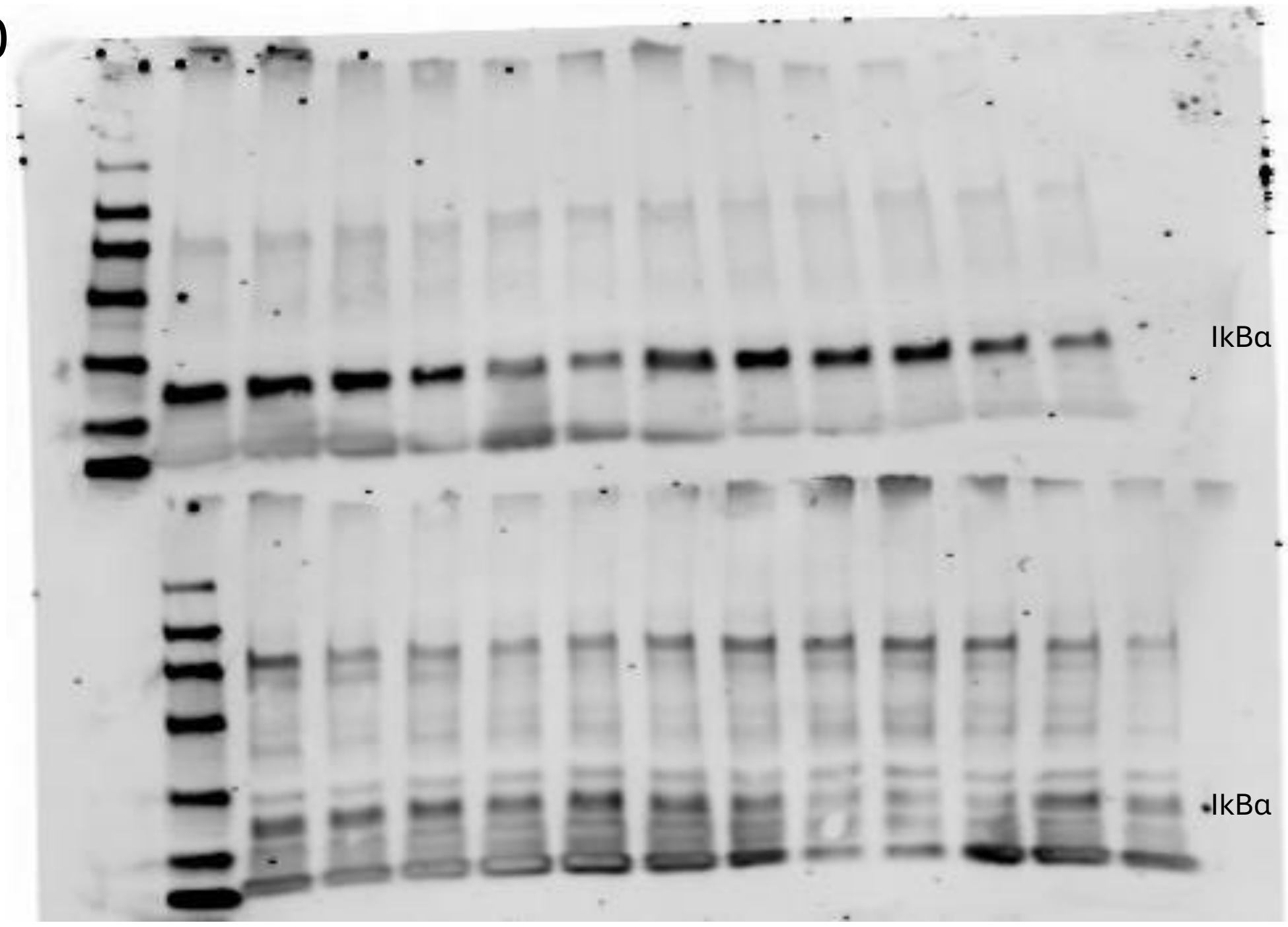

Lane #	Contents (n2)
1	Licor Chameleon Duo Ladder
2	Whole Cell Lysate HEK Blue TLR1 602S – Unstimulated n2
3	Whole Cell Lysate HEK Blue TLR1 602S – 30” 100 ng/mL Pam3CSK4 Stimulated n2
4	Whole Cell Lysate HEK Blue TLR1 602S – 60” 100 ng/mL Pam3CSK4 Stimulated n2
5	Whole Cell Lysate HEK Blue TLR1 602S – 90” 100 ng/mL Pam3CSK4 Stimulated n2
6	Whole Cell Lysate HEK Blue TLR1 602S – 120” 100 ng/mL Pam3CSK4 Stimulated n2
7	Whole Cell Lysate HEK Blue TLR1 602S – 180” 100 ng/mL Pam3CSK4 Stimulated n2
8	Whole Cell Lysate HEK Blue TLR1 602I – Unstimulated n2
9	Whole Cell Lysate HEK Blue TLR1 602I – 30” 100 ng/mL Pam3CSK4 Stimulated n2
10	Whole Cell Lysate HEK Blue TLR1 602I – 60” 100 ng/mL Pam3CSK4 Stimulated n2
11	Whole Cell Lysate HEK Blue TLR1 602I – 90” 100 ng/mL Pam3CSK4 Stimulated n2
12	Whole Cell Lysate HEK Blue TLR1 602I – 120” 100 ng/mL Pam3CSK4 Stimulated n2
13	Whole Cell Lysate HEK Blue TLR1 602I – 180” 100 ng/mL Pam3CSK4 Stimulated n2

Lane #	Contents (n3)
1	Licor Chameleon Duo Ladder
2	Whole Cell Lysate HEK Blue TLR1 602S – Unstimulated n3
3	Whole Cell Lysate HEK Blue TLR1 602S – 30” 100 ng/mL Pam3CSK4 Stimulated n3
4	Whole Cell Lysate HEK Blue TLR1 602S – 60” 100 ng/mL Pam3CSK4 Stimulated n3
5	Whole Cell Lysate HEK Blue TLR1 602S – 90” 100 ng/mL Pam3CSK4 Stimulated n3
6	Whole Cell Lysate HEK Blue TLR1 602S – 120” 100 ng/mL Pam3CSK4 Stimulated n3
7	Whole Cell Lysate HEK Blue TLR1 602S – 180” 100 ng/mL Pam3CSK4 Stimulated n3
8	Whole Cell Lysate HEK Blue TLR1 602I – Unstimulated n3
9	Whole Cell Lysate HEK Blue TLR1 602I – 30” 100 ng/mL Pam3CSK4 Stimulated n3
10	Whole Cell Lysate HEK Blue TLR1 602I – 60” 100 ng/mL Pam3CSK4 Stimulated n3
11	Whole Cell Lysate HEK Blue TLR1 602I – 90” 100 ng/mL Pam3CSK4 Stimulated n3
12	Whole Cell Lysate HEK Blue TLR1 602I – 120” 100 ng/mL Pam3CSK4 Stimulated n3
13	Whole Cell Lysate HEK Blue TLR1 602I – 180” 100 ng/mL Pam3CSK4 Stimulated n3

Fig3C n2(Top) and n3 (bottom)
Channel 800

Licor Odyssey
F Imager

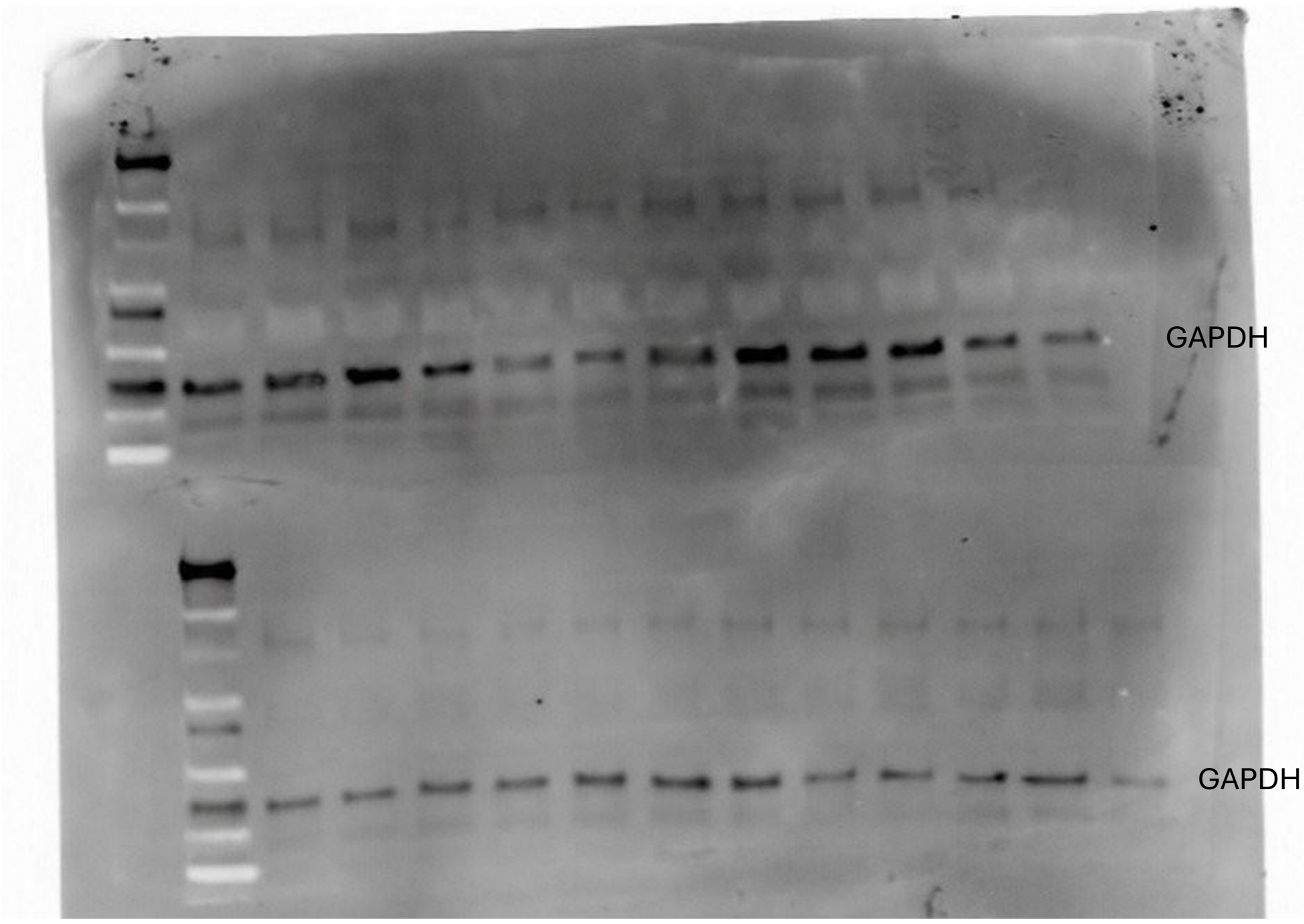

Lane #	Contents (n2)
1	Licor Chameleon Duo Ladder
2	Whole Cell Lysate HEK Blue TLR1 602S – Unstimulated n2
3	Whole Cell Lysate HEK Blue TLR1 602S – 30” 100 ng/mL Pam3CSK4 Stimulated n2
4	Whole Cell Lysate HEK Blue TLR1 602S – 60” 100 ng/mL Pam3CSK4 Stimulated n2
5	Whole Cell Lysate HEK Blue TLR1 602S – 90” 100 ng/mL Pam3CSK4 Stimulated n2
6	Whole Cell Lysate HEK Blue TLR1 602S – 120” 100 ng/mL Pam3CSK4 Stimulated n2
7	Whole Cell Lysate HEK Blue TLR1 602S – 180” 100 ng/mL Pam3CSK4 Stimulated n2
8	Whole Cell Lysate HEK Blue TLR1 602I – Unstimulated n2
9	Whole Cell Lysate HEK Blue TLR1 602I – 30” 100 ng/mL Pam3CSK4 Stimulated n2
10	Whole Cell Lysate HEK Blue TLR1 602I – 60” 100 ng/mL Pam3CSK4 Stimulated n2
11	Whole Cell Lysate HEK Blue TLR1 602I – 90” 100 ng/mL Pam3CSK4 Stimulated n2
12	Whole Cell Lysate HEK Blue TLR1 602I – 120” 100 ng/mL Pam3CSK4 Stimulated n2
13	Whole Cell Lysate HEK Blue TLR1 602I – 180” 100 ng/mL Pam3CSK4 Stimulated n2

Lane #	Contents (n3)
1	Licor Chameleon Duo Ladder
2	Whole Cell Lysate HEK Blue TLR1 602S – Unstimulated n3
3	Whole Cell Lysate HEK Blue TLR1 602S – 30” 100 ng/mL Pam3CSK4 Stimulated n3
4	Whole Cell Lysate HEK Blue TLR1 602S – 60” 100 ng/mL Pam3CSK4 Stimulated n3
5	Whole Cell Lysate HEK Blue TLR1 602S – 90” 100 ng/mL Pam3CSK4 Stimulated n3
6	Whole Cell Lysate HEK Blue TLR1 602S – 120” 100 ng/mL Pam3CSK4 Stimulated n3
7	Whole Cell Lysate HEK Blue TLR1 602S – 180” 100 ng/mL Pam3CSK4 Stimulated n3
8	Whole Cell Lysate HEK Blue TLR1 602I – Unstimulated n3
9	Whole Cell Lysate HEK Blue TLR1 602I – 30” 100 ng/mL Pam3CSK4 Stimulated n3
10	Whole Cell Lysate HEK Blue TLR1 602I – 60” 100 ng/mL Pam3CSK4 Stimulated n3
11	Whole Cell Lysate HEK Blue TLR1 602I – 90” 100 ng/mL Pam3CSK4 Stimulated n3
12	Whole Cell Lysate HEK Blue TLR1 602I – 120” 100 ng/mL Pam3CSK4 Stimulated n3
13	Whole Cell Lysate HEK Blue TLR1 602I – 180” 100 ng/mL Pam3CSK4 Stimulated n3

Fig3D Representative and Fig3E n1
Channel 700

Licor Odyssey F Imager

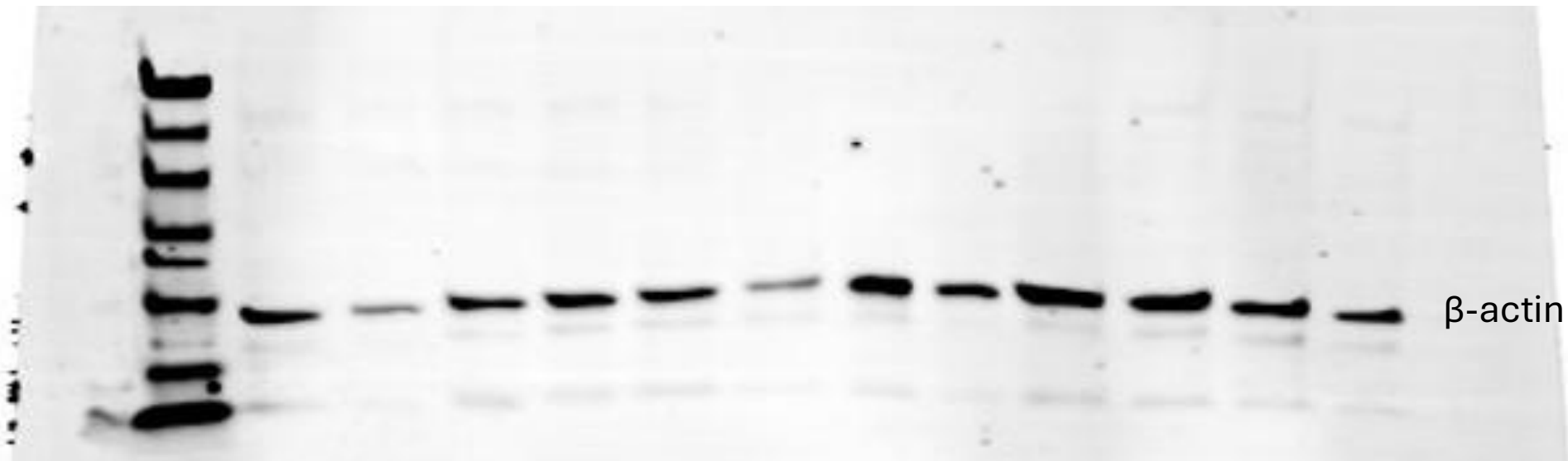

Lane #	Contents
1	Licor Chameleon Duo Ladder
2	Nuclear Enriched Lysate HEK Blue TLR1 602S – Unstimulated n1
3	Nuclear Enriched Lysate HEK Blue TLR1 602S – 30” 100 ng/mL Pam3CSK4 Stimulated n1
4	Nuclear Enriched Lysate HEK Blue TLR1 602S – 60” 100 ng/mL Pam3CSK4 Stimulated n1
5	Nuclear Enriched Lysate HEK Blue TLR1 602S – 90” 100 ng/mL Pam3CSK4 Stimulated n1
6	Nuclear Enriched Lysate HEK Blue TLR1 602S – 120” 100 ng/mL Pam3CSK4 Stimulated n1
7	Nuclear Enriched Lysate HEK Blue TLR1 602S – 180” 100 ng/mL Pam3CSK4 Stimulated n1
8	Nuclear Enriched Lysate HEK Blue TLR1 602I – Unstimulated n1
9	Nuclear Enriched Lysate HEK Blue TLR1 602I – 30” 100 ng/mL Pam3CSK4 Stimulated n1
10	Nuclear Enriched Lysate HEK Blue TLR1 602I – 60” 100 ng/mL Pam3CSK4 Stimulated n1
11	Nuclear Enriched Lysate HEK Blue TLR1 602I – 90” 100 ng/mL Pam3CSK4 Stimulated n1
12	Nuclear Enriched Lysate HEK Blue TLR1 602I – 120” 100 ng/mL Pam3CSK4 Stimulated n1
13	Nuclear Enriched Lysate HEK Blue TLR1 602I – 180” 100 ng/mL Pam3CSK4 Stimulated n1

Fig3D Representative and Fig3E n1

Channel 800

Licor Odyssey F Imager

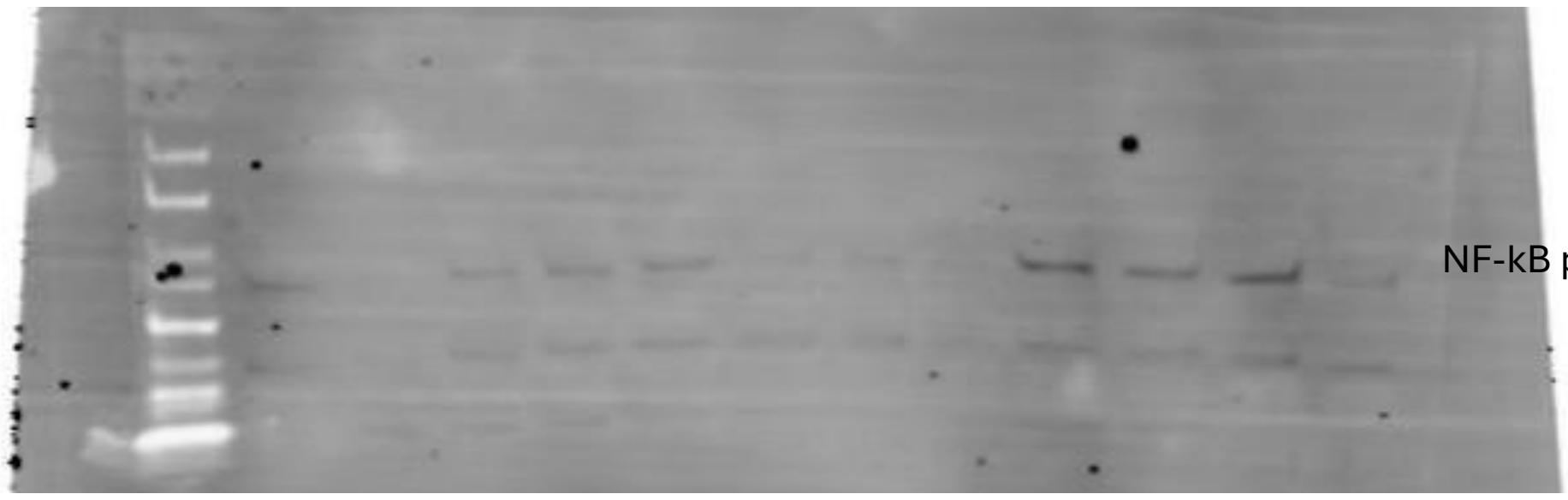

Lane #	Contents
1	Licor Chameleon Duo Ladder
2	Nuclear Enriched Lysate HEK Blue TLR1 602S – Unstimulated n1
3	Nuclear Enriched Lysate HEK Blue TLR1 602S – 30” 100 ng/mL Pam3CSK4 Stimulated n1
4	Nuclear Enriched Lysate HEK Blue TLR1 602S – 60” 100 ng/mL Pam3CSK4 Stimulated n1
5	Nuclear Enriched Lysate HEK Blue TLR1 602S – 90” 100 ng/mL Pam3CSK4 Stimulated n1
6	Nuclear Enriched Lysate HEK Blue TLR1 602S – 120” 100 ng/mL Pam3CSK4 Stimulated n1
7	Nuclear Enriched Lysate HEK Blue TLR1 602S – 180” 100 ng/mL Pam3CSK4 Stimulated n1
8	Nuclear Enriched Lysate HEK Blue TLR1 602I – Unstimulated n1
9	Nuclear Enriched Lysate HEK Blue TLR1 602I – 30” 100 ng/mL Pam3CSK4 Stimulated n1
10	Nuclear Enriched Lysate HEK Blue TLR1 602I – 60” 100 ng/mL Pam3CSK4 Stimulated n1
11	Nuclear Enriched Lysate HEK Blue TLR1 602I – 90” 100 ng/mL Pam3CSK4 Stimulated n1
12	Nuclear Enriched Lysate HEK Blue TLR1 602I – 120” 100 ng/mL Pam3CSK4 Stimulated n1
13	Nuclear Enriched Lysate HEK Blue TLR1 602I – 180” 100 ng/mL Pam3CSK4 Stimulated n1

Fig3D Representative Channel 700

Licor Odyssey F Imager

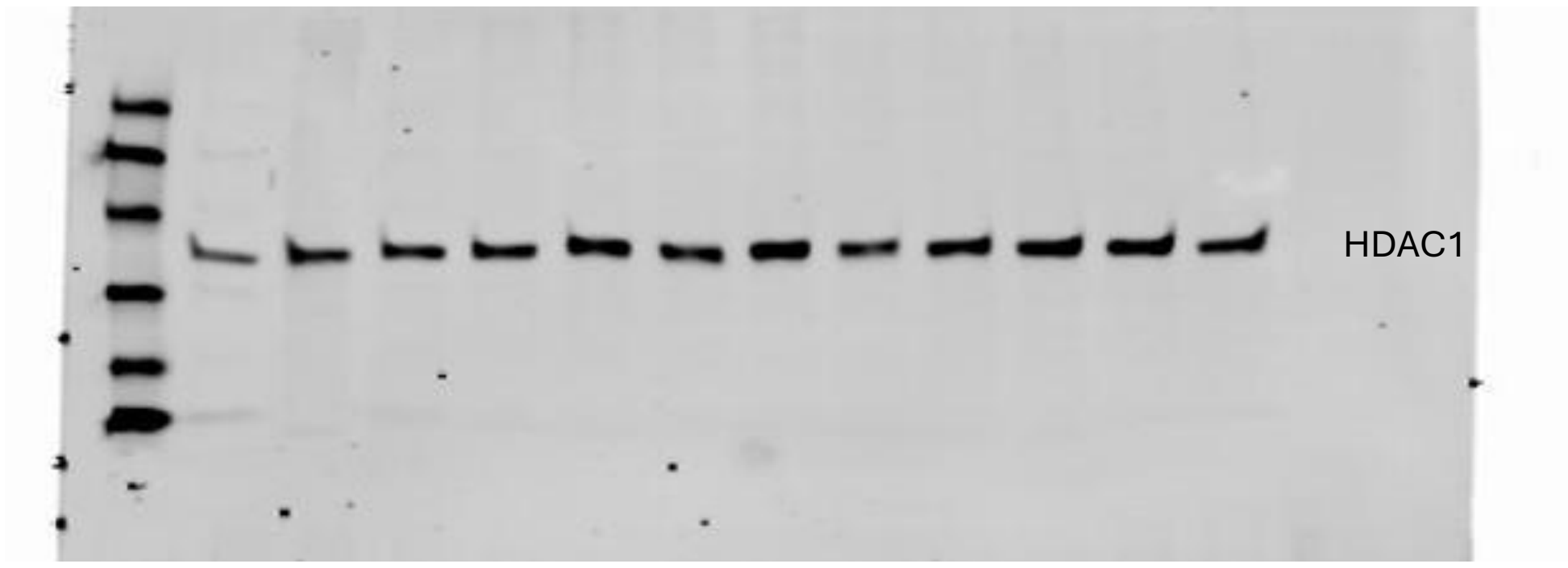

Lane #	Contents
1	Licor Chameleon Duo Ladder
2	Nuclear Enriched Lysate HEK Blue TLR1 602S – Unstimulated n1
3	Nuclear Enriched Lysate HEK Blue TLR1 602S – 30” 100 ng/mL Pam3CSK4 Stimulated n1
4	Nuclear Enriched Lysate HEK Blue TLR1 602S – 60” 100 ng/mL Pam3CSK4 Stimulated n1
5	Nuclear Enriched Lysate HEK Blue TLR1 602S – 90” 100 ng/mL Pam3CSK4 Stimulated n1
6	Nuclear Enriched Lysate HEK Blue TLR1 602S – 120” 100 ng/mL Pam3CSK4 Stimulated n1
7	Nuclear Enriched Lysate HEK Blue TLR1 602S – 180” 100 ng/mL Pam3CSK4 Stimulated n1
8	Nuclear Enriched Lysate HEK Blue TLR1 602I – Unstimulated n1
9	Nuclear Enriched Lysate HEK Blue TLR1 602I – 30” 100 ng/mL Pam3CSK4 Stimulated n1
10	Nuclear Enriched Lysate HEK Blue TLR1 602I – 60” 100 ng/mL Pam3CSK4 Stimulated n1
11	Nuclear Enriched Lysate HEK Blue TLR1 602I – 90” 100 ng/mL Pam3CSK4 Stimulated n1
12	Nuclear Enriched Lysate HEK Blue TLR1 602I – 120” 100 ng/mL Pam3CSK4 Stimulated n1
13	Nuclear Enriched Lysate HEK Blue TLR1 602I – 180” 100 ng/mL Pam3CSK4 Stimulated n1

Fig3D Representative
Channel 800

Licor Odyssey F Imager

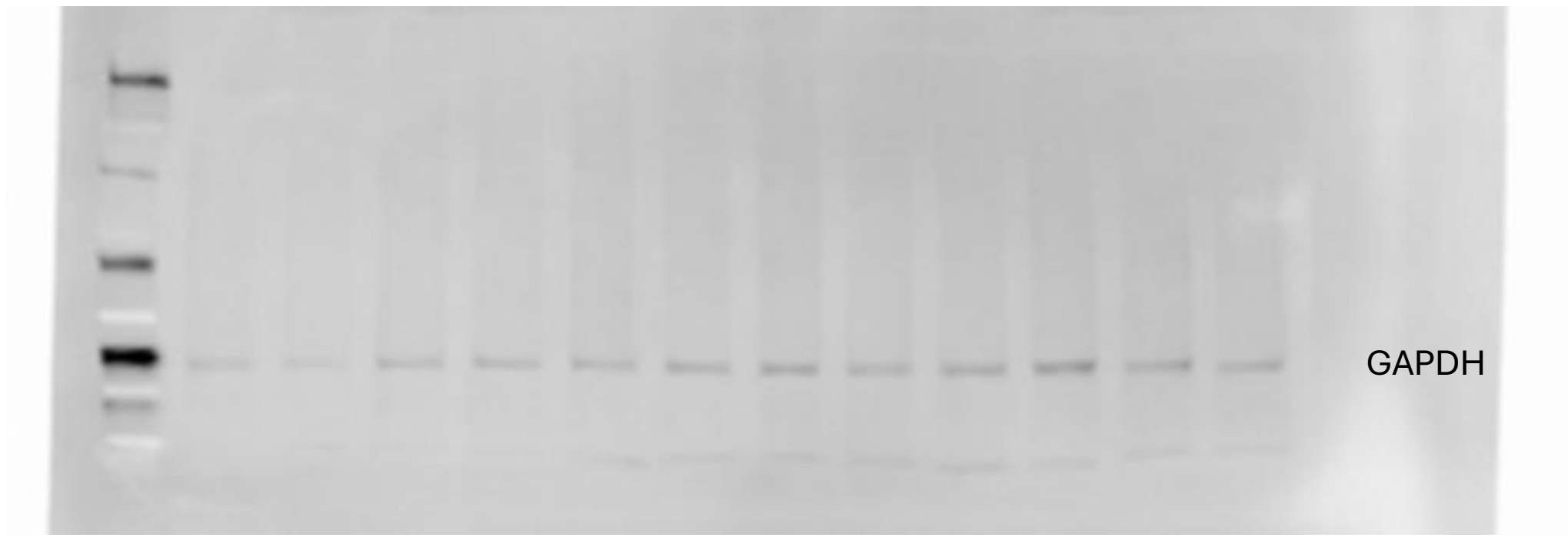

Lane #	Contents
1	Licor Chameleon Duo Ladder
2	Nuclear Enriched Lysate HEK Blue TLR1 602S – Unstimulated n1
3	Nuclear Enriched Lysate HEK Blue TLR1 602S – 30” 100 ng/mL Pam3CSK4 Stimulated n1
4	Nuclear Enriched Lysate HEK Blue TLR1 602S – 60” 100 ng/mL Pam3CSK4 Stimulated n1
5	Nuclear Enriched Lysate HEK Blue TLR1 602S – 90” 100 ng/mL Pam3CSK4 Stimulated n1
6	Nuclear Enriched Lysate HEK Blue TLR1 602S – 120” 100 ng/mL Pam3CSK4 Stimulated n1
7	Nuclear Enriched Lysate HEK Blue TLR1 602S – 180” 100 ng/mL Pam3CSK4 Stimulated n1
8	Nuclear Enriched Lysate HEK Blue TLR1 602I – Unstimulated n1
9	Nuclear Enriched Lysate HEK Blue TLR1 602I – 30” 100 ng/mL Pam3CSK4 Stimulated n1
10	Nuclear Enriched Lysate HEK Blue TLR1 602I – 60” 100 ng/mL Pam3CSK4 Stimulated n1
11	Nuclear Enriched Lysate HEK Blue TLR1 602I – 90” 100 ng/mL Pam3CSK4 Stimulated n1
12	Nuclear Enriched Lysate HEK Blue TLR1 602I – 120” 100 ng/mL Pam3CSK4 Stimulated n1
13	Nuclear Enriched Lysate HEK Blue TLR1 602I – 180” 100 ng/mL Pam3CSK4 Stimulated n1

Fig3E n2
Channel 700

Licor Odyssey F Imager

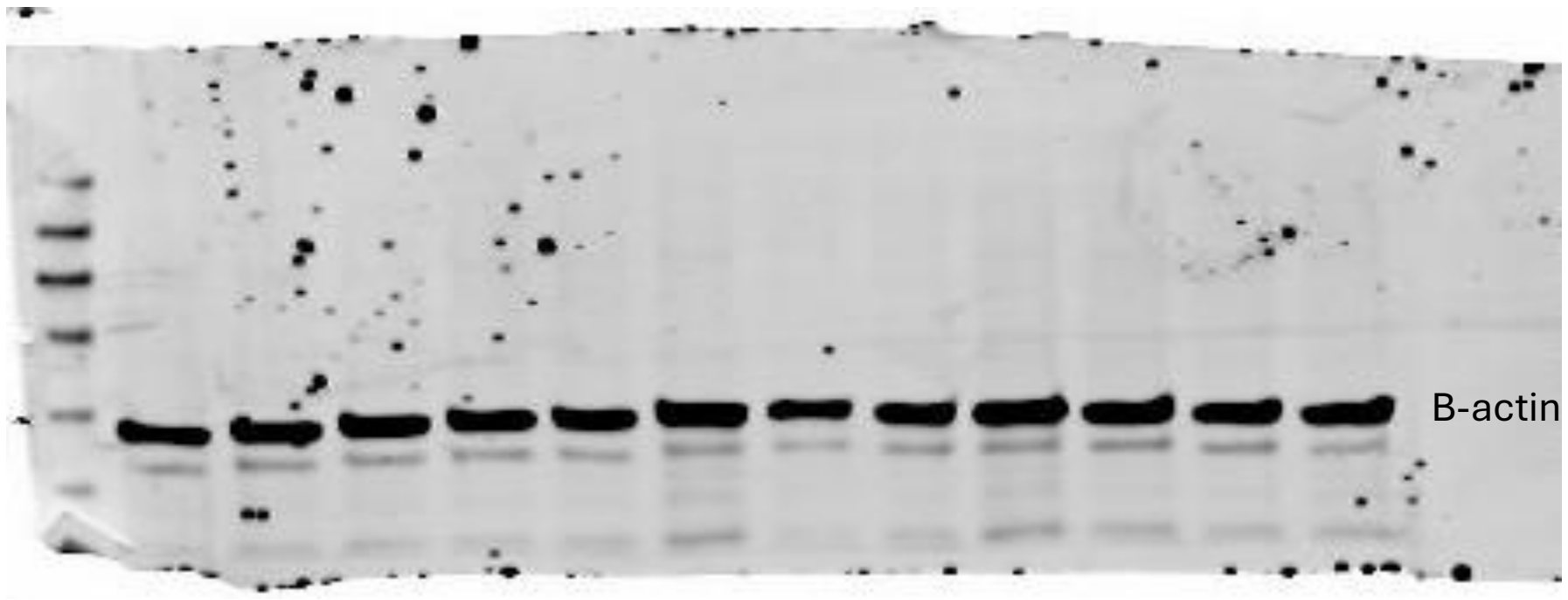

Lane #	Contents
1	Licor Chameleon Duo Ladder
2	Nuclear Enriched Lysate HEK Blue TLR1 602S – Unstimulated n2
3	Nuclear Enriched Lysate HEK Blue TLR1 602S – 30” 100 ng/mL Pam3CSK4 Stimulated n2
4	Nuclear Enriched Lysate HEK Blue TLR1 602S – 60” 100 ng/mL Pam3CSK4 Stimulated n2
5	Nuclear Enriched Lysate HEK Blue TLR1 602S – 90” 100 ng/mL Pam3CSK4 Stimulated n2
6	Nuclear Enriched Lysate HEK Blue TLR1 602S – 120” 100 ng/mL Pam3CSK4 Stimulated n2
7	Nuclear Enriched Lysate HEK Blue TLR1 602S – 180” 100 ng/mL Pam3CSK4 Stimulated n2
8	Nuclear Enriched Lysate HEK Blue TLR1 602I – Unstimulated n2
9	Nuclear Enriched Lysate HEK Blue TLR1 602I – 30” 100 ng/mL Pam3CSK4 Stimulated n2
10	Nuclear Enriched Lysate HEK Blue TLR1 602I – 60” 100 ng/mL Pam3CSK4 Stimulated n2
11	Nuclear Enriched Lysate HEK Blue TLR1 602I – 90” 100 ng/mL Pam3CSK4 Stimulated n2
12	Nuclear Enriched Lysate HEK Blue TLR1 602I – 120” 100 ng/mL Pam3CSK4 Stimulated n2
13	Nuclear Enriched Lysate HEK Blue TLR1 602I – 180” 100 ng/mL Pam3CSK4 Stimulated n2

Fig3E n2
Channel 800

Licor Odyssey F Imager

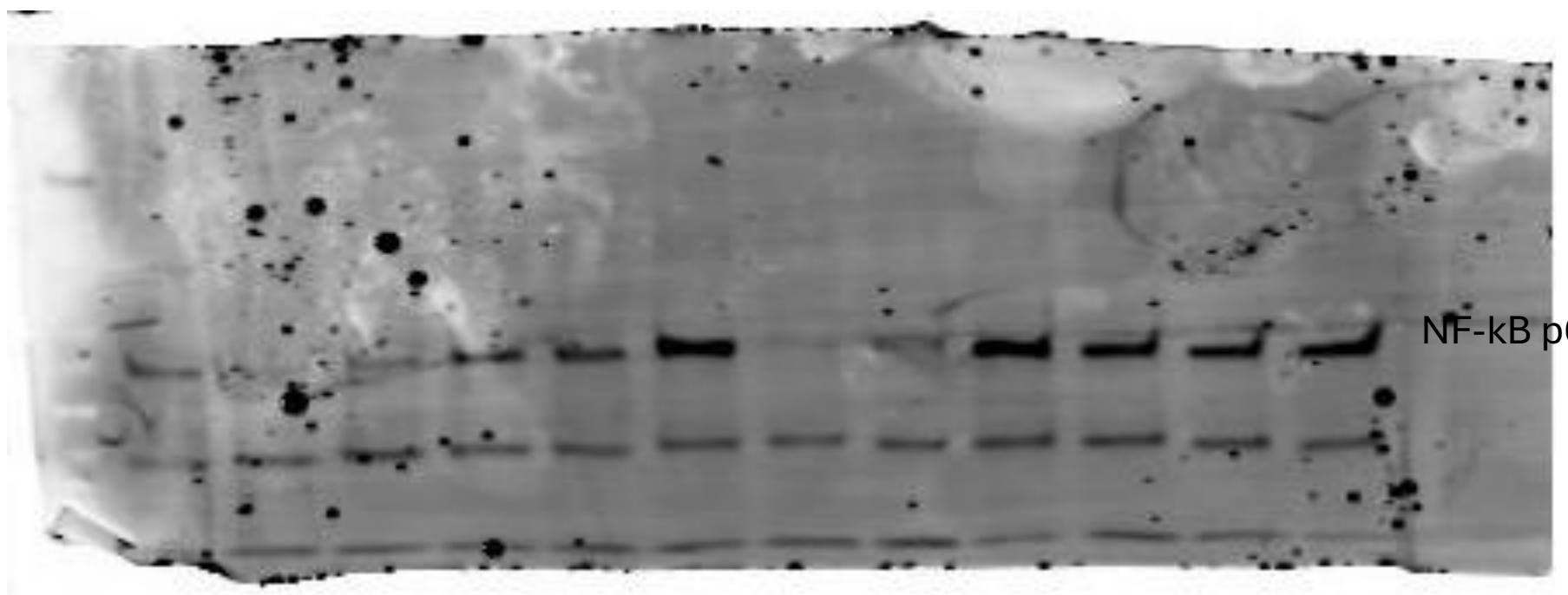

Lane #	Contents
1	Licor Chameleon Duo Ladder
2	Nuclear Enriched Lysate HEK Blue TLR1 602S – Unstimulated n2
3	Nuclear Enriched Lysate HEK Blue TLR1 602S – 30” 100 ng/mL Pam3CSK4 Stimulated n2
4	Nuclear Enriched Lysate HEK Blue TLR1 602S – 60” 100 ng/mL Pam3CSK4 Stimulated n2
5	Nuclear Enriched Lysate HEK Blue TLR1 602S – 90” 100 ng/mL Pam3CSK4 Stimulated n2
6	Nuclear Enriched Lysate HEK Blue TLR1 602S – 120” 100 ng/mL Pam3CSK4 Stimulated n2
7	Nuclear Enriched Lysate HEK Blue TLR1 602S – 180” 100 ng/mL Pam3CSK4 Stimulated n2
8	Nuclear Enriched Lysate HEK Blue TLR1 602I – Unstimulated n2
9	Nuclear Enriched Lysate HEK Blue TLR1 602I – 30” 100 ng/mL Pam3CSK4 Stimulated n2
10	Nuclear Enriched Lysate HEK Blue TLR1 602I – 60” 100 ng/mL Pam3CSK4 Stimulated n2
11	Nuclear Enriched Lysate HEK Blue TLR1 602I – 90” 100 ng/mL Pam3CSK4 Stimulated n2
12	Nuclear Enriched Lysate HEK Blue TLR1 602I – 120” 100 ng/mL Pam3CSK4 Stimulated n2
13	Nuclear Enriched Lysate HEK Blue TLR1 602I – 180” 100 ng/mL Pam3CSK4 Stimulated n2

Fig3E n3
Channel 700

Licor Odyssey F Imager

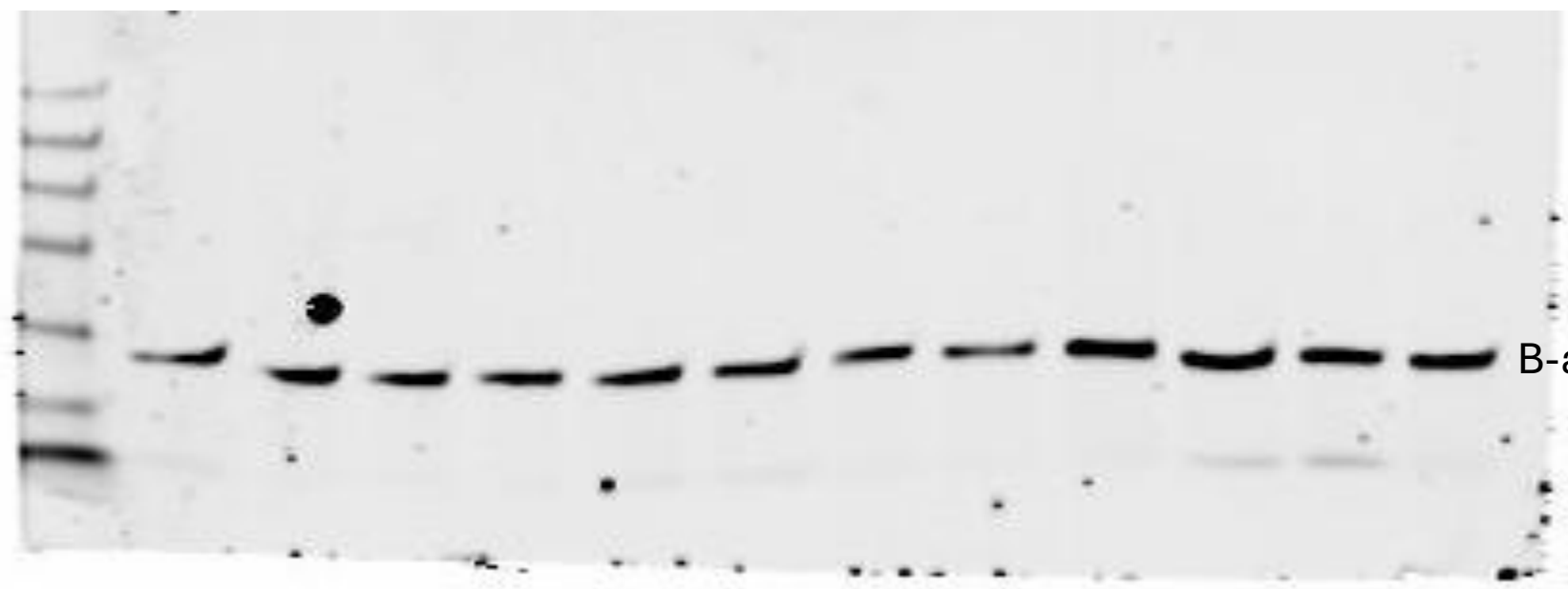

Lane #	Contents
1	Licor Chameleon Duo Ladder
2	Nuclear Enriched Lysate HEK Blue TLR1 602S – Unstimulated n3
3	Nuclear Enriched Lysate HEK Blue TLR1 602S – 30” 100 ng/mL Pam3CSK4 Stimulated n3
4	Nuclear Enriched Lysate HEK Blue TLR1 602S – 60” 100 ng/mL Pam3CSK4 Stimulated n3
5	Nuclear Enriched Lysate HEK Blue TLR1 602S – 90” 100 ng/mL Pam3CSK4 Stimulated n3
6	Nuclear Enriched Lysate HEK Blue TLR1 602S – 120” 100 ng/mL Pam3CSK4 Stimulated n3
7	Nuclear Enriched Lysate HEK Blue TLR1 602S – 180” 100 ng/mL Pam3CSK4 Stimulated n3
8	Nuclear Enriched Lysate HEK Blue TLR1 602I – Unstimulated n3
9	Nuclear Enriched Lysate HEK Blue TLR1 602I – 30” 100 ng/mL Pam3CSK4 Stimulated n3
10	Nuclear Enriched Lysate HEK Blue TLR1 602I – 60” 100 ng/mL Pam3CSK4 Stimulated n3
11	Nuclear Enriched Lysate HEK Blue TLR1 602I – 90” 100 ng/mL Pam3CSK4 Stimulated n3
12	Nuclear Enriched Lysate HEK Blue TLR1 602I – 120” 100 ng/mL Pam3CSK4 Stimulated n3
13	Nuclear Enriched Lysate HEK Blue TLR1 602I – 180” 100 ng/mL Pam3CSK4 Stimulated n3

Fig3E n3
Channel 800

Licor Odyssey F Imager

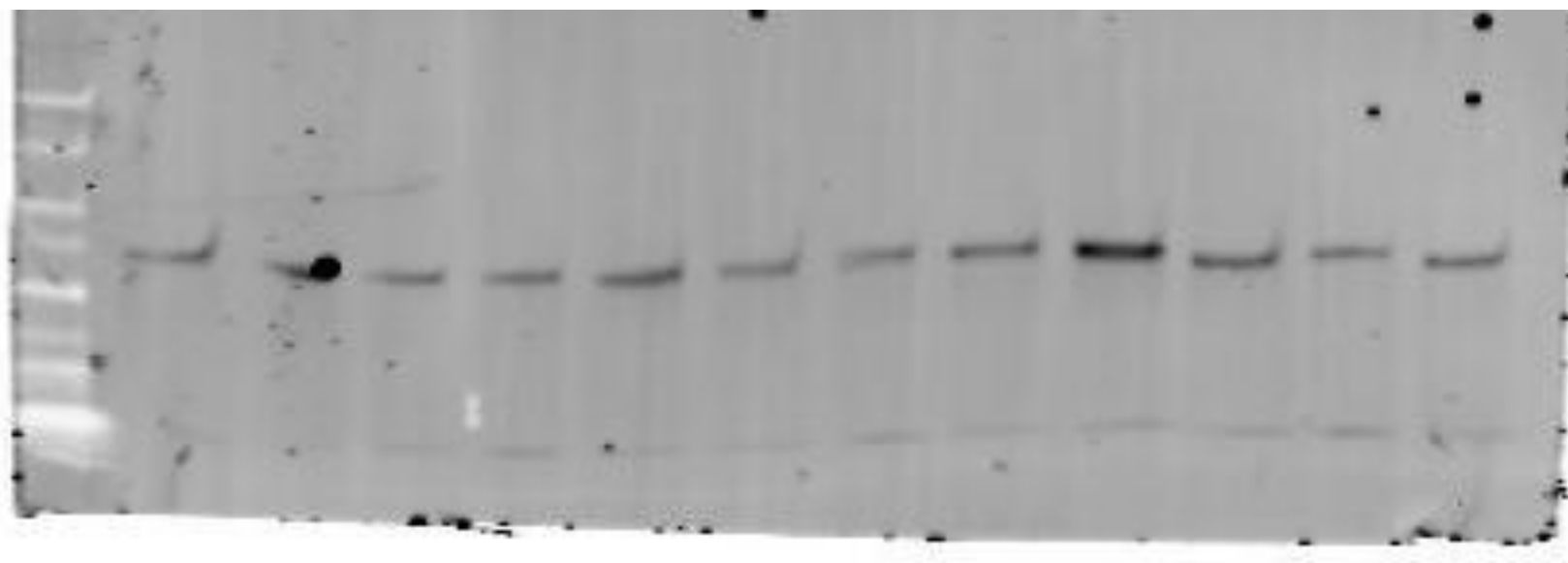

Lane #	Contents
1	Licor Chameleon Duo Ladder
2	Nuclear Enriched Lysate HEK Blue TLR1 602S – Unstimulated n3
3	Nuclear Enriched Lysate HEK Blue TLR1 602S – 30” 100 ng/mL Pam3CSK4 Stimulated n3
4	Nuclear Enriched Lysate HEK Blue TLR1 602S – 60” 100 ng/mL Pam3CSK4 Stimulated n3
5	Nuclear Enriched Lysate HEK Blue TLR1 602S – 90” 100 ng/mL Pam3CSK4 Stimulated n3
6	Nuclear Enriched Lysate HEK Blue TLR1 602S – 120” 100 ng/mL Pam3CSK4 Stimulated n3
7	Nuclear Enriched Lysate HEK Blue TLR1 602S – 180” 100 ng/mL Pam3CSK4 Stimulated n3
8	Nuclear Enriched Lysate HEK Blue TLR1 602I – Unstimulated n3
9	Nuclear Enriched Lysate HEK Blue TLR1 602I – 30” 100 ng/mL Pam3CSK4 Stimulated n3
10	Nuclear Enriched Lysate HEK Blue TLR1 602I – 60” 100 ng/mL Pam3CSK4 Stimulated n3
11	Nuclear Enriched Lysate HEK Blue TLR1 602I – 90” 100 ng/mL Pam3CSK4 Stimulated n3
12	Nuclear Enriched Lysate HEK Blue TLR1 602I – 120” 100 ng/mL Pam3CSK4 Stimulated n3
13	Nuclear Enriched Lysate HEK Blue TLR1 602I – 180” 100 ng/mL Pam3CSK4 Stimulated n3

Fig4A Representative and Fig4B n1
Channel 700

Licor Odyssey F Imager

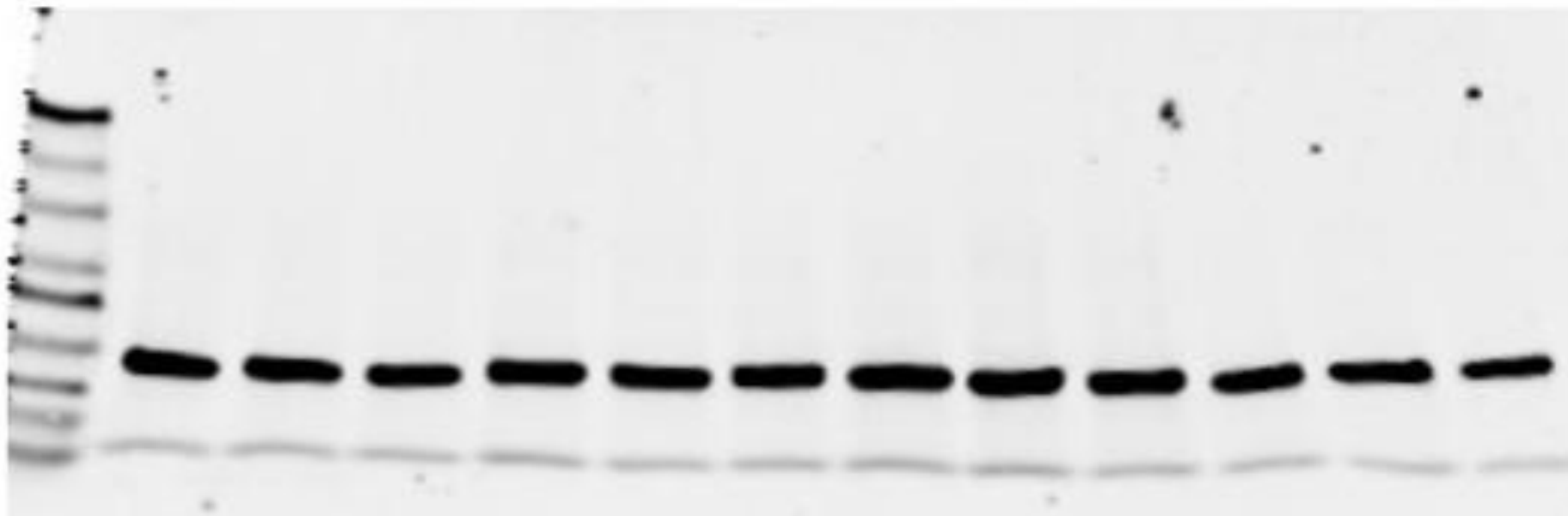

Lane #	Contents
1	Licor Chameleon Duo Ladder
2	Nuclear Enriched Lysate HEK Blue TLR1 602S – Unstimulated n1
3	Nuclear Enriched Lysate HEK Blue TLR1 602S – 30” 100 ng/mL Pam3CSK4 Stimulated n1
4	Nuclear Enriched Lysate HEK Blue TLR1 602S – 60” 100 ng/mL Pam3CSK4 Stimulated n1
5	Nuclear Enriched Lysate HEK Blue TLR1 602S – 90” 100 ng/mL Pam3CSK4 Stimulated n1
6	Nuclear Enriched Lysate HEK Blue TLR1 602S – 120” 100 ng/mL Pam3CSK4 Stimulated n1
7	Nuclear Enriched Lysate HEK Blue TLR1 602S – 180” 100 ng/mL Pam3CSK4 Stimulated n1
8	Nuclear Enriched Lysate HEK Blue TLR1 602I – Unstimulated n1
9	Nuclear Enriched Lysate HEK Blue TLR1 602I – 30” 100 ng/mL Pam3CSK4 Stimulated n1
10	Nuclear Enriched Lysate HEK Blue TLR1 602I – 60” 100 ng/mL Pam3CSK4 Stimulated n1
11	Nuclear Enriched Lysate HEK Blue TLR1 602I – 90” 100 ng/mL Pam3CSK4 Stimulated n1
12	Nuclear Enriched Lysate HEK Blue TLR1 602I – 120” 100 ng/mL Pam3CSK4 Stimulated n1
13	Nuclear Enriched Lysate HEK Blue TLR1 602I – 180” 100 ng/mL Pam3CSK4 Stimulated n1

Fig4A Representative and Fig4B n1
Channel 800

Licor Odyssey F Imager

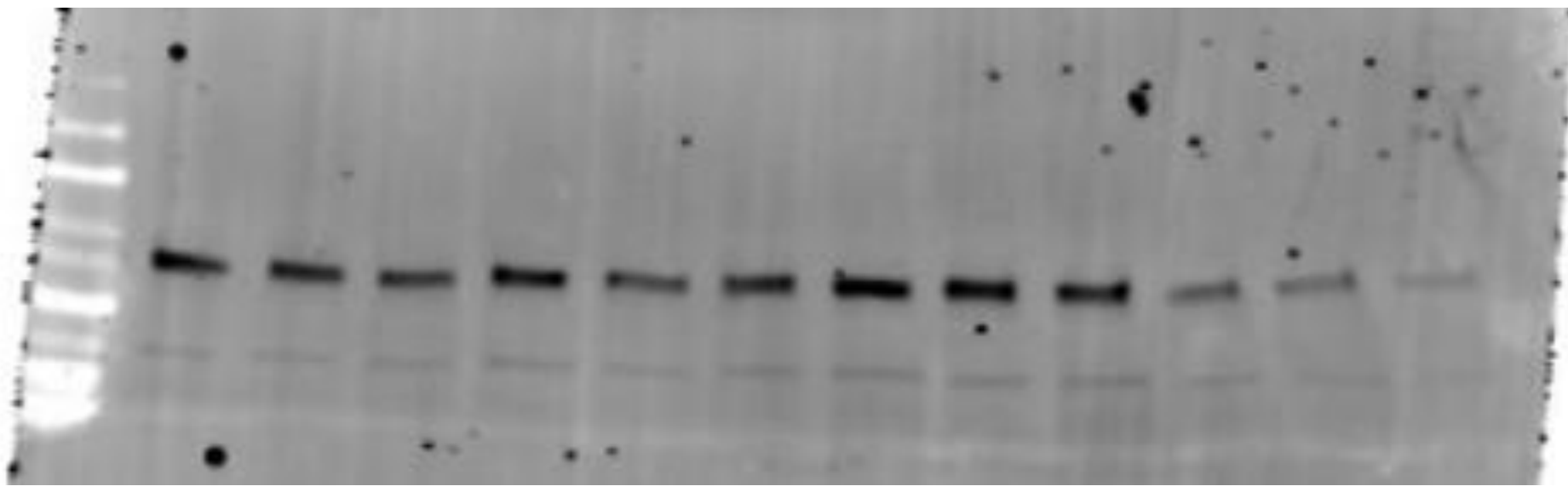

Lane #	Contents
1	Licor Chameleon Duo Ladder
2	Nuclear Enriched Lysate HEK Blue TLR1 602S – Unstimulated n1
3	Nuclear Enriched Lysate HEK Blue TLR1 602S – 30” 100 ng/mL Pam3CSK4 Stimulated n1
4	Nuclear Enriched Lysate HEK Blue TLR1 602S – 60” 100 ng/mL Pam3CSK4 Stimulated n1
5	Nuclear Enriched Lysate HEK Blue TLR1 602S – 90” 100 ng/mL Pam3CSK4 Stimulated n1
6	Nuclear Enriched Lysate HEK Blue TLR1 602S – 120” 100 ng/mL Pam3CSK4 Stimulated n1
7	Nuclear Enriched Lysate HEK Blue TLR1 602S – 180” 100 ng/mL Pam3CSK4 Stimulated n1
8	Nuclear Enriched Lysate HEK Blue TLR1 602I – Unstimulated n1
9	Nuclear Enriched Lysate HEK Blue TLR1 602I – 30” 100 ng/mL Pam3CSK4 Stimulated n1
10	Nuclear Enriched Lysate HEK Blue TLR1 602I – 60” 100 ng/mL Pam3CSK4 Stimulated n1
11	Nuclear Enriched Lysate HEK Blue TLR1 602I – 90” 100 ng/mL Pam3CSK4 Stimulated n1
12	Nuclear Enriched Lysate HEK Blue TLR1 602I – 120” 100 ng/mL Pam3CSK4 Stimulated n1
13	Nuclear Enriched Lysate HEK Blue TLR1 602I – 180” 100 ng/mL Pam3CSK4 Stimulated n1

Fig4A Representative Channel 700

Licor Odyssey F Imager

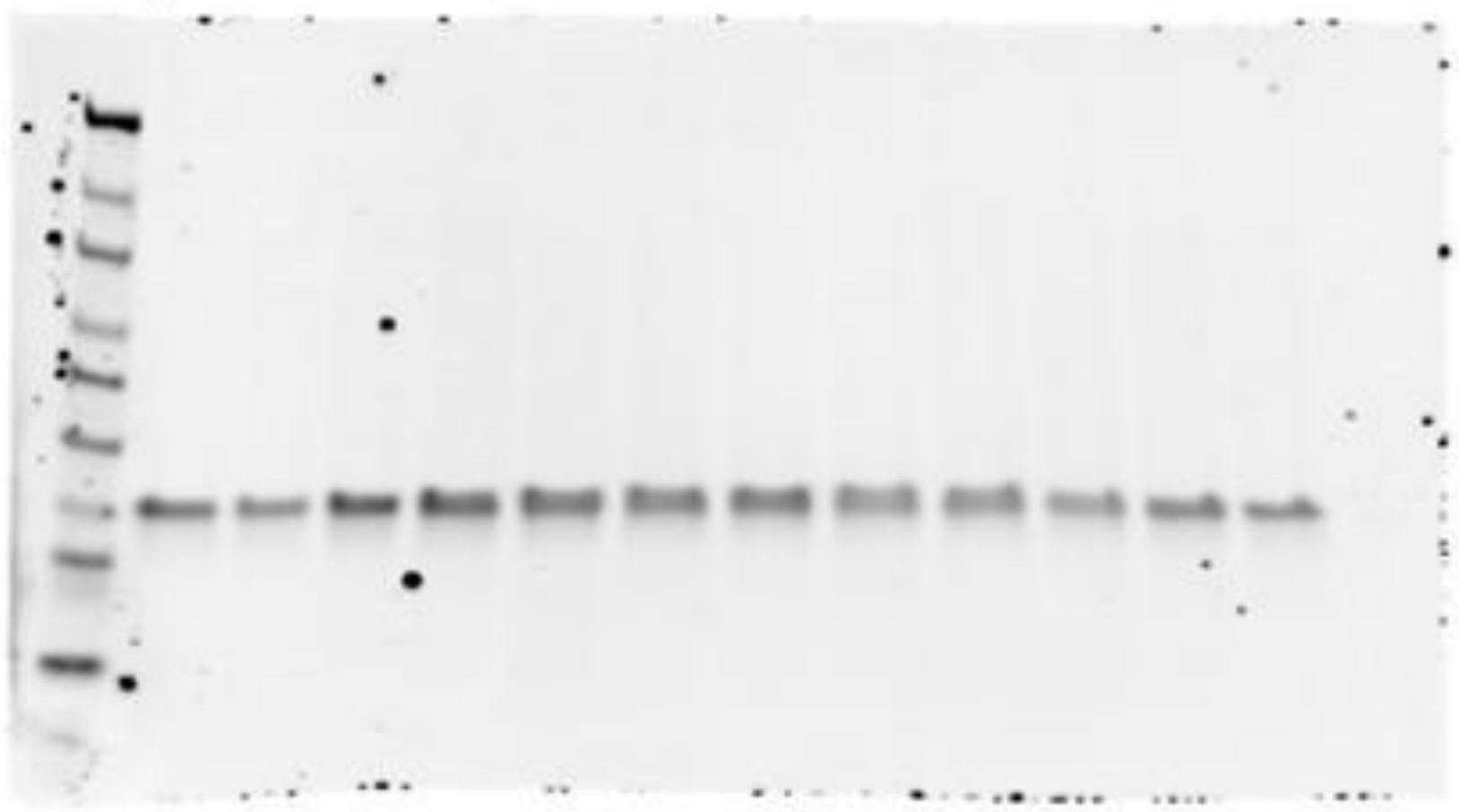

Lane #	Contents
1	Licor Chameleon Duo Ladder
2	Nuclear Enriched Lysate HEK Blue TLR1 602S – Unstimulated n1
3	Nuclear Enriched Lysate HEK Blue TLR1 602S – 30” 100 ng/mL Pam3CSK4 Stimulated n1
4	Nuclear Enriched Lysate HEK Blue TLR1 602S – 60” 100 ng/mL Pam3CSK4 Stimulated n1
5	Nuclear Enriched Lysate HEK Blue TLR1 602S – 90” 100 ng/mL Pam3CSK4 Stimulated n1
6	Nuclear Enriched Lysate HEK Blue TLR1 602S – 120” 100 ng/mL Pam3CSK4 Stimulated n1
7	Nuclear Enriched Lysate HEK Blue TLR1 602S – 180” 100 ng/mL Pam3CSK4 Stimulated n1
8	Nuclear Enriched Lysate HEK Blue TLR1 602I – Unstimulated n1
9	Nuclear Enriched Lysate HEK Blue TLR1 602I – 30” 100 ng/mL Pam3CSK4 Stimulated n1
10	Nuclear Enriched Lysate HEK Blue TLR1 602I – 60” 100 ng/mL Pam3CSK4 Stimulated n1
11	Nuclear Enriched Lysate HEK Blue TLR1 602I – 90” 100 ng/mL Pam3CSK4 Stimulated n1
12	Nuclear Enriched Lysate HEK Blue TLR1 602I – 120” 100 ng/mL Pam3CSK4 Stimulated n1
13	Nuclear Enriched Lysate HEK Blue TLR1 602I – 180” 100 ng/mL Pam3CSK4 Stimulated n1

Fig4A Representative Channel 800

Licor Odyssey F Imager

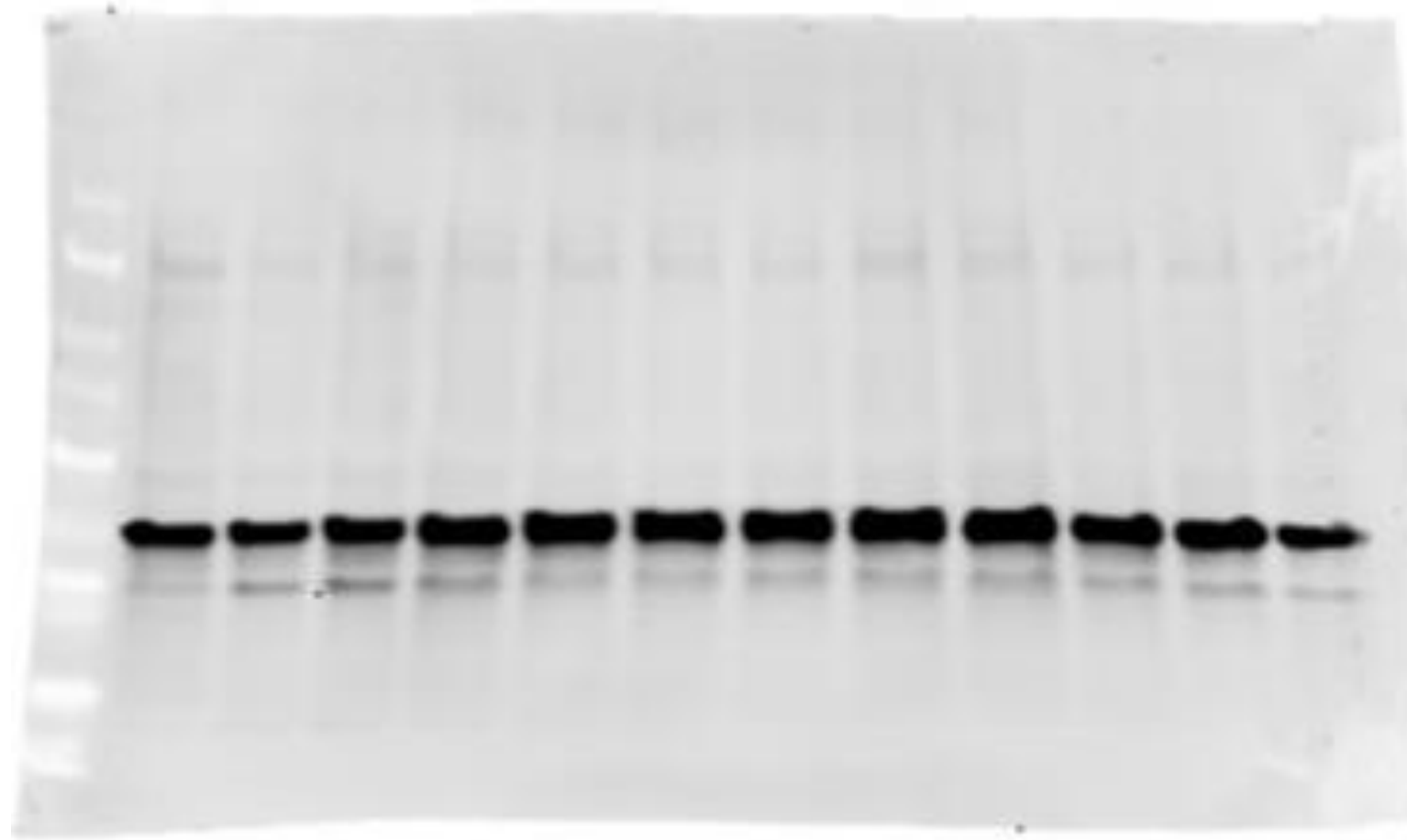

Lane #	Contents
1	Licor Chameleon Duo Ladder
2	Nuclear Enriched Lysate HEK Blue TLR1 602S – Unstimulated n1
3	Nuclear Enriched Lysate HEK Blue TLR1 602S – 30” 100 ng/mL Pam3CSK4 Stimulated n1
4	Nuclear Enriched Lysate HEK Blue TLR1 602S – 60” 100 ng/mL Pam3CSK4 Stimulated n1
5	Nuclear Enriched Lysate HEK Blue TLR1 602S – 90” 100 ng/mL Pam3CSK4 Stimulated n1
6	Nuclear Enriched Lysate HEK Blue TLR1 602S – 120” 100 ng/mL Pam3CSK4 Stimulated n1
7	Nuclear Enriched Lysate HEK Blue TLR1 602S – 180” 100 ng/mL Pam3CSK4 Stimulated n1
8	Nuclear Enriched Lysate HEK Blue TLR1 602I – Unstimulated n1
9	Nuclear Enriched Lysate HEK Blue TLR1 602I – 30” 100 ng/mL Pam3CSK4 Stimulated n1
10	Nuclear Enriched Lysate HEK Blue TLR1 602I – 60” 100 ng/mL Pam3CSK4 Stimulated n1
11	Nuclear Enriched Lysate HEK Blue TLR1 602I – 90” 100 ng/mL Pam3CSK4 Stimulated n1
12	Nuclear Enriched Lysate HEK Blue TLR1 602I – 120” 100 ng/mL Pam3CSK4 Stimulated n1
13	Nuclear Enriched Lysate HEK Blue TLR1 602I – 180” 100 ng/mL Pam3CSK4 Stimulated n1

Fig4B n2
Channel 700

Licor Odyssey F Imager

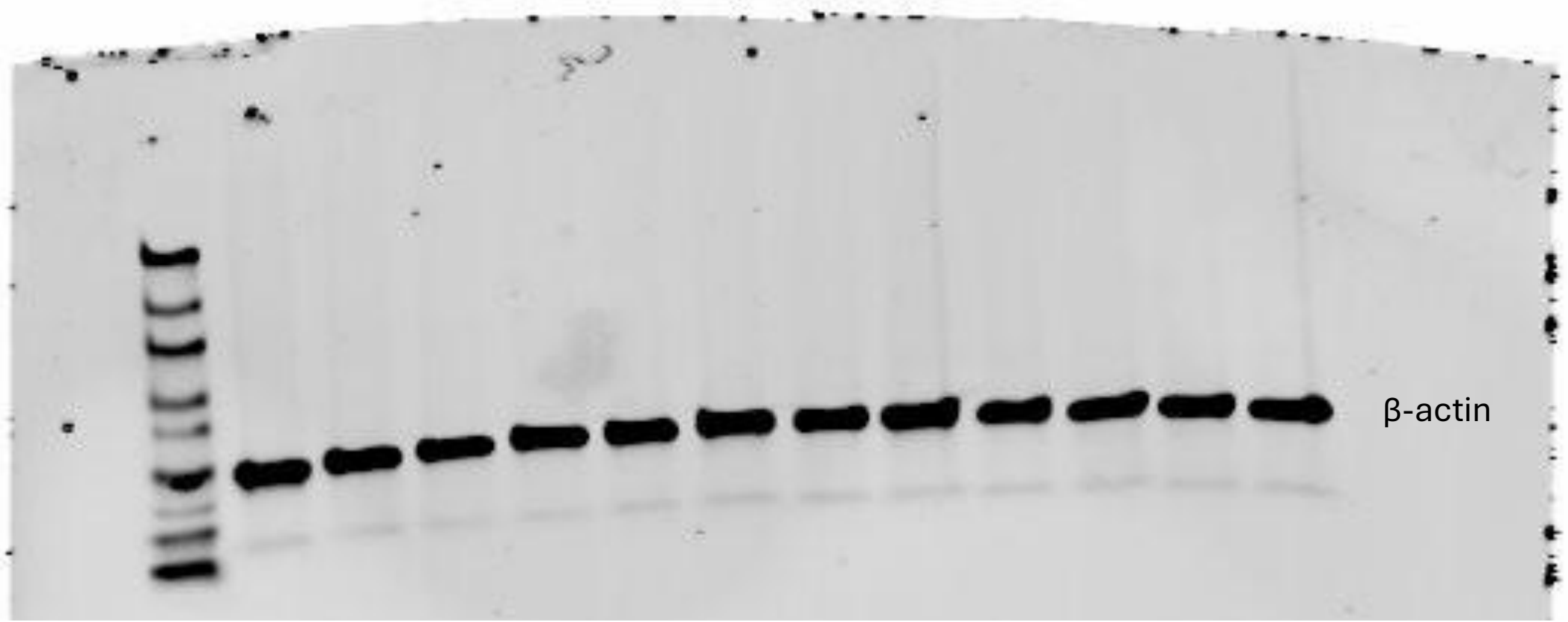

Lane #	Contents
1	Licor Chameleon Duo Ladder
2	Nuclear Enriched Lysate HEK Blue TLR1 602S – Unstimulated n2
3	Nuclear Enriched Lysate HEK Blue TLR1 602S – 30” 100 ng/mL Pam3CSK4 Stimulated n2
4	Nuclear Enriched Lysate HEK Blue TLR1 602S – 60” 100 ng/mL Pam3CSK4 Stimulated n2
5	Nuclear Enriched Lysate HEK Blue TLR1 602S – 90” 100 ng/mL Pam3CSK4 Stimulated n2
6	Nuclear Enriched Lysate HEK Blue TLR1 602S – 120” 100 ng/mL Pam3CSK4 Stimulated n2
7	Nuclear Enriched Lysate HEK Blue TLR1 602S – 180” 100 ng/mL Pam3CSK4 Stimulated n2
8	Nuclear Enriched Lysate HEK Blue TLR1 602I – Unstimulated n2
9	Nuclear Enriched Lysate HEK Blue TLR1 602I – 30” 100 ng/mL Pam3CSK4 Stimulated n2
10	Nuclear Enriched Lysate HEK Blue TLR1 602I – 60” 100 ng/mL Pam3CSK4 Stimulated n2
11	Nuclear Enriched Lysate HEK Blue TLR1 602I – 90” 100 ng/mL Pam3CSK4 Stimulated n2
12	Nuclear Enriched Lysate HEK Blue TLR1 602I – 120” 100 ng/mL Pam3CSK4 Stimulated n2
13	Nuclear Enriched Lysate HEK Blue TLR1 602I – 180” 100 ng/mL Pam3CSK4 Stimulated n2

Fig4B n2
Channel 800

Licor Odyssey F Imager

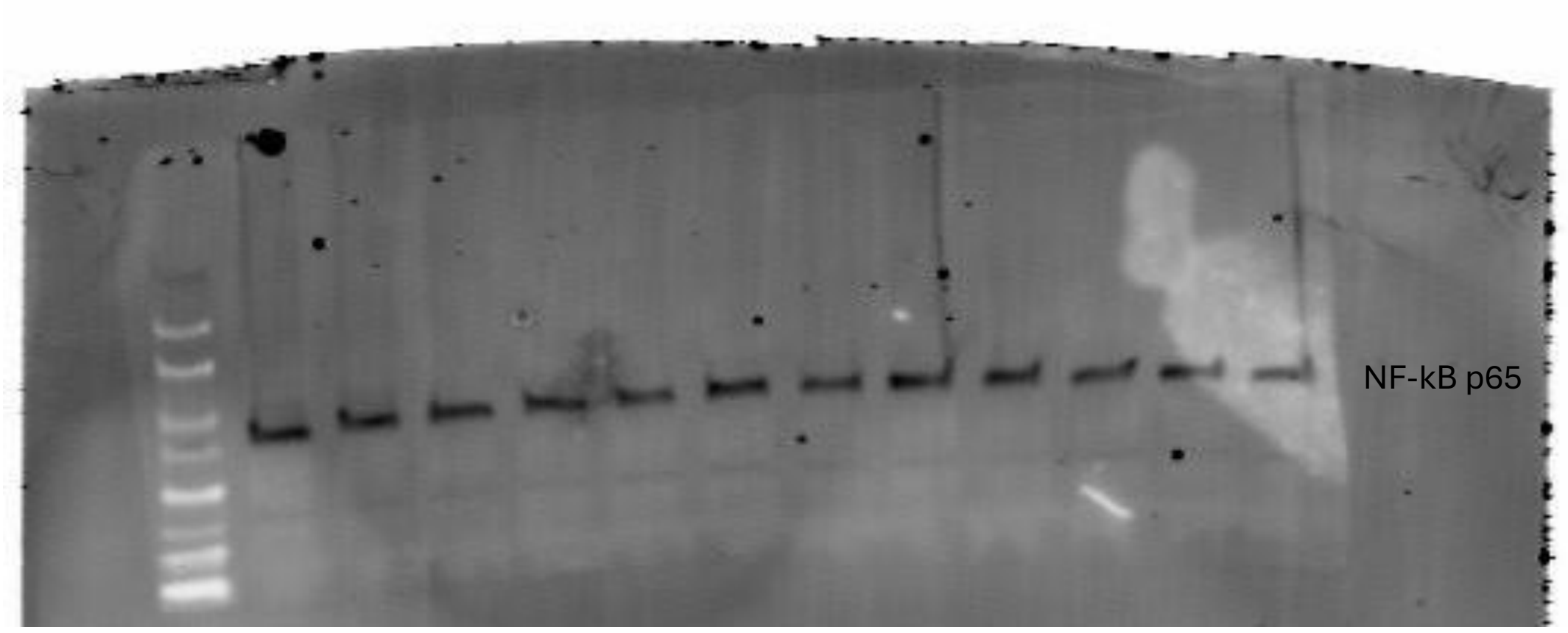

Lane #	Contents
1	Licor Chameleon Duo Ladder
2	Nuclear Enriched Lysate HEK Blue TLR1 602S – Unstimulated n2
3	Nuclear Enriched Lysate HEK Blue TLR1 602S – 30” 100 ng/mL Pam3CSK4 Stimulated n2
4	Nuclear Enriched Lysate HEK Blue TLR1 602S – 60” 100 ng/mL Pam3CSK4 Stimulated n2
5	Nuclear Enriched Lysate HEK Blue TLR1 602S – 90” 100 ng/mL Pam3CSK4 Stimulated n2
6	Nuclear Enriched Lysate HEK Blue TLR1 602S – 120” 100 ng/mL Pam3CSK4 Stimulated n2
7	Nuclear Enriched Lysate HEK Blue TLR1 602S – 180” 100 ng/mL Pam3CSK4 Stimulated n2
8	Nuclear Enriched Lysate HEK Blue TLR1 602I – Unstimulated n2
9	Nuclear Enriched Lysate HEK Blue TLR1 602I – 30” 100 ng/mL Pam3CSK4 Stimulated n2
10	Nuclear Enriched Lysate HEK Blue TLR1 602I – 60” 100 ng/mL Pam3CSK4 Stimulated n2
11	Nuclear Enriched Lysate HEK Blue TLR1 602I – 90” 100 ng/mL Pam3CSK4 Stimulated n2
12	Nuclear Enriched Lysate HEK Blue TLR1 602I – 120” 100 ng/mL Pam3CSK4 Stimulated n2
13	Nuclear Enriched Lysate HEK Blue TLR1 602I – 180” 100 ng/mL Pam3CSK4 Stimulated n2

Fig4B n3

Channel 700

Licor Odyssey F Imager

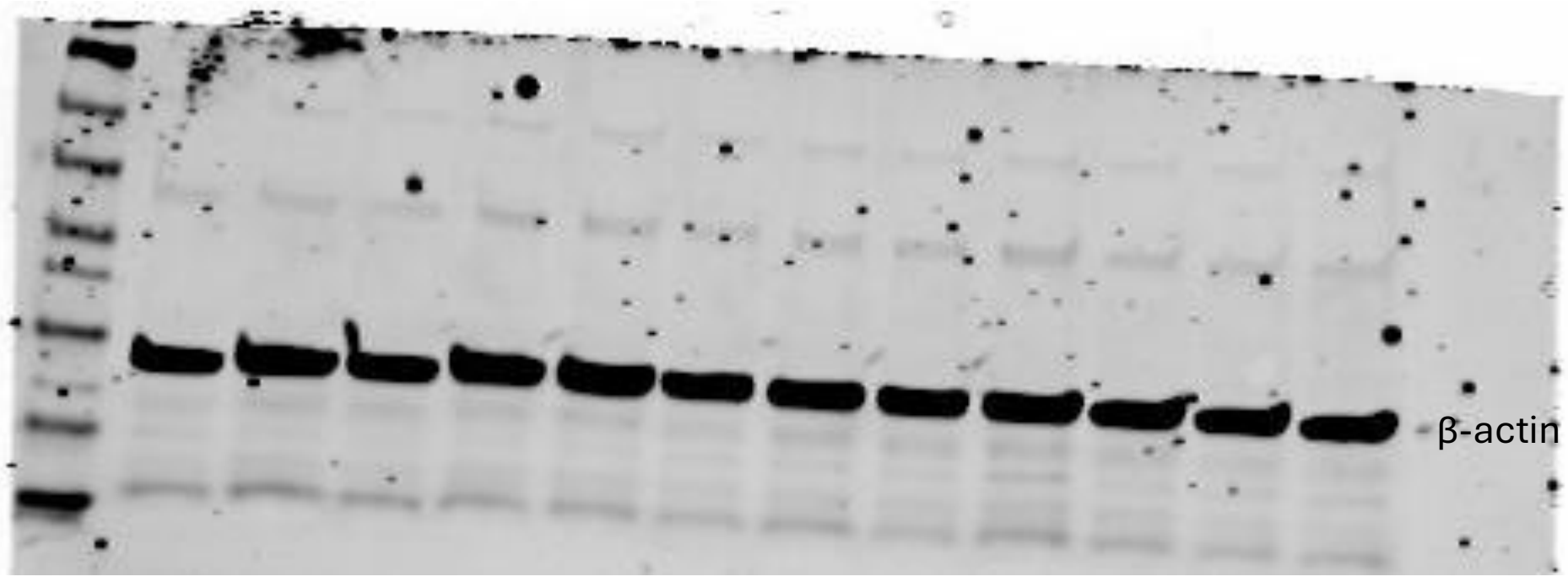

Lane #	Contents
1	Licor Chameleon Duo Ladder
2	Nuclear Enriched Lysate HEK Blue TLR1 602S – Unstimulated n3
3	Nuclear Enriched Lysate HEK Blue TLR1 602S – 30” 100 ng/mL Pam3CSK4 Stimulated n3
4	Nuclear Enriched Lysate HEK Blue TLR1 602S – 60” 100 ng/mL Pam3CSK4 Stimulated n3
5	Nuclear Enriched Lysate HEK Blue TLR1 602S – 90” 100 ng/mL Pam3CSK4 Stimulated n3
6	Nuclear Enriched Lysate HEK Blue TLR1 602S – 120” 100 ng/mL Pam3CSK4 Stimulated n3
7	Nuclear Enriched Lysate HEK Blue TLR1 602S – 180” 100 ng/mL Pam3CSK4 Stimulated n3
8	Nuclear Enriched Lysate HEK Blue TLR1 602I – Unstimulated n3
9	Nuclear Enriched Lysate HEK Blue TLR1 602I – 30” 100 ng/mL Pam3CSK4 Stimulated n3
10	Nuclear Enriched Lysate HEK Blue TLR1 602I – 60” 100 ng/mL Pam3CSK4 Stimulated n3
11	Nuclear Enriched Lysate HEK Blue TLR1 602I – 90” 100 ng/mL Pam3CSK4 Stimulated n3
12	Nuclear Enriched Lysate HEK Blue TLR1 602I – 120” 100 ng/mL Pam3CSK4 Stimulated n3
13	Nuclear Enriched Lysate HEK Blue TLR1 602I – 180” 100 ng/mL Pam3CSK4 Stimulated n3

Fig4B n3
Channel 800

Licor Odyssey F Imager

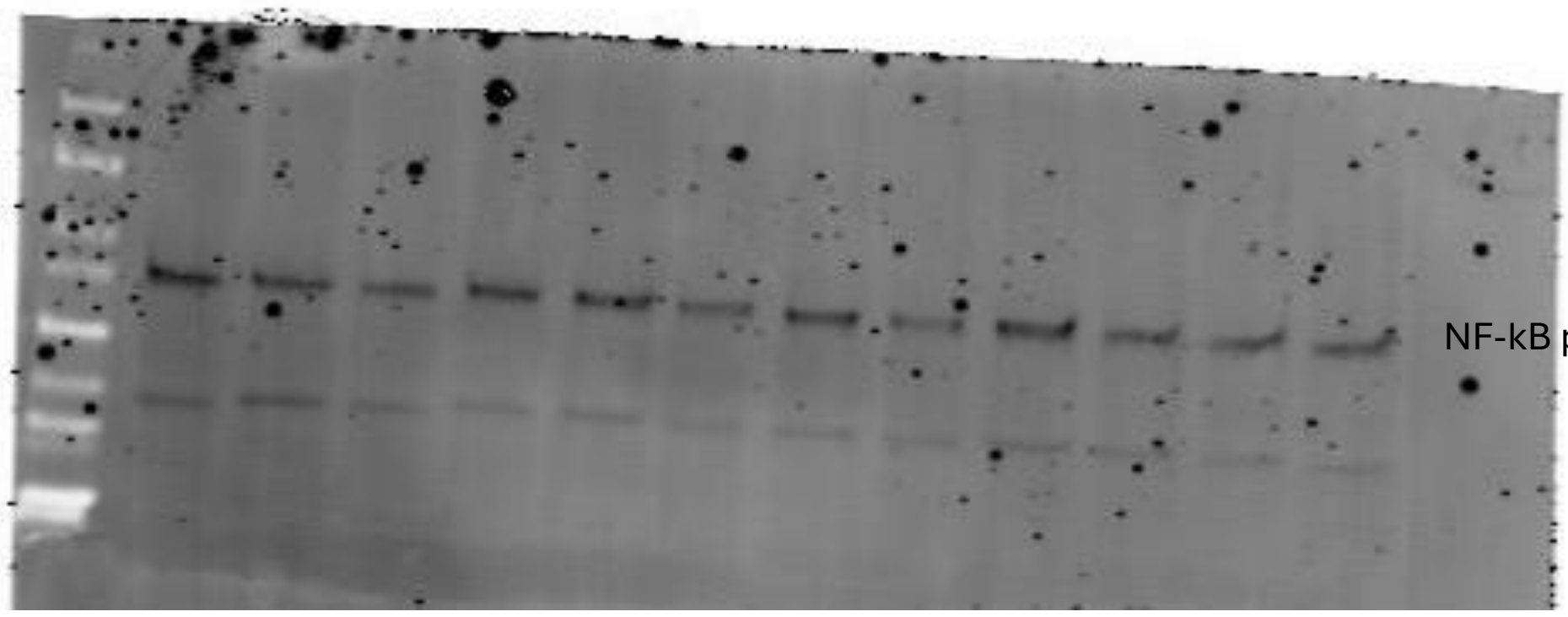

Lane #	Contents
1	Licor Chameleon Duo Ladder
2	Nuclear Enriched Lysate HEK Blue TLR1 602S – Unstimulated n3
3	Nuclear Enriched Lysate HEK Blue TLR1 602S – 30” 100 ng/mL Pam3CSK4 Stimulated n3
4	Nuclear Enriched Lysate HEK Blue TLR1 602S – 60” 100 ng/mL Pam3CSK4 Stimulated n3
5	Nuclear Enriched Lysate HEK Blue TLR1 602S – 90” 100 ng/mL Pam3CSK4 Stimulated n3
6	Nuclear Enriched Lysate HEK Blue TLR1 602S – 120” 100 ng/mL Pam3CSK4 Stimulated n3
7	Nuclear Enriched Lysate HEK Blue TLR1 602S – 180” 100 ng/mL Pam3CSK4 Stimulated n3
8	Nuclear Enriched Lysate HEK Blue TLR1 602I – Unstimulated n3
9	Nuclear Enriched Lysate HEK Blue TLR1 602I – 30” 100 ng/mL Pam3CSK4 Stimulated n3
10	Nuclear Enriched Lysate HEK Blue TLR1 602I – 60” 100 ng/mL Pam3CSK4 Stimulated n3
11	Nuclear Enriched Lysate HEK Blue TLR1 602I – 90” 100 ng/mL Pam3CSK4 Stimulated n3
12	Nuclear Enriched Lysate HEK Blue TLR1 602I – 120” 100 ng/mL Pam3CSK4 Stimulated n3
13	Nuclear Enriched Lysate HEK Blue TLR1 602I – 180” 100 ng/mL Pam3CSK4 Stimulated n3

Fig4C n2 (top) and n3 (bottom)
Channel 700

Licor Odyssey F Imager

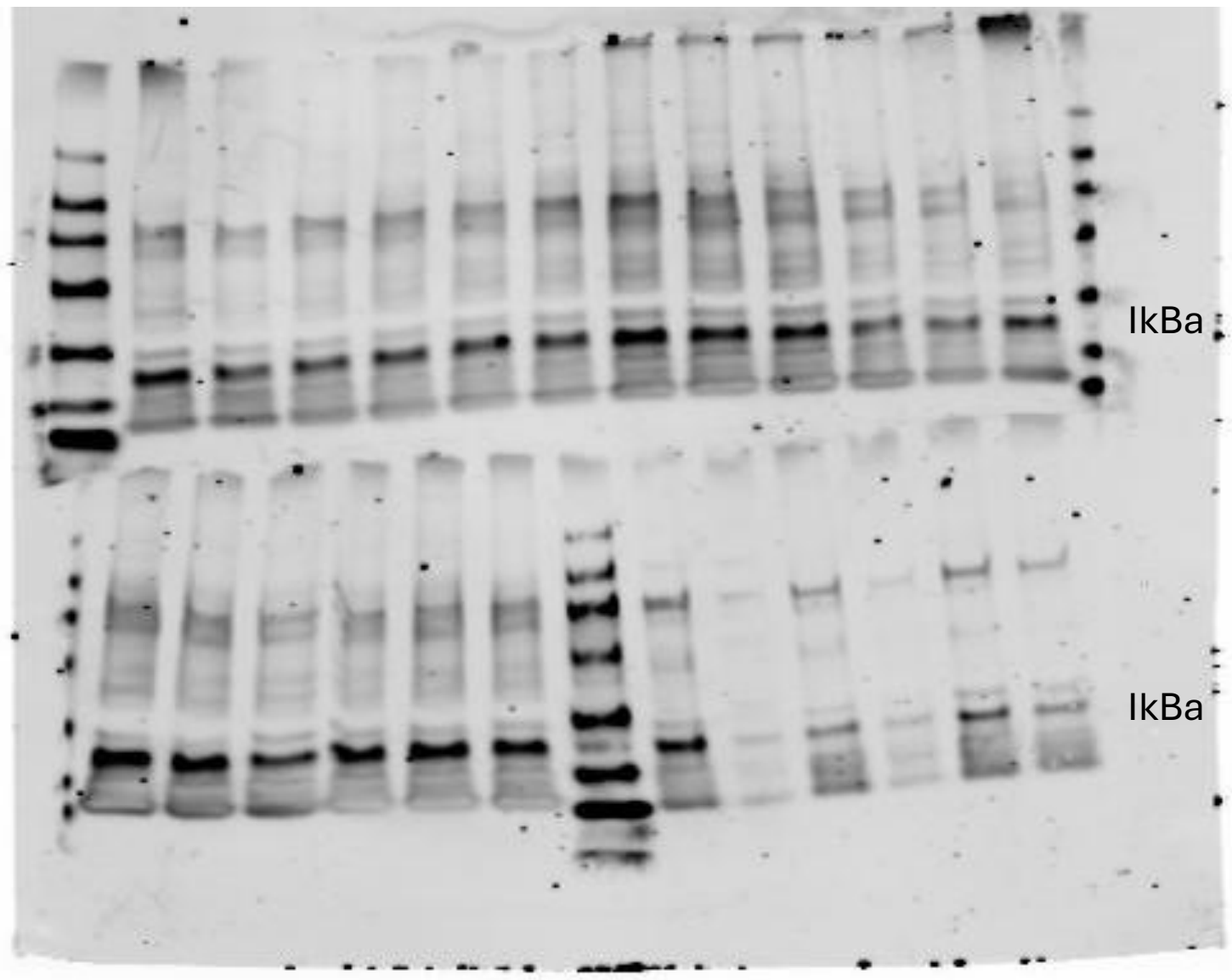

Lane #	Contents (n2)
1	Licor Chameleon Duo Ladder
2	Whole Cell Lysate HEK Blue TLR1 602S – Unstimulated n2
3	Whole Cell Lysate HEK Blue TLR1 602S – 30” 5 μm Pam3CSK4 Stimulated n2
4	Whole Cell Lysate HEK Blue TLR1 602S – 60” 5 μm Pam3CSK4 Stimulated n2
5	Whole Cell Lysate HEK Blue TLR1 602S – 90” 5 μm Pam3CSK4 Stimulated n2
6	Whole Cell Lysate HEK Blue TLR1 602S – 120” 5 μm Pam3CSK4 Stimulated n2
7	Whole Cell Lysate HEK Blue TLR1 602S – 180” 5 μm Pam3CSK4 Stimulated n2
8	Whole Cell Lysate HEK Blue TLR1 602I – Unstimulated n2
9	Whole Cell Lysate HEK Blue TLR1 602I – 30” 5 μm Pam3CSK4 Stimulated n2
10	Whole Cell Lysate HEK Blue TLR1 602I – 60” 5 μm Pam3CSK4 Stimulated n2
11	Whole Cell Lysate HEK Blue TLR1 602I – 90” 5 μm Pam3CSK4 Stimulated n2
12	Whole Cell Lysate HEK Blue TLR1 602I – 120” 5 μm Pam3CSK4 Stimulated n2
13	Whole Cell Lysate HEK Blue TLR1 602I – 180” 5 μm Pam3CSK4 Stimulated n2
14 (partial)	Licor Chameleon Duo Ladder

n3 Lane #	Contents (n3)
1 (partial)	Licor Chameleon Duo Ladder
2	Whole Cell Lysate HEK Blue TLR1 602S – Unstimulated n3
3	Whole Cell Lysate HEK Blue TLR1 602S – 30” 5 μm Pam3CSK4 Stimulated n3
4	Whole Cell Lysate HEK Blue TLR1 602S – 60” 5 μm Pam3CSK4 Stimulated n3
5	Whole Cell Lysate HEK Blue TLR1 602S – 90” 5 μm Pam3CSK4 Stimulated n3
6	Whole Cell Lysate HEK Blue TLR1 602S – 120” 5 μm Pam3CSK4 Stimulated n3
7	Whole Cell Lysate HEK Blue TLR1 602S – 180” 5 μm Pam3CSK4 Stimulated n3
8	Licor Chameleon Duo Ladder
9	Whole Cell Lysate HEK Blue TLR1 602I – Unstimulated n3
10	Whole Cell Lysate HEK Blue TLR1 602I – 30” 5 μm Pam3CSK4 Stimulated n3
11	Whole Cell Lysate HEK Blue TLR1 602I – 60” 5 μm Pam3CSK4 Stimulated n3
12	Whole Cell Lysate HEK Blue TLR1 602I – 90” 5 μm Pam3CSK4 Stimulated n3
13	Whole Cell Lysate HEK Blue TLR1 602I – 120” 5 μm Pam3CSK4 Stimulated n3
14	Whole Cell Lysate HEK Blue TLR1 602I – 180” 5 μm Pam3CSK4 Stimulated n3

Fig4C n2 (top) and n3 (bottom)
Channel 800

Licor Odyssey F Imager

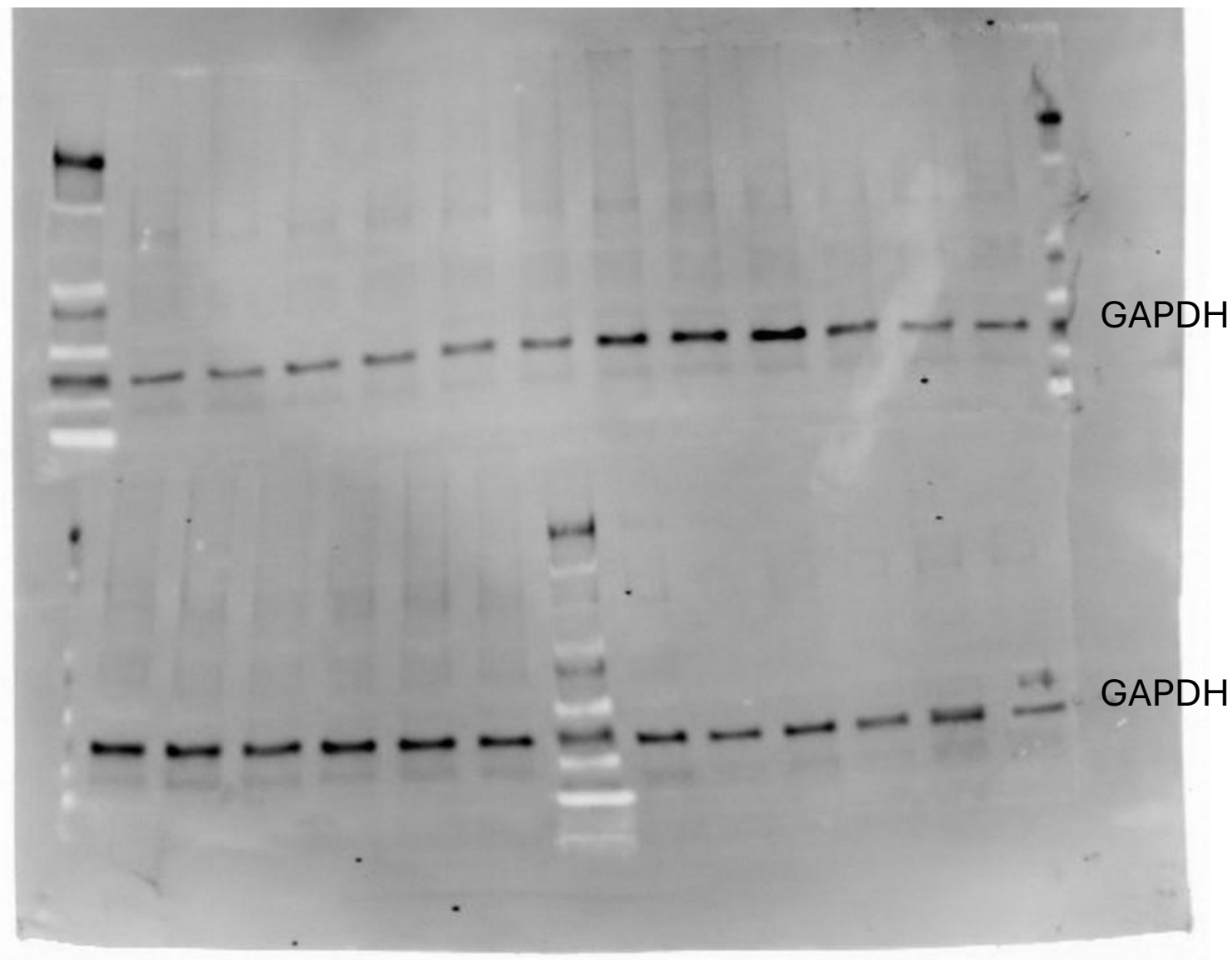

Lane #	Contents (n2)
1	Licor Chameleon Duo Ladder
2	Whole Cell Lysate HEK Blue TLR1 602S – Unstimulated n2
3	Whole Cell Lysate HEK Blue TLR1 602S – 30” 5 µm Pam3CSK4 Stimulated n2
4	Whole Cell Lysate HEK Blue TLR1 602S – 60” 5 µm Pam3CSK4 Stimulated n2
5	Whole Cell Lysate HEK Blue TLR1 602S – 90” 5 µm Pam3CSK4 Stimulated n2
6	Whole Cell Lysate HEK Blue TLR1 602S – 120” 5 µm Pam3CSK4 Stimulated n2
7	Whole Cell Lysate HEK Blue TLR1 602S – 180” 5 µm Pam3CSK4 Stimulated n2
8	Whole Cell Lysate HEK Blue TLR1 602I – Unstimulated n2
9	Whole Cell Lysate HEK Blue TLR1 602I – 30” 5 µm Pam3CSK4 Stimulated n2
10	Whole Cell Lysate HEK Blue TLR1 602I – 60” 5 µm Pam3CSK4 Stimulated n2
11	Whole Cell Lysate HEK Blue TLR1 602I – 90” 5 µm Pam3CSK4 Stimulated n2
12	Whole Cell Lysate HEK Blue TLR1 602I – 120” 5 µm Pam3CSK4 Stimulated n2
13	Whole Cell Lysate HEK Blue TLR1 602I – 180” 5 µm Pam3CSK4 Stimulated n2
14 (partial)	Licor Chameleon Duo Ladder

n3 Lane #	Contents (n3)
1 (partial)	Licor Chameleon Duo Ladder
2	Whole Cell Lysate HEK Blue TLR1 602S – Unstimulated n3
3	Whole Cell Lysate HEK Blue TLR1 602S – 30” 5 µm Pam3CSK4 Stimulated n3
4	Whole Cell Lysate HEK Blue TLR1 602S – 60” 5 µm Pam3CSK4 Stimulated n3
5	Whole Cell Lysate HEK Blue TLR1 602S – 90” 5 µm Pam3CSK4 Stimulated n3
6	Whole Cell Lysate HEK Blue TLR1 602S – 120” 5 µm Pam3CSK4 Stimulated n3
7	Whole Cell Lysate HEK Blue TLR1 602S – 180” 5 µm Pam3CSK4 Stimulated n3
8	Licor Chameleon Duo Ladder
9	Whole Cell Lysate HEK Blue TLR1 602I – Unstimulated n3
10	Whole Cell Lysate HEK Blue TLR1 602I – 30” 5 µm Pam3CSK4 Stimulated n3
11	Whole Cell Lysate HEK Blue TLR1 602I – 60” 5 µm Pam3CSK4 Stimulated n3
12	Whole Cell Lysate HEK Blue TLR1 602I – 90” 5 µm Pam3CSK4 Stimulated n3
13	Whole Cell Lysate HEK Blue TLR1 602I – 120” 5 µm Pam3CSK4 Stimulated n3
14	Whole Cell Lysate HEK Blue TLR1 602I – 180” 5 µm Pam3CSK4 Stimulated n3

Fig4D Representative and Fig4E n1
Channel 700

Licor Odyssey F Imager

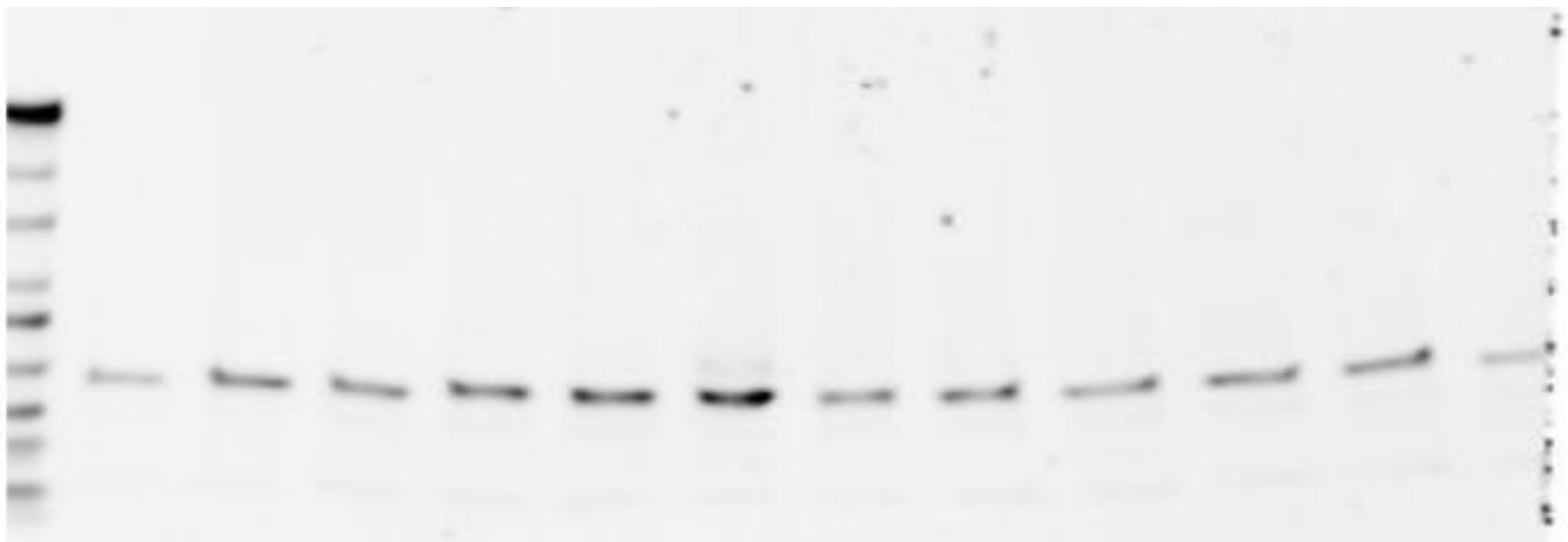

Lane #	Contents
1	Licor Chameleon Duo Ladder
2	Nuclear Enriched Lysate HEK Blue TLR1 602S – Unstimulated n1
3	Nuclear Enriched Lysate HEK Blue TLR1 602S – 30” 5 µm Pam3CSK4 Stimulated n1
4	Nuclear Enriched Lysate HEK Blue TLR1 602S – 60” 5 µm Pam3CSK4 Stimulated n1
5	Nuclear Enriched Lysate HEK Blue TLR1 602S – 90” 5 µm Pam3CSK4 Stimulated n1
6	Nuclear Enriched Lysate HEK Blue TLR1 602S – 120” 5 µm Pam3CSK4 Stimulated n1
7	Nuclear Enriched Lysate HEK Blue TLR1 602S – 180” 5 µm Pam3CSK4 Stimulated n1
8	Nuclear Enriched Lysate HEK Blue TLR1 602I – Unstimulated n1
9	Nuclear Enriched Lysate HEK Blue TLR1 602I – 30” 5 µm Pam3CSK4 Stimulated n1
10	Nuclear Enriched Lysate HEK Blue TLR1 602I – 60” 5 µm Pam3CSK4 Stimulated n1
11	Nuclear Enriched Lysate HEK Blue TLR1 602I – 90” 5 µm Pam3CSK4 Stimulated n1
12	Nuclear Enriched Lysate HEK Blue TLR1 602I – 120” 5 µm Pam3CSK4 Stimulated n1
13	Nuclear Enriched Lysate HEK Blue TLR1 602I – 180” 5 µm Pam3CSK4 Stimulated n1

Fig4D Representative and Fig4E n1
Channel 800

Licor Odyssey F Imager

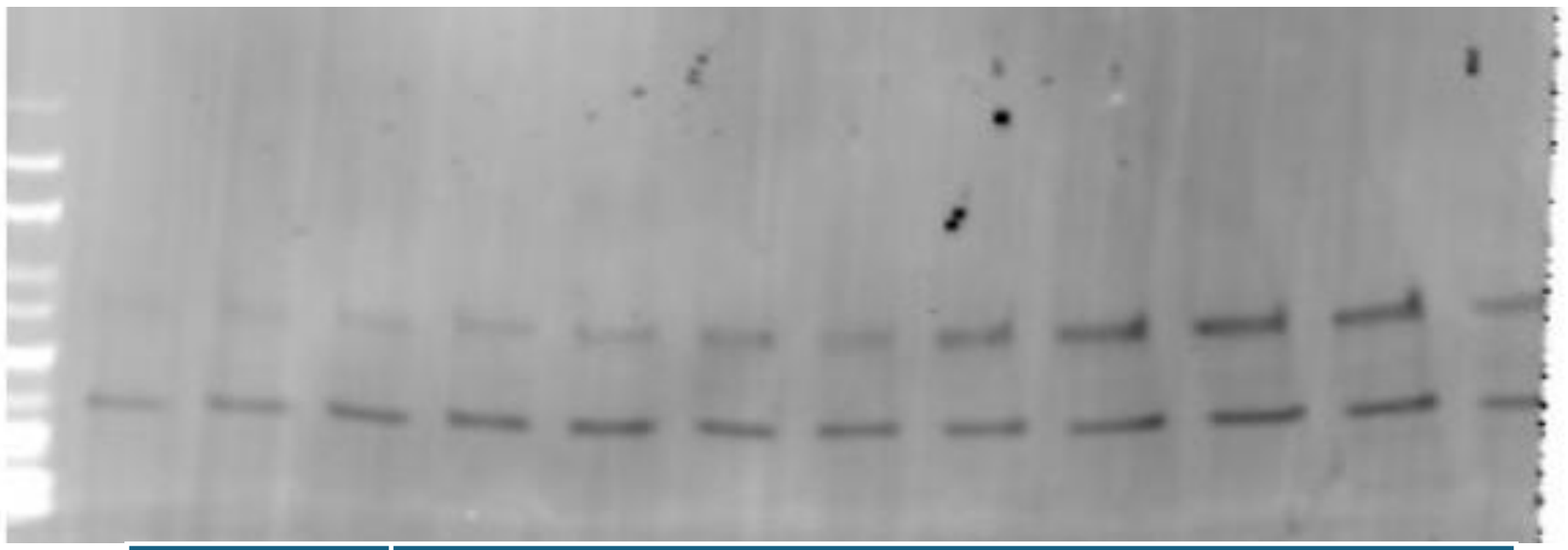

Lane #	Contents
1	Licor Chameleon Duo Ladder
2	Nuclear Enriched Lysate HEK Blue TLR1 602S – Unstimulated n1
3	Nuclear Enriched Lysate HEK Blue TLR1 602S – 30” 5 μm Pam3CSK4 Stimulated n1
4	Nuclear Enriched Lysate HEK Blue TLR1 602S – 60” 5 μm Pam3CSK4 Stimulated n1
5	Nuclear Enriched Lysate HEK Blue TLR1 602S – 90” 5 μm Pam3CSK4 Stimulated n1
6	Nuclear Enriched Lysate HEK Blue TLR1 602S – 120” 5 μm Pam3CSK4 Stimulated n1
7	Nuclear Enriched Lysate HEK Blue TLR1 602S – 180” 5 μm Pam3CSK4 Stimulated n1
8	Nuclear Enriched Lysate HEK Blue TLR1 602I – Unstimulated n1
9	Nuclear Enriched Lysate HEK Blue TLR1 602I – 30” 5 μm Pam3CSK4 Stimulated n1
10	Nuclear Enriched Lysate HEK Blue TLR1 602I – 60” 5 μm Pam3CSK4 Stimulated n1
11	Nuclear Enriched Lysate HEK Blue TLR1 602I – 90” 5 μm Pam3CSK4 Stimulated n1
12	Nuclear Enriched Lysate HEK Blue TLR1 602I – 120” 5 μm Pam3CSK4 Stimulated n1
13	Nuclear Enriched Lysate HEK Blue TLR1 602I – 180” 5 μm Pam3CSK4 Stimulated n1

Fig4D Representative
Channel 700

Licor Odyssey F Imager

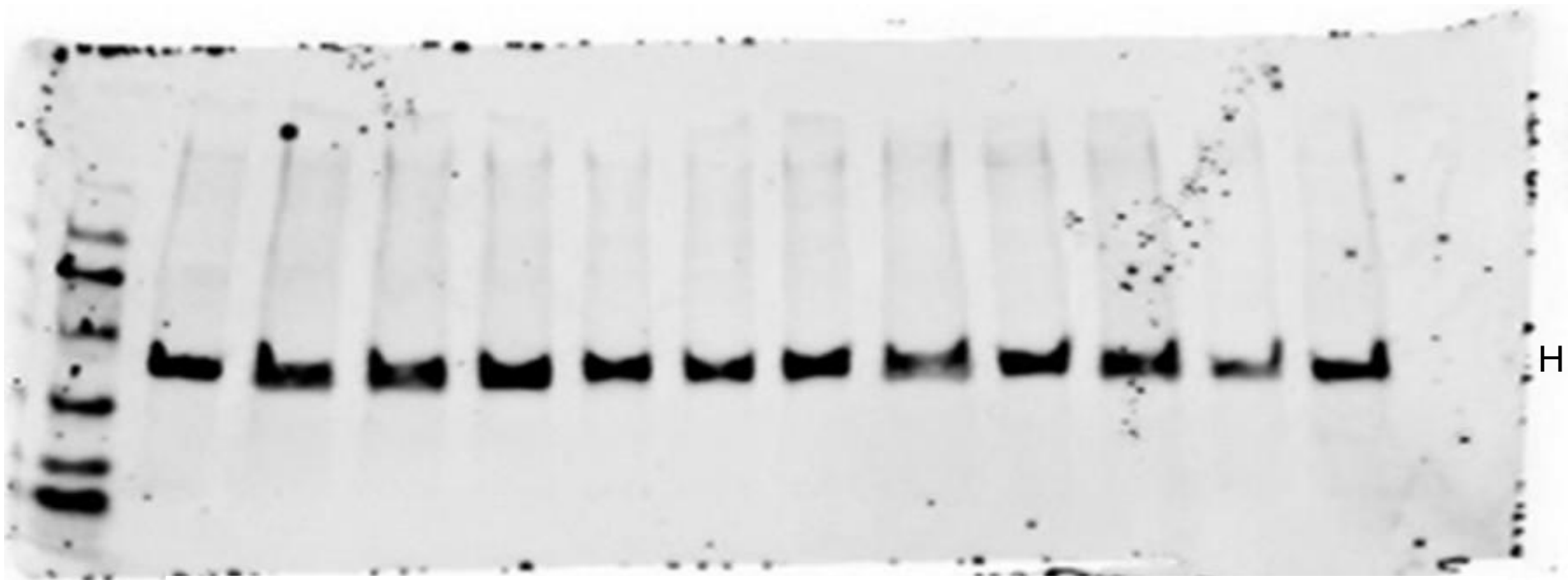

Lane #	Contents
1	Licor Chameleon Duo Ladder
2	Nuclear Enriched Lysate HEK Blue TLR1 602S – Unstimulated n1
3	Nuclear Enriched Lysate HEK Blue TLR1 602S – 30" 5 µm Pam3CSK4 Stimulated n1
4	Nuclear Enriched Lysate HEK Blue TLR1 602S – 60" 5 µm Pam3CSK4 Stimulated n1
5	Nuclear Enriched Lysate HEK Blue TLR1 602S – 90" 5 µm Pam3CSK4 Stimulated n1
6	Nuclear Enriched Lysate HEK Blue TLR1 602S – 120" 5 µm Pam3CSK4 Stimulated n1
7	Nuclear Enriched Lysate HEK Blue TLR1 602S – 180" 5 µm Pam3CSK4 Stimulated n1
8	Nuclear Enriched Lysate HEK Blue TLR1 602I – Unstimulated n1
9	Nuclear Enriched Lysate HEK Blue TLR1 602I – 30" 5 µm Pam3CSK4 Stimulated n1
10	Nuclear Enriched Lysate HEK Blue TLR1 602I – 60" 5 µm Pam3CSK4 Stimulated n1
11	Nuclear Enriched Lysate HEK Blue TLR1 602I – 90" 5 µm Pam3CSK4 Stimulated n1
12	Nuclear Enriched Lysate HEK Blue TLR1 602I – 120" 5 µm Pam3CSK4 Stimulated n1
13	Nuclear Enriched Lysate HEK Blue TLR1 602I – 180" 5 µm Pam3CSK4 Stimulated n1

Fig4D Representative
Channel 800

Licor Odyssey F Imager

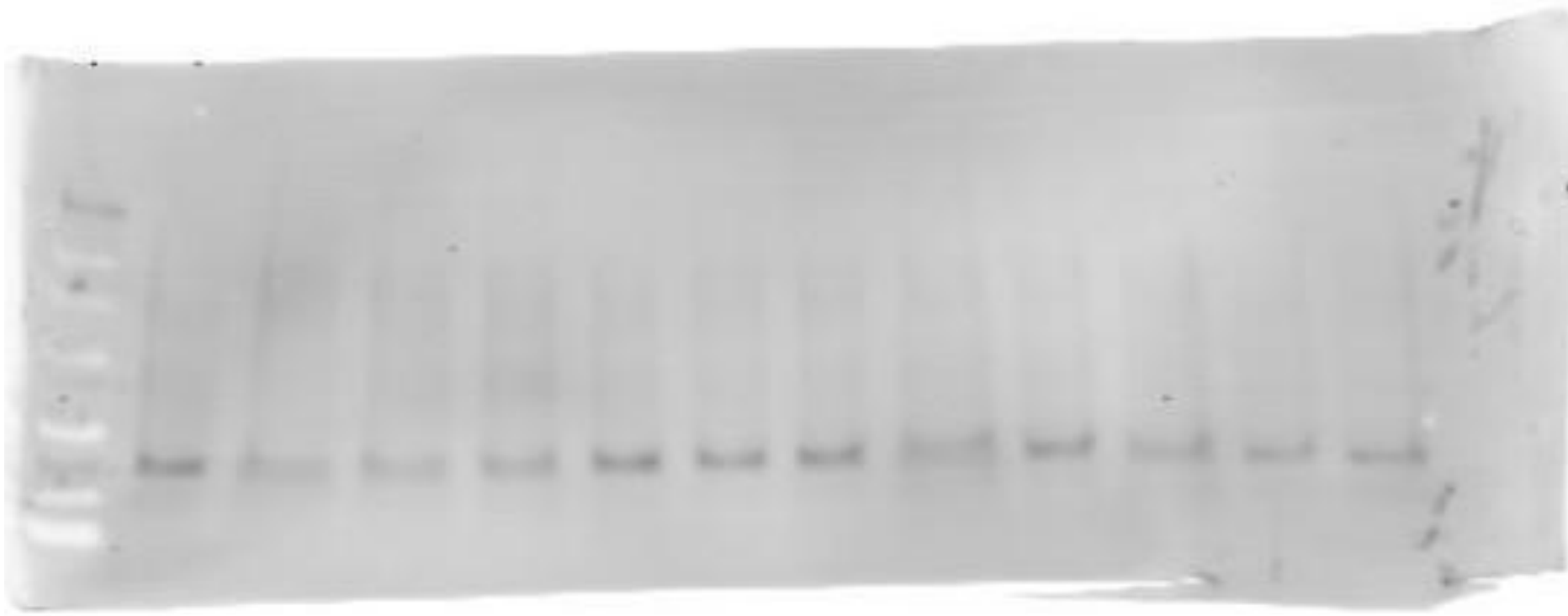

Lane #	Contents
1	Licor Chameleon Duo Ladder
2	Nuclear Enriched Lysate HEK Blue TLR1 602S – Unstimulated n1
3	Nuclear Enriched Lysate HEK Blue TLR1 602S – 30” 5 µm Pam3CSK4 Stimulated n1
4	Nuclear Enriched Lysate HEK Blue TLR1 602S – 60” 5 µm Pam3CSK4 Stimulated n1
5	Nuclear Enriched Lysate HEK Blue TLR1 602S – 90” 5 µm Pam3CSK4 Stimulated n1
6	Nuclear Enriched Lysate HEK Blue TLR1 602S – 120” 5 µm Pam3CSK4 Stimulated n1
7	Nuclear Enriched Lysate HEK Blue TLR1 602S – 180” 5 µm Pam3CSK4 Stimulated n1
8	Nuclear Enriched Lysate HEK Blue TLR1 602I – Unstimulated n1
9	Nuclear Enriched Lysate HEK Blue TLR1 602I – 30” 5 µm Pam3CSK4 Stimulated n1
10	Nuclear Enriched Lysate HEK Blue TLR1 602I – 60” 5 µm Pam3CSK4 Stimulated n1
11	Nuclear Enriched Lysate HEK Blue TLR1 602I – 90” 5 µm Pam3CSK4 Stimulated n1
12	Nuclear Enriched Lysate HEK Blue TLR1 602I – 120” 5 µm Pam3CSK4 Stimulated n1
13	Nuclear Enriched Lysate HEK Blue TLR1 602I – 180” 5 µm Pam3CSK4 Stimulated n1

Fig4E n2
Channel 700

Licor Odyssey F Imager

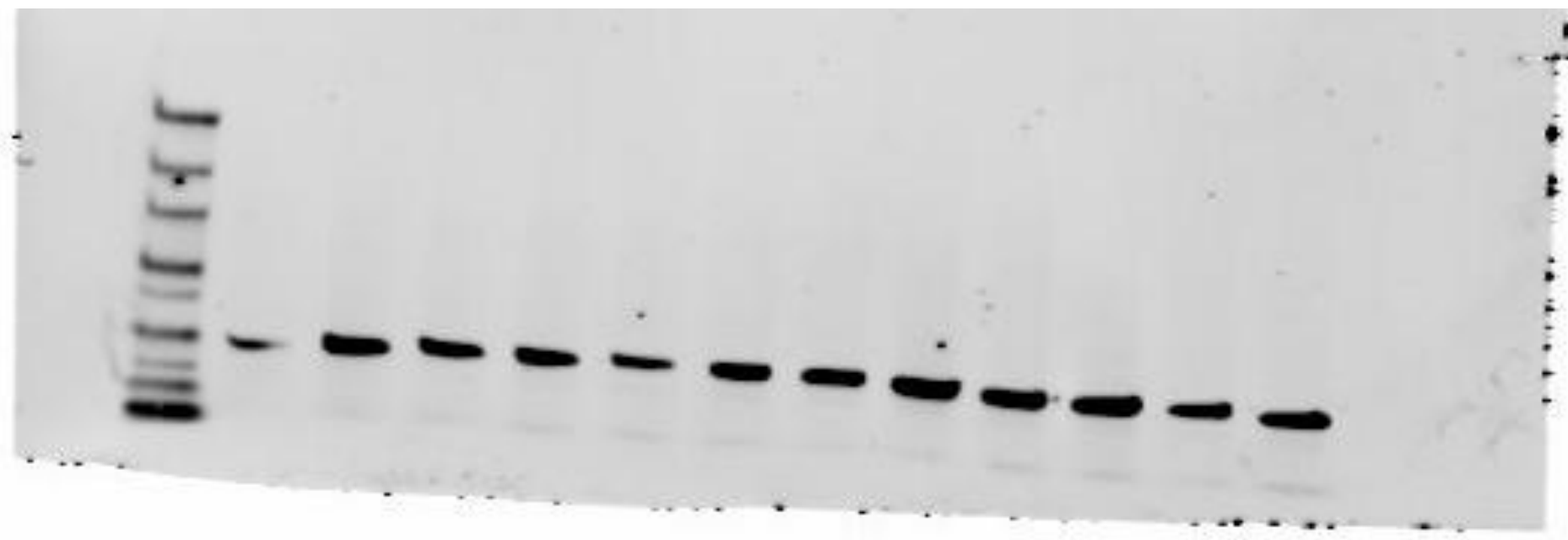

Lane #	Contents
1	Licor Chameleon Duo Ladder
2	Nuclear Enriched Lysate HEK Blue TLR1 602S – Unstimulated n2
3	Nuclear Enriched Lysate HEK Blue TLR1 602S – 30” 5 µm Pam3CSK4 Stimulated n2
4	Nuclear Enriched Lysate HEK Blue TLR1 602S – 60” 5 µm Pam3CSK4 Stimulated n2
5	Nuclear Enriched Lysate HEK Blue TLR1 602S – 90” 5 µm Pam3CSK4 Stimulated n2
6	Nuclear Enriched Lysate HEK Blue TLR1 602S – 120” 5 µm Pam3CSK4 Stimulated n2
7	Nuclear Enriched Lysate HEK Blue TLR1 602S – 180” 5 µm Pam3CSK4 Stimulated n2
8	Nuclear Enriched Lysate HEK Blue TLR1 602I – Unstimulated n2
9	Nuclear Enriched Lysate HEK Blue TLR1 602I – 30” 5 µm Pam3CSK4 Stimulated n2
10	Nuclear Enriched Lysate HEK Blue TLR1 602I – 60” 5 µm Pam3CSK4 Stimulated n2
11	Nuclear Enriched Lysate HEK Blue TLR1 602I – 90” 5 µm Pam3CSK4 Stimulated n2
12	Nuclear Enriched Lysate HEK Blue TLR1 602I – 120” 5 µm Pam3CSK4 Stimulated n2
13	Nuclear Enriched Lysate HEK Blue TLR1 602I – 180” 5 µm Pam3CSK4 Stimulated n2

Fig4E n2

Channel 800

Licor Odyssey F Imager

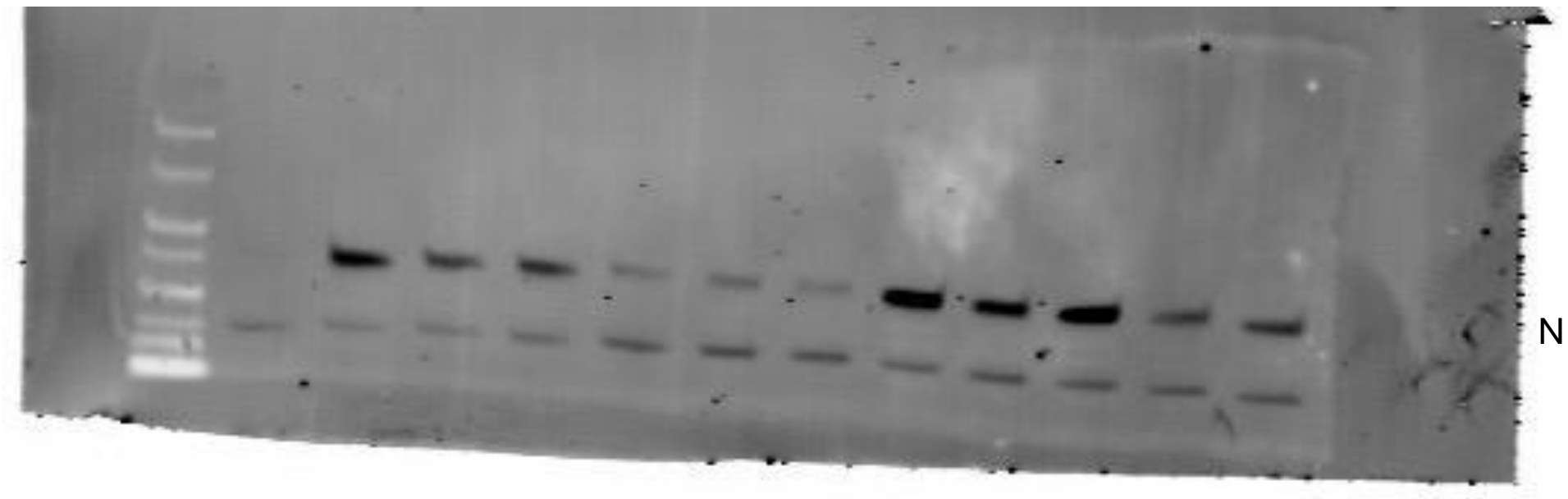

Lane #	Contents
1	Licor Chameleon Duo Ladder
2	Nuclear Enriched Lysate HEK Blue TLR1 602S – Unstimulated n2
3	Nuclear Enriched Lysate HEK Blue TLR1 602S – 30” 5 µm Pam3CSK4 Stimulated n2
4	Nuclear Enriched Lysate HEK Blue TLR1 602S – 60” 5 µm Pam3CSK4 Stimulated n2
5	Nuclear Enriched Lysate HEK Blue TLR1 602S – 90” 5 µm Pam3CSK4 Stimulated n2
6	Nuclear Enriched Lysate HEK Blue TLR1 602S – 120” 5 µm Pam3CSK4 Stimulated n2
7	Nuclear Enriched Lysate HEK Blue TLR1 602S – 180” 5 µm Pam3CSK4 Stimulated n2
8	Nuclear Enriched Lysate HEK Blue TLR1 602I – Unstimulated n2
9	Nuclear Enriched Lysate HEK Blue TLR1 602I – 30” 5 µm Pam3CSK4 Stimulated n2
10	Nuclear Enriched Lysate HEK Blue TLR1 602I – 60” 5 µm Pam3CSK4 Stimulated n2
11	Nuclear Enriched Lysate HEK Blue TLR1 602I – 90” 5 µm Pam3CSK4 Stimulated n2
12	Nuclear Enriched Lysate HEK Blue TLR1 602I – 120” 5 µm Pam3CSK4 Stimulated n2
13	Nuclear Enriched Lysate HEK Blue TLR1 602I – 180” 5 µm Pam3CSK4 Stimulated n2

Fig4E n3
Channel 700

Licor Odyssey F Imager

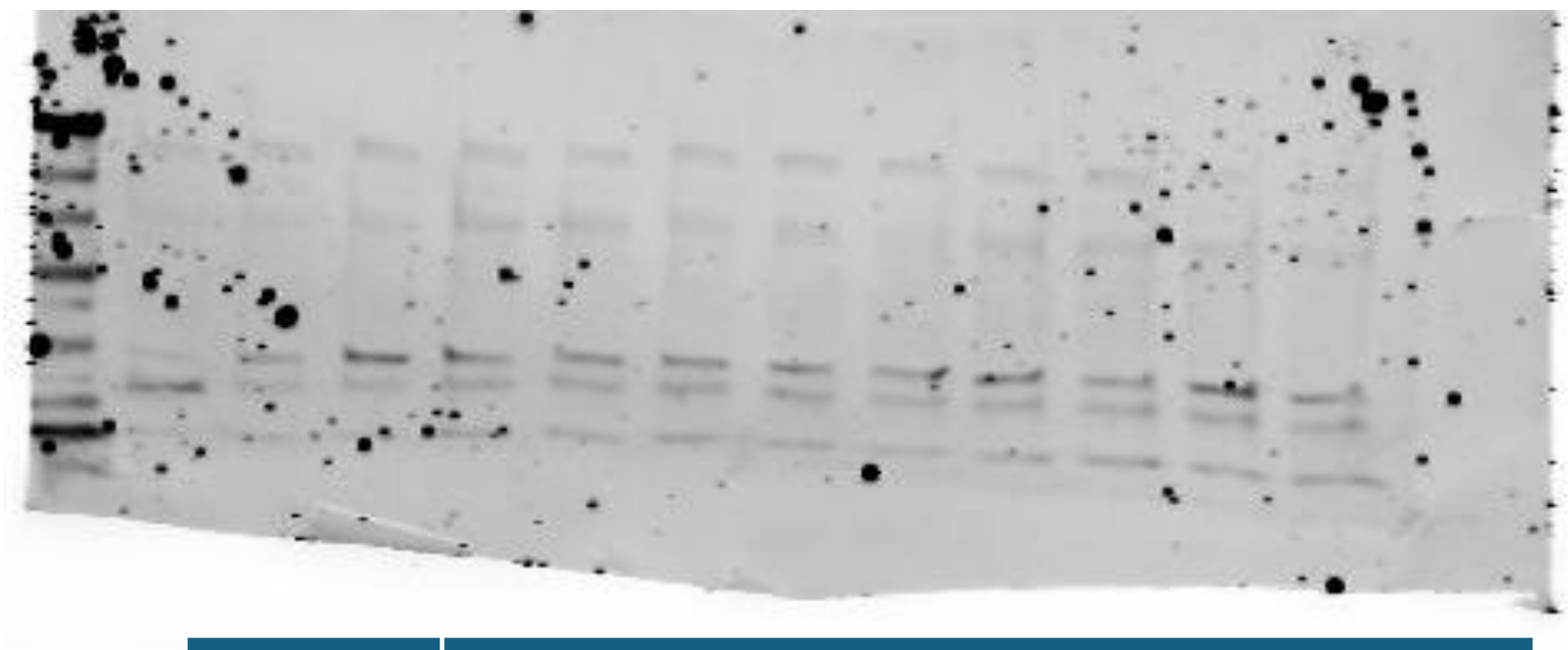

Lane #	Contents
1	Licor Chameleon Duo Ladder
2	Nuclear Enriched Lysate HEK Blue TLR1 602S – Unstimulated n3
3	Nuclear Enriched Lysate HEK Blue TLR1 602S – 30” 5 µm Pam3CSK4 Stimulated n3
4	Nuclear Enriched Lysate HEK Blue TLR1 602S – 60” 5 µm Pam3CSK4 Stimulated n3
5	Nuclear Enriched Lysate HEK Blue TLR1 602S – 90” 5 µm Pam3CSK4 Stimulated n3
6	Nuclear Enriched Lysate HEK Blue TLR1 602S – 120” 5 µm Pam3CSK4 Stimulated n3
7	Nuclear Enriched Lysate HEK Blue TLR1 602S – 180” 5 µm Pam3CSK4 Stimulated n3
8	Nuclear Enriched Lysate HEK Blue TLR1 602I – Unstimulated n3
9	Nuclear Enriched Lysate HEK Blue TLR1 602I – 30” 5 µm Pam3CSK4 Stimulated n3
10	Nuclear Enriched Lysate HEK Blue TLR1 602I – 60” 5 µm Pam3CSK4 Stimulated n3
11	Nuclear Enriched Lysate HEK Blue TLR1 602I – 90” 5 µm Pam3CSK4 Stimulated n3
12	Nuclear Enriched Lysate HEK Blue TLR1 602I – 120” 5 µm Pam3CSK4 Stimulated n3
13	Nuclear Enriched Lysate HEK Blue TLR1 602I – 180” 5 µm Pam3CSK4 Stimulated n3

Fig4E n3
Channel 800

Licor Odyssey F Imager

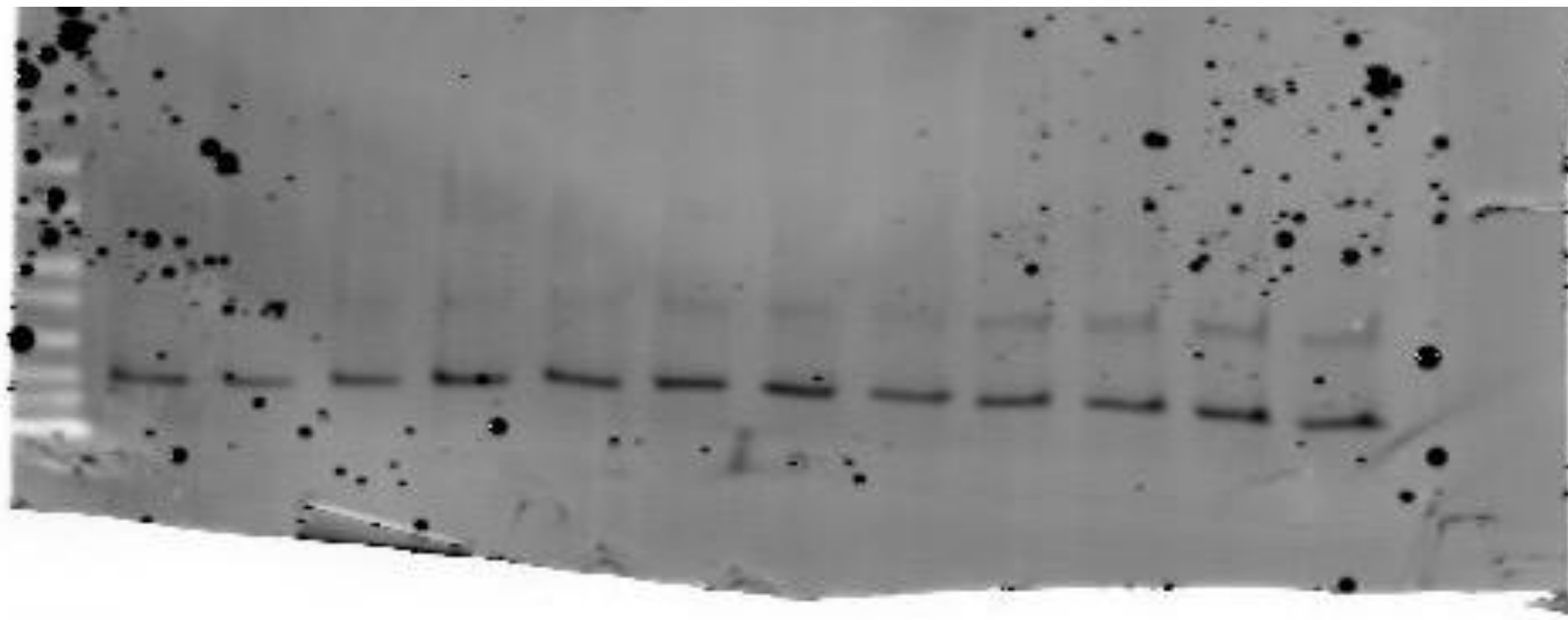

NF-kB p65

Lane #	Contents
1	Licor Chameleon Duo Ladder
2	Nuclear Enriched Lysate HEK Blue TLR1 602S – Unstimulated n3
3	Nuclear Enriched Lysate HEK Blue TLR1 602S – 30” 5 µm Pam3CSK4 Stimulated n3
4	Nuclear Enriched Lysate HEK Blue TLR1 602S – 60” 5 µm Pam3CSK4 Stimulated n3
5	Nuclear Enriched Lysate HEK Blue TLR1 602S – 90” 5 µm Pam3CSK4 Stimulated n3
6	Nuclear Enriched Lysate HEK Blue TLR1 602S – 120” 5 µm Pam3CSK4 Stimulated n3
7	Nuclear Enriched Lysate HEK Blue TLR1 602S – 180” 5 µm Pam3CSK4 Stimulated n3
8	Nuclear Enriched Lysate HEK Blue TLR1 602I – Unstimulated n3
9	Nuclear Enriched Lysate HEK Blue TLR1 602I – 30” 5 µm Pam3CSK4 Stimulated n3
10	Nuclear Enriched Lysate HEK Blue TLR1 602I – 60” 5 µm Pam3CSK4 Stimulated n3
11	Nuclear Enriched Lysate HEK Blue TLR1 602I – 90” 5 µm Pam3CSK4 Stimulated n3
12	Nuclear Enriched Lysate HEK Blue TLR1 602I – 120” 5 µm Pam3CSK4 Stimulated n3
13	Nuclear Enriched Lysate HEK Blue TLR1 602I – 180” 5 µm Pam3CSK4 Stimulated n3
