# Supplementary material for: Endosome restricted human TLR1 polymorphism highlights a role for MyD88 in Pam3CSK4 detection
Source: PLoS One. 2026 Oct 5;21(10):e0358252. doi: 10.1371/journal.pone.0358252 (PMC13638051; doi:10.1371/journal.pone.0358252)
Supplement: Supplementary file 2 [file pone.0358252.s004.docx]

>Addgene pCMV-3Tag-3a

ATGCATTAGT TATTAATAGT AATCAATTAC GGGGTCATTA GTTCATAGCC

51 CATATATGGA GTTCCGCGTT ACATAACTTA CGGTAAATGG CCCGCCTGGC

101 TGACCGCCCA ACGACCCCCG CCCATTGACG TCAATAATGA CGTATGTTCC

151 CATAGTAACG CCAATAGGGA CTTTCCATTG ACGTCAATGG GTGGAGTATT

201 TACGGTAAAC TGCCCACTTG GCAGTACATC AAGTGTATCA TATGCCAAGT

251 ACGCCCCCTA TTGACGTCAA TGACGGTAAA TGGCCCGCCT GGCATTATGC

301 CCAGTACATG ACCTTATGGG ACTTTCCTAC TTGGCAGTAC ATCTACGTAT

351 TAGTCATCGC TATTACCATG GTGATGCGGT TTTGGCAGTA CATCAATGGG

401 CGTGGATAGC GGTTTGACTC ACGGGGATTT CCAAGTCTCC ACCCCATTGA

451 CGTCAATGGG AGTTTGTTTT GGCACCAAAA TCAACGGGAC TTTCCAAAAT

501 GTCGTAACAA CTCCGCCCCA TTGACGCAAA TGGGCGGTAG GCGTGTACGG

551 TGGGAGGTCT ATATAAGCAG AGCTGGTTTA GTGAACCGTC AGATCCGCTA

601 GCGATTACGC CAAGCTCGAA ATTAACCCTC ACTAAAGGGA ACAAAAGCTG

651 GAGCTCCACC GCGGTGGCGG CCGCTCTAGC CCGGGCGGAT CCCCCGGGCT

701 GCAGGAATTC GATATCAAGC TTATCGATAC CGTCGACCTC GAGGATTACA

751 AGGATGACGA CGATAAGGAC TATAAGGACG ATGATGACAA GGACTACAAA

801 GATGATGACG ATAAATAGGG CCCGGTACCT TAATTAATTA AGGTACCAGG

851 TAAGTGTACC CAATTCGCCC TATAGTGAGT CGTATTACAA TTCACTCGAT

901 CGGCTCGCTG ATCAGCCTCG ACTGTGCCTT CTAGTTGCCA GCCATCTGTT

951 GTTTGCCCCT CCCCCGTGCC TTCCTTGACC CTGGAAGGTG CCACTCCCAC

1001 TGTCCTTTCC TAATAAAATG AGGAAATTGC ATCGCATTGT CTGAGTAGGT

1051 GTCATTCTAT TCTGGGGGGT GGGGTGGGGC AGGACAGCAA GGGGGAGGAT

1101 TGGGAAGACA ATAGCAGGCA TGCTGGGGAA CGCGTAAATT GTAAGCGTTA

1151 ATATTTTGTT AAAATTCGCG TTAAATTTTT GTTAAATCAG CTCATTTTTT

1201 AACCAATAGG CCGAAATCGG CAAAATCCCT TATAAATCAA AAGAATAGAC

1251 CGAGATAGGG TTGAGTGTTG TTCCAGTTTG GAACAAGAGT CCACTATTAA

1301 AGAACGTGGA CTCCAACGTC AAAGGGCGAA AAACCGTCTA TCAGGGCGAT

1351 GGCCCACTAC GTGAACCATC ACCCTAATCA AGTTTTTTGG GGTCGAGGTG

1401 CCGTAAAGCA CTAAATCGGA ACCCTAAAGG GAGCCCCCGA TTTAGAGCTT

1451 GACGGGGAAA GCCGGCGAAC GTGGCGAGAA AGGAAGGGAA GAAAGCGAAA

1501 GGAGCGGGCG CTAGGGCGCT GGCAAGTGTA GCGGTCACGC TGCGCGTAAC

1551 CACCACACCC GCCGCGCTTA ATGCGCCGCT ACAGGGCGCG TCAGGTGGCA

1601 CTTTTCGGGG AAATGTGCGC GGAACCCCTA TTTGTTTATT TTTCTAAATA

1651 CATTCAAATA TGTATCCGCT CATGAGACAA TAACCCTGAT AAATGCTTCA

1701 ATAATATTGA AAAAGGAAGA ATCCTGAGGC GGAAAGAACC AGCTGTGGAA

1751 TGTGTGTCAG TTAGGGTGTG GAAAGTCCCC AGGCTCCCCA GCAGGCAGAA

1801 GTATGCAAAG CATGCATCTC AATTAGTCAG CAACCAGGTG TGGAAAGTCC

1851 CCAGGCTCCC CAGCAGGCAG AAGTATGCAA AGCATGCATC TCAATTAGTC

1901 AGCAACCATA GTCCCGCCCC TAACTCCGCC CATCCCGCCC CTAACTCCGC

1951 CCAGTTCCGC CCATTCTCCG CCCCATGGCT GACTAATTTT TTTTATTTAT

2001 GCAGAGGCCG AGGCCGCCTC GGCCTCTGAG CTATTCCAGA AGTAGTGAGG

2051 AGGCTTTTTT GGAGGCCTAG GCTTTTGCAA AGATCGATCA AGAGACAGGA

2101 TGAGGATCGT TTCGCATGAT TGAACAAGAT GGATTGCACG CAGGTTCTCC

2151 GGCCGCTTGG GTGGAGAGGC TATTCGGCTA TGACTGGGCA CAACAGACAA

2201 TCGGCTGCTC TGATGCCGCC GTGTTCCGGC TGTCAGCGCA GGGGCGCCCG

2251 GTTCTTTTTG TCAAGACCGA CCTGTCCGGT GCCCTGAATG AACTGCAAGA

2301 CGAGGCAGCG CGGCTATCGT GGCTGGCCAC GACGGGCGTT CCTTGCGCAG

2351 CTGTGCTCGA CGTTGTCACT GAAGCGGGAA GGGACTGGCT GCTATTGGGC

2401 GAAGTGCCGG GGCAGGATCT CCTGTCATCT CACCTTGCTC CTGCCGAGAA

2451 AGTATCCATC ATGGCTGATG CAATGCGGCG GCTGCATACG CTTGATCCGG

2501 CTACCTGCCC ATTCGACCAC CAAGCGAAAC ATCGCATCGA GCGAGCACGT

2551 ACTCGGATGG AAGCCGGTCT TGTCGATCAG GATGATCTGG ACGAAGAACA

2601 TCAGGGGCTC GCGCCAGCCG AACTGTTCGC CAGGCTCAAG GCGAGCATGC

2651 CCGACGGCGA GGATCTCGTC GTGACCCATG GCGATGCCTG CTTGCCGAAT

2701 ATCATGGTGG AAAATGGCCG CTTTTCTGGA TTCATCGACT GTGGCCGGCT

2751 GGGTGTGGCG GACCGCTATC AGGACATAGC GTTGGCTACC CGTGATATTG

2801 CTGAAGAACT TGGCGGCGAA TGGGCTGACC GCTTCCTCGT GCTTTACGGT

2851 ATCGCCGCTC CCGATTCGCA GCGCATCGCC TTCTATCGCC TTCTTGACGA

2901 GTTCTTCTGA GCGGGACTCT GGGGTTCGAA ATGACCGACC AAGCGACGCC

2951 CAACCTGCCA TCACGAGATT TCGATTCCAC CGCCGCCTTC TATGAAAGGT

3001 TGGGCTTCGG AATCGTTTTC CGGGACGCCG GCTGGATGAT CCTCCAGCGC

3051 GGGGATCTCA TGCTGGAGTT CTTCGCCCAC CCTAGGGGGA GGCTAACTGA

3101 AACACGGAAG GAGACAATAC CGGAAGGAAC CCGCGCTATG ACGGCAATAA

3151 AAAGACAGAA TAAAACGCAC GGTGTTGGGT CGTTTGTTCA TAAACGCGGG

3201 GTTCGGTCCC AGGGCTGGCA CTCTGTCGAT ACCCCACCGA GACCCCATTG

3251 GGGCCAATAC GCCCGCGTTT CTTCCTTTTC CCCACCCCAC CCCCCAAGTT

3301 CGGGTGAAGG CCCAGGGCTC GCAGCCAACG TCGGGGCGGC AGGCCCTGCC

3351 ATAGCCTCAG GTTACTCATA TATACTTTAG ATTGATTTAA AACTTCATTT

3401 TTAATTTAAA AGGATCTAGG TGAAGATCCT TTTTGATAAT CTCATGACCA

3451 AAATCCCTTA ACGTGAGTTT TCGTTCCACT GAGCGTCAGA CCCCGTAGAA

3501 AAGATCAAAG GATCTTCTTG AGATCCTTTT TTTCTGCGCG TAATCTGCTG

3551 CTTGCAAACA AAAAAACCAC CGCTACCAGC GGTGGTTTGT TTGCCGGATC

3601 AAGAGCTACC AACTCTTTTT CCGAAGGTAA CTGGCTTCAG CAGAGCGCAG

3651 ATACCAAATA CTGTTCTTCT AGTGTAGCCG TAGTTAGGCC ACCACTTCAA

3701 GAACTCTGTA GCACCGCCTA CATACCTCGC TCTGCTAATC CTGTTACCAG

3751 TGGCTGCTGC CAGTGGCGAT AAGTCGTGTC TTACCGGGTT GGACTCAAGA

3801 CGATAGTTAC CGGATAAGGC GCAGCGGTCG GGCTGAACGG GGGGTTCGTG

3851 CACACAGCCC AGCTTGGAGC GAACGACCTA CACCGAACTG AGATACCTAC

3901 AGCGTGAGCT ATGAGAAAGC GCCACGCTTC CCGAAGGGAG AAAGGCGGAC

3951 AGGTATCCGG TAAGCGGCAG GGTCGGAACA GGAGAGCGCA CGAGGGAGCT

4001 TCCAGGGGGA AACGCCTGGT ATCTTTATAG TCCTGTCGGG TTTCGCCACC

4051 TCTGACTTGA GCGTCGATTT TTGTGATGCT CGTCAGGGGG GCGGAGCCTA

4101 TGGAAAAACG CCAGCAACGC GGCCTTTTTA CGGTTCCTGG CCTTTTGCTG

4151 GCCTTTTGCT CACATGTTCT TTCCTGCGTT ATCCCCTGAT TCTGTGGATA

4201 ACCGTATTAC CGCC

>CCW pCMV-TLR1.602I-3FLAG

ATGCATTAGTTATTAATAGTAATCAATTACGGGGTCATTAGTTCATAGCCCATATATGGAGTTCCGCGTTACATAACTTACGGTAAATGGCCCGCCTGGCTGACCGCCCAACGACCCCCGCCCATTGACGTCAATAATGACGTATGTTCCCATAGTAACGCCAATAGGGACTTTCCATTGACGTCAATGGGTGGAGTATTTACGGTAAACTGCCCACTTGGCAGTACATCAAGTGTATCATATGCCAAGTACGCCCCCTATTGACGTCAATGACGGTAAATGGCCCGCCTGGCATTATGCCCAGTACATGACCTTATGGGACTTTCCTACTTGGCAGTACATCTACGTATTAGTCATCGCTATTACCATGGTGATGCGGTTTTGGCAGTACATCAATGGGCGTGGATAGCGGTTTGACTCACGGGGATTTCCAAGTCTCCACCCCATTGACGTCAATGGGAGTTTGTTTTGGCACCAAAATCAACGGGACTTTCCAAAATGTCGTAACAACTCCGCCCCATTGACGCAAATGGGCGGTAGGCGTGTACGGTGGGAGGTCTATATAAGCAGAGCTGGTTTAGTGAACCGTCAGATCCGCTAGCATGACATCCATCTTTCATTTCGCCATTATCTTCATGCTGATACTGCAGATCAGAATCCAGCTGAGCGAGGAAAGCGAGTTCCTGGTGGACAGAAGCAAGAACGGCCTGATTCACGTGCCTAAGGACCTGAGCCAAAAGACCACAATCCTGAATATCAGCCAGAACTACATCTCCGAGCTGTGGACAAGCGACATCCTGTCTCTCTCTAAGCTGCGGATCCTGATCATCAGCCACAATAGAATCCAGTACCTGGACATCAGCGTGTTCAAGTTCAATCAAGAGCTCGAATACCTCGACCTGTCTCACAACAAGCTGGTGAAGATTAGCTGTCACCCTACAGTGAACCTGAAGCACCTGGACCTGTCCTTTAACGCCTTTGACGCCCTGCCTATCTGTAAAGAATTCGGCAACATGAGCCAGCTGAAATTCCTAGGCCTGTCAACAACACACCTGGAGAAGTCTTCTGTTCTTCCTATCGCCCATCTGAATATCAGCAAGGTGCTGCTGGTGCTGGGCGAAACCTACGGCGAGAAGGAAGATCCTGAGGGCCTGCAGGACTTCAACACCGAGTCACTGCACATCGTCTTTCCAACAAACAAGGAATTCCACTTCATCCTGGACGTGAGTGTGAAGACAGTGGCCAACCTGGAGCTGTCTAATATTAAGTGCGTGCTGGAAGATAACAAGTGTAGTTACTTTCTGAGCATCCTGGCTAAACTGCAAACCAACCCCAAGCTGTCCAACCTGACCCTGAACAACATCGAGACCACCTGGAACAGCTTCATCCGGATCCTGCAGCTGGTGTGGCACACCACCGTATGGTATTTCAGCATCAGCAACGTGAAGCTCCAGGGCCAGCTGGACTTTAGAGATTTCGACTACTCCGGCACCAGCCTGAAGGCCCTGTCTATCCACCAGGTGGTGAGCGATGTGTTCGGATTCCCCCAATCTTACATCTACGAAATCTTTAGCAATATGAACATCAAGAACTTCACCGTGTCTGGAACAAGAATGGTGCACATGCTGTGTCCTTCTAAGATCAGTCCTTTCCTGCACCTGGATTTTAGCAACAACCTGCTCACAGACACCGTGTTCGAGAACTGCGGCCACCTGACAGAGTTGGAAACCCTGATCCTGCAGATGAACCAGCTGAAAGAGCTGTCAAAGATCGCCGAGATGACCACACAGATGAAGAGCCTGCAGCAGCTGGATATCAGCCAGAATTCCGTGTCCTACGACGAGAAAAAGGGCGATTGCAGCTGGACTAAGTCTCTTCTTAGCCTGAATATGTCTTCCAACATCCTGACCGACACCATCTTTCGGTGCCTGCCCCCTAGAATCAAGGTGCTGGACCTGCACAGCAACAAAATCAAGAGCATACCTAAGCAGGTGGTCAAGCTGGAAGCCCTGCAGGAGCTGAACGTGGCCTTCAACTCTCTGACCGACCTTCCTGGCTGCGGCAGCTTCAGCAGCCTGAGCGTGCTGATCATCGACCACAACAGCGTGAGCCATCCTAGCGCCGATTTCTTCCAAAGCTGCCAGAAGATGCGGAGCATCAAAGCCGGCGACAACCCTTTCCAGTGCACCTGTGAACTGGGAGAATTCGTCAAAAACATCGACCAGGTTTCTAGCGAAGTGCTGGAAGGCTGGCCTGATTCTTACAAGTGCGACTACCCCGAGAGCTATAGAGGCACCCTGCTGAAAGATTTCCACATGAGCGAACTGAGCTGCAACATAACCCTGCTGATCGTGACTATCGTGGCCACCATGCTGGTGCTCGCTGTGACAGTGACTTCCCTCTGTATCTACCTGGATCTGCCTTGGTACCTGAGGATGGTGTGCCAGTGGACCCAGACCAGAAGACGGGCCAGAAACATCCCTCTGGAGGAGCTGCAAAGAAATCTGCAGTTCCACGCCTTTATCTCTTATAGCGGACACGACAGCTTCTGGGTGAAGAACGAGCTGCTGCCAAACCTGGAAAAAGAGGGAATGCAGATCTGCCTGCACGAGCGGAACTTCGTCCCAGGCAAGTCCATCGTGGAGAACATCATCACCTGCATCGAGAAGTCCTACAAGAGCATCTTCGTGCTGAGCCCCAATTTCGTCCAGAGCGAGTGGTGCCACTACGAGCTGTACTTCGCTCATCACAACCTGTTCCACGAGGGCAGCAATAGCCTGATTCTGATCCTGCTGGAGCCTATCCCACAGTACAGCATCCCCAGCAGTTACCACAAGCTGAAGTCCCTGATGGCTAGACGCACCTACCTGGAATGGCCCAAGGAAAAATCTAAAAGAGGCTTGTTCTGGGCCAATCTGCGCGCCGCTATCAACATCAAGCTGACCGAGCAGGCCAAGAAGGTCGACCTCGAGGATTACAAGGATGACGACGATAAGGACTATAAGGACGATGATGACAAGGACTACAAAGATGATGACGATAAATAGGGCCCGGTACCTTAATTAATTAAGGTACCAGGTAAGTGTACCCAATTCGCCCTATAGTGAGTCGTATTACAATTCACTCGATCGGCTCGCTGATCAGCCTCGACTGTGCCTTCTAGTTGCCAGCCATCTGTTGTTTGCCCCTCCCCCGTGCCTTCCTTGACCCTGGAAGGTGCCACTCCCACTGTCCTTTCCTAATAAAATGAGGAAATTGCATCGCATTGTCTGAGTAGGTGTCATTCTATTCTGGGGGGTGGGGTGGGGCAGGACAGCAAGGGGGAGGATTGGGAAGACAATAGCAGGCATGCTGGGGAACGCGTAAATTGTAAGCGTTAATATTTTGTTAAAATTCGCGTTAAATTTTTGTTAAATCAGCTCATTTTTTAACCAATAGGCCGAAATCGGCAAAATCCCTTATAAATCAAAAGAATAGACCGAGATAGGGTTGAGTGTTGTTCCAGTTTGGAACAAGAGTCCACTATTAAAGAACGTGGACTCCAACGTCAAAGGGCGAAAAACCGTCTATCAGGGCGATGGCCCACTACGTGAACCATCACCCTAATCAAGTTTTTTGGGGTCGAGGTGCCGTAAAGCACTAAATCGGAACCCTAAAGGGAGCCCCCGATTTAGAGCTTGACGGGGAAAGCCGGCGAACGTGGCGAGAAAGGAAGGGAAGAAAGCGAAAGGAGCGGGCGCTAGGGCGCTGGCAAGTGTAGCGGTCACGCTGCGCGTAACCACCACACCCGCCGCGCTTAATGCGCCGCTACAGGGCGCGTCAGGTGGCACTTTTCGGGGAAATGTGCGCGGAACCCCTATTTGTTTATTTTTCTAAATACATTCAAATATGTATCCGCTCATGAGACAATAACCCTGATAAATGCTTCAATAATATTGAAAAAGGAAGAATCCTGAGGCGGAAAGAACCAGCTGTGGAATGTGTGTCAGTTAGGGTGTGGAAAGTCCCCAGGCTCCCCAGCAGGCAGAAGTATGCAAAGCATGCATCTCAATTAGTCAGCAACCAGGTGTGGAAAGTCCCCAGGCTCCCCAGCAGGCAGAAGTATGCAAAGCATGCATCTCAATTAGTCAGCAACCATAGTCCCGCCCCTAACTCCGCCCATCCCGCCCCTAACTCCGCCCAGTTCCGCCCATTCTCCGCCCCATGGCTGACTAATTTTTTTTATTTATGCAGAGGCCGAGGCCGCCTCGGCCTCTGAGCTATTCCAGAAGTAGTGAGGAGGCTTTTTTGGAGGCCTAGGCTTTTGCAAAGATCGATCAAGAGACAGGATGAGGATCGTTTCGCATGATTGAACAAGATGGATTGCACGCAGGTTCTCCGGCCGCTTGGGTGGAGAGGCTATTCGGCTATGACTGGGCACAACAGACAATCGGCTGCTCTGATGCCGCCGTGTTCCGGCTGTCAGCGCAGGGGCGCCCGGTTCTTTTTGTCAAGACCGACCTGTCCGGTGCCCTGAATGAACTGCAAGACGAGGCAGCGCGGCTATCGTGGCTGGCCACGACGGGCGTTCCTTGCGCAGCTGTGCTCGACGTTGTCACTGAAGCGGGAAGGGACTGGCTGCTATTGGGCGAAGTGCCGGGGCAGGATCTCCTGTCATCTCACCTTGCTCCTGCCGAGAAAGTATCCATCATGGCTGATGCAATGCGGCGGCTGCATACGCTTGATCCGGCTACCTGCCCATTCGACCACCAAGCGAAACATCGCATCGAGCGAGCACGTACTCGGATGGAAGCCGGTCTTGTCGATCAGGATGATCTGGACGAAGAACATCAGGGGCTCGCGCCAGCCGAACTGTTCGCCAGGCTCAAGGCGAGCATGCCCGACGGCGAGGATCTCGTCGTGACCCATGGCGATGCCTGCTTGCCGAATATCATGGTGGAAAATGGCCGCTTTTCTGGATTCATCGACTGTGGCCGGCTGGGTGTGGCGGACCGCTATCAGGACATAGCGTTGGCTACCCGTGATATTGCTGAAGAACTTGGCGGCGAATGGGCTGACCGCTTCCTCGTGCTTTACGGTATCGCCGCTCCCGATTCGCAGCGCATCGCCTTCTATCGCCTTCTTGACGAGTTCTTCTGAGCGGGACTCTGGGGTTCGAAATGACCGACCAAGCGACGCCCAACCTGCCATCACGAGATTTCGATTCCACCGCCGCCTTCTATGAAAGGTTGGGCTTCGGAATCGTTTTCCGGGACGCCGGCTGGATGATCCTCCAGCGCGGGGATCTCATGCTGGAGTTCTTCGCCCACCCTAGGGGGAGGCTAACTGAAACACGGAAGGAGACAATACCGGAAGGAACCCGCGCTATGACGGCAATAAAAAGACAGAATAAAACGCACGGTGTTGGGTCGTTTGTTCATAAACGCGGGGTTCGGTCCCAGGGCTGGCACTCTGTCGATACCCCACCGAGACCCCATTGGGGCCAATACGCCCGCGTTTCTTCCTTTTCCCCACCCCACCCCCCAAGTTCGGGTGAAGGCCCAGGGCTCGCAGCCAACGTCGGGGCGGCAGGCCCTGCCATAGCCTCAGGTTACTCATATATACTTTAGATTGATTTAAAACTTCATTTTTAATTTAAAAGGATCTAGGTGAAGATCCTTTTTGATAATCTCATGACCAAAATCCCTTAACGTGAGTTTTCGTTCCACTGAGCGTCAGACCCCGTAGAAAAGATCAAAGGATCTTCTTGAGATCCTTTTTTTCTGCGCGTAATCTGCTGCTTGCAAACAAAAAAACCACCGCTACCAGCGGTGGTTTGTTTGCCGGATCAAGAGCTACCAACTCTTTTTCCGAAGGTAACTGGCTTCAGCAGAGCGCAGATACCAAATACTGTTCTTCTAGTGTAGCCGTAGTTAGGCCACCACTTCAAGAACTCTGTAGCACCGCCTACATACCTCGCTCTGCTAATCCTGTTACCAGTGGCTGCTGCCAGTGGCGATAAGTCGTGTCTTACCGGGTTGGACTCAAGACGATAGTTACCGGATAAGGCGCAGCGGTCGGGCTGAACGGGGGGTTCGTGCACACAGCCCAGCTTGGAGCGAACGACCTACACCGAACTGAGATACCTACAGCGTGAGCTATGAGAAAGCGCCACGCTTCCCGAAGGGAGAAAGGCGGACAGGTATCCGGTAAGCGGCAGGGTCGGAACAGGAGAGCGCACGAGGGAGCTTCCAGGGGGAAACGCCTGGTATCTTTATAGTCCTGTCGGGTTTCGCCACCTCTGACTTGAGCGTCGATTTTTGTGATGCTCGTCAGGGGGGCGGAGCCTATGGAAAAACGCCAGCAACGCGGCCTTTTTACGGTTCCTGGCCTTTTGCTGGCCTTTTGCTCACATGTTCTTTCCTGCGTTATCCCCTGATTCTGTGGATAACCGTATTACCGCC

>CCW pCMV-TLR1.602S-3FLAG

ATGCATTAGTTATTAATAGTAATCAATTACGGGGTCATTAGTTCATAGCCCATATATGGAGTTCCGCGTTACATAACTTACGGTAAATGGCCCGCCTGGCTGACCGCCCAACGACCCCCGCCCATTGACGTCAATAATGACGTATGTTCCCATAGTAACGCCAATAGGGACTTTCCATTGACGTCAATGGGTGGAGTATTTACGGTAAACTGCCCACTTGGCAGTACATCAAGTGTATCATATGCCAAGTACGCCCCCTATTGACGTCAATGACGGTAAATGGCCCGCCTGGCATTATGCCCAGTACATGACCTTATGGGACTTTCCTACTTGGCAGTACATCTACGTATTAGTCATCGCTATTACCATGGTGATGCGGTTTTGGCAGTACATCAATGGGCGTGGATAGCGGTTTGACTCACGGGGATTTCCAAGTCTCCACCCCATTGACGTCAATGGGAGTTTGTTTTGGCACCAAAATCAACGGGACTTTCCAAAATGTCGTAACAACTCCGCCCCATTGACGCAAATGGGCGGTAGGCGTGTACGGTGGGAGGTCTATATAAGCAGAGCTGGTTTAGTGAACCGTCAGATCCGCTAGCATGACATCCATCTTTCATTTCGCCATTATCTTCATGCTGATACTGCAGATCAGAATCCAGCTGAGCGAGGAAAGCGAGTTCCTGGTGGACAGAAGCAAGAACGGCCTGATTCACGTGCCTAAGGACCTGAGCCAAAAGACCACAATCCTGAATATCAGCCAGAACTACATCTCCGAGCTGTGGACAAGCGACATCCTGTCTCTCTCTAAGCTGCGGATCCTGATCATCAGCCACAATAGAATCCAGTACCTGGACATCAGCGTGTTCAAGTTCAATCAAGAGCTCGAATACCTCGACCTGTCTCACAACAAGCTGGTGAAGATTAGCTGTCACCCTACAGTGAACCTGAAGCACCTGGACCTGTCCTTTAACGCCTTTGACGCCCTGCCTATCTGTAAAGAATTCGGCAACATGAGCCAGCTGAAATTCCTAGGCCTGTCAACAACACACCTGGAGAAGTCTTCTGTTCTTCCTATCGCCCATCTGAATATCAGCAAGGTGCTGCTGGTGCTGGGCGAAACCTACGGCGAGAAGGAAGATCCTGAGGGCCTGCAGGACTTCAACACCGAGTCACTGCACATCGTCTTTCCAACAAACAAGGAATTCCACTTCATCCTGGACGTGAGTGTGAAGACAGTGGCCAACCTGGAGCTGTCTAATATTAAGTGCGTGCTGGAAGATAACAAGTGTAGTTACTTTCTGAGCATCCTGGCTAAACTGCAAACCAACCCCAAGCTGTCCAACCTGACCCTGAACAACATCGAGACCACCTGGAACAGCTTCATCCGGATCCTGCAGCTGGTGTGGCACACCACCGTATGGTATTTCAGCATCAGCAACGTGAAGCTCCAGGGCCAGCTGGACTTTAGAGATTTCGACTACTCCGGCACCAGCCTGAAGGCCCTGTCTATCCACCAGGTGGTGAGCGATGTGTTCGGATTCCCCCAATCTTACATCTACGAAATCTTTAGCAATATGAACATCAAGAACTTCACCGTGTCTGGAACAAGAATGGTGCACATGCTGTGTCCTTCTAAGATCAGTCCTTTCCTGCACCTGGATTTTAGCAACAACCTGCTCACAGACACCGTGTTCGAGAACTGCGGCCACCTGACAGAGTTGGAAACCCTGATCCTGCAGATGAACCAGCTGAAAGAGCTGTCAAAGATCGCCGAGATGACCACACAGATGAAGAGCCTGCAGCAGCTGGATATCAGCCAGAATTCCGTGTCCTACGACGAGAAAAAGGGCGATTGCAGCTGGACTAAGTCTCTTCTTAGCCTGAATATGTCTTCCAACATCCTGACCGACACCATCTTTCGGTGCCTGCCCCCTAGAATCAAGGTGCTGGACCTGCACAGCAACAAAATCAAGAGCATACCTAAGCAGGTGGTCAAGCTGGAAGCCCTGCAGGAGCTGAACGTGGCCTTCAACTCTCTGACCGACCTTCCTGGCTGCGGCAGCTTCAGCAGCCTGAGCGTGCTGATCATCGACCACAACAGCGTGAGCCATCCTAGCGCCGATTTCTTCCAAAGCTGCCAGAAGATGCGGAGCATCAAAGCCGGCGACAACCCTTTCCAGTGCACCTGTGAACTGGGAGAATTCGTCAAAAACATCGACCAGGTTTCTAGCGAAGTGCTGGAAGGCTGGCCTGATTCTTACAAGTGCGACTACCCCGAGAGCTATAGAGGCACCCTGCTGAAAGATTTCCACATGAGCGAACTGAGCTGCAACATAACCCTGCTGATCGTGACTATCGTGGCCACCATGCTGGTGCTCGCTGTGACAGTGACTTCCCTCTGTAGCTACCTGGATCTGCCTTGGTACCTGAGGATGGTGTGCCAGTGGACCCAGACCAGAAGACGGGCCAGAAACATCCCTCTGGAGGAGCTGCAAAGAAATCTGCAGTTCCACGCCTTTATCTCTTATAGCGGACACGACAGCTTCTGGGTGAAGAACGAGCTGCTGCCAAACCTGGAAAAAGAGGGAATGCAGATCTGCCTGCACGAGCGGAACTTCGTCCCAGGCAAGTCCATCGTGGAGAACATCATCACCTGCATCGAGAAGTCCTACAAGAGCATCTTCGTGCTGAGCCCCAATTTCGTCCAGAGCGAGTGGTGCCACTACGAGCTGTACTTCGCTCATCACAACCTGTTCCACGAGGGCAGCAATAGCCTGATTCTGATCCTGCTGGAGCCTATCCCACAGTACAGCATCCCCAGCAGTTACCACAAGCTGAAGTCCCTGATGGCTAGACGCACCTACCTGGAATGGCCCAAGGAAAAATCTAAAAGAGGCTTGTTCTGGGCCAATCTGCGCGCCGCTATCAACATCAAGCTGACCGAGCAGGCCAAGAAGGTCGACCTCGAGGATTACAAGGATGACGACGATAAGGACTATAAGGACGATGATGACAAGGACTACAAAGATGATGACGATAAATAGGGCCCGGTACCTTAATTAATTAAGGTACCAGGTAAGTGTACCCAATTCGCCCTATAGTGAGTCGTATTACAATTCACTCGATCGGCTCGCTGATCAGCCTCGACTGTGCCTTCTAGTTGCCAGCCATCTGTTGTTTGCCCCTCCCCCGTGCCTTCCTTGACCCTGGAAGGTGCCACTCCCACTGTCCTTTCCTAATAAAATGAGGAAATTGCATCGCATTGTCTGAGTAGGTGTCATTCTATTCTGGGGGGTGGGGTGGGGCAGGACAGCAAGGGGGAGGATTGGGAAGACAATAGCAGGCATGCTGGGGAACGCGTAAATTGTAAGCGTTAATATTTTGTTAAAATTCGCGTTAAATTTTTGTTAAATCAGCTCATTTTTTAACCAATAGGCCGAAATCGGCAAAATCCCTTATAAATCAAAAGAATAGACCGAGATAGGGTTGAGTGTTGTTCCAGTTTGGAACAAGAGTCCACTATTAAAGAACGTGGACTCCAACGTCAAAGGGCGAAAAACCGTCTATCAGGGCGATGGCCCACTACGTGAACCATCACCCTAATCAAGTTTTTTGGGGTCGAGGTGCCGTAAAGCACTAAATCGGAACCCTAAAGGGAGCCCCCGATTTAGAGCTTGACGGGGAAAGCCGGCGAACGTGGCGAGAAAGGAAGGGAAGAAAGCGAAAGGAGCGGGCGCTAGGGCGCTGGCAAGTGTAGCGGTCACGCTGCGCGTAACCACCACACCCGCCGCGCTTAATGCGCCGCTACAGGGCGCGTCAGGTGGCACTTTTCGGGGAAATGTGCGCGGAACCCCTATTTGTTTATTTTTCTAAATACATTCAAATATGTATCCGCTCATGAGACAATAACCCTGATAAATGCTTCAATAATATTGAAAAAGGAAGAATCCTGAGGCGGAAAGAACCAGCTGTGGAATGTGTGTCAGTTAGGGTGTGGAAAGTCCCCAGGCTCCCCAGCAGGCAGAAGTATGCAAAGCATGCATCTCAATTAGTCAGCAACCAGGTGTGGAAAGTCCCCAGGCTCCCCAGCAGGCAGAAGTATGCAAAGCATGCATCTCAATTAGTCAGCAACCATAGTCCCGCCCCTAACTCCGCCCATCCCGCCCCTAACTCCGCCCAGTTCCGCCCATTCTCCGCCCCATGGCTGACTAATTTTTTTTATTTATGCAGAGGCCGAGGCCGCCTCGGCCTCTGAGCTATTCCAGAAGTAGTGAGGAGGCTTTTTTGGAGGCCTAGGCTTTTGCAAAGATCGATCAAGAGACAGGATGAGGATCGTTTCGCATGATTGAACAAGATGGATTGCACGCAGGTTCTCCGGCCGCTTGGGTGGAGAGGCTATTCGGCTATGACTGGGCACAACAGACAATCGGCTGCTCTGATGCCGCCGTGTTCCGGCTGTCAGCGCAGGGGCGCCCGGTTCTTTTTGTCAAGACCGACCTGTCCGGTGCCCTGAATGAACTGCAAGACGAGGCAGCGCGGCTATCGTGGCTGGCCACGACGGGCGTTCCTTGCGCAGCTGTGCTCGACGTTGTCACTGAAGCGGGAAGGGACTGGCTGCTATTGGGCGAAGTGCCGGGGCAGGATCTCCTGTCATCTCACCTTGCTCCTGCCGAGAAAGTATCCATCATGGCTGATGCAATGCGGCGGCTGCATACGCTTGATCCGGCTACCTGCCCATTCGACCACCAAGCGAAACATCGCATCGAGCGAGCACGTACTCGGATGGAAGCCGGTCTTGTCGATCAGGATGATCTGGACGAAGAACATCAGGGGCTCGCGCCAGCCGAACTGTTCGCCAGGCTCAAGGCGAGCATGCCCGACGGCGAGGATCTCGTCGTGACCCATGGCGATGCCTGCTTGCCGAATATCATGGTGGAAAATGGCCGCTTTTCTGGATTCATCGACTGTGGCCGGCTGGGTGTGGCGGACCGCTATCAGGACATAGCGTTGGCTACCCGTGATATTGCTGAAGAACTTGGCGGCGAATGGGCTGACCGCTTCCTCGTGCTTTACGGTATCGCCGCTCCCGATTCGCAGCGCATCGCCTTCTATCGCCTTCTTGACGAGTTCTTCTGAGCGGGACTCTGGGGTTCGAAATGACCGACCAAGCGACGCCCAACCTGCCATCACGAGATTTCGATTCCACCGCCGCCTTCTATGAAAGGTTGGGCTTCGGAATCGTTTTCCGGGACGCCGGCTGGATGATCCTCCAGCGCGGGGATCTCATGCTGGAGTTCTTCGCCCACCCTAGGGGGAGGCTAACTGAAACACGGAAGGAGACAATACCGGAAGGAACCCGCGCTATGACGGCAATAAAAAGACAGAATAAAACGCACGGTGTTGGGTCGTTTGTTCATAAACGCGGGGTTCGGTCCCAGGGCTGGCACTCTGTCGATACCCCACCGAGACCCCATTGGGGCCAATACGCCCGCGTTTCTTCCTTTTCCCCACCCCACCCCCCAAGTTCGGGTGAAGGCCCAGGGCTCGCAGCCAACGTCGGGGCGGCAGGCCCTGCCATAGCCTCAGGTTACTCATATATACTTTAGATTGATTTAAAACTTCATTTTTAATTTAAAAGGATCTAGGTGAAGATCCTTTTTGATAATCTCATGACCAAAATCCCTTAACGTGAGTTTTCGTTCCACTGAGCGTCAGACCCCGTAGAAAAGATCAAAGGATCTTCTTGAGATCCTTTTTTTCTGCGCGTAATCTGCTGCTTGCAAACAAAAAAACCACCGCTACCAGCGGTGGTTTGTTTGCCGGATCAAGAGCTACCAACTCTTTTTCCGAAGGTAACTGGCTTCAGCAGAGCGCAGATACCAAATACTGTTCTTCTAGTGTAGCCGTAGTTAGGCCACCACTTCAAGAACTCTGTAGCACCGCCTACATACCTCGCTCTGCTAATCCTGTTACCAGTGGCTGCTGCCAGTGGCGATAAGTCGTGTCTTACCGGGTTGGACTCAAGACGATAGTTACCGGATAAGGCGCAGCGGTCGGGCTGAACGGGGGGTTCGTGCACACAGCCCAGCTTGGAGCGAACGACCTACACCGAACTGAGATACCTACAGCGTGAGCTATGAGAAAGCGCCACGCTTCCCGAAGGGAGAAAGGCGGACAGGTATCCGGTAAGCGGCAGGGTCGGAACAGGAGAGCGCACGAGGGAGCTTCCAGGGGGAAACGCCTGGTATCTTTATAGTCCTGTCGGGTTTCGCCACCTCTGACTTGAGCGTCGATTTTTGTGATGCTCGTCAGGGGGGCGGAGCCTATGGAAAAACGCCAGCAACGCGGCCTTTTTACGGTTCCTGGCCTTTTGCTGGCCTTTTGCTCACATGTTCTTTCCTGCGTTATCCCCTGATTCTGTGGATAACCGTATTACCGCC
