# Supplementary material for: Endosome restricted human TLR1 polymorphism highlights a role for MyD88 in Pam3CSK4 detection
Source: PLoS One. 2026 Oct 5;21(10):e0358252. doi: 10.1371/journal.pone.0358252 (PMC13638051; doi:10.1371/journal.pone.0358252)
Supplement: Supplementary file 3 [file pone.0358252.s006.docx]

**RESPONSE TO REVIEWERS**

**Major Comments**

**Reviewer 1:**

Q: *Lack of negative controls for MyD88 dependency (Figure 5): The authors claim that NF-κB activation by the endosomal TLR1 602S variant is strictly MyD88-dependent based on the siRNA knockdown data in Figure 5B. However, given the generally lower signaling amplitude of the 602S variant compared to 602I (Fig 1D), the ablation of the SEAP signal under MyD88 siRNA conditions might be an artifact of hitting the assay's lower detection limit rather than a complete absence of signaling.*

We thank the reviewer for their comment and have amended our manuscript to reflect the limitations of the study, tempering our statement regarding the strict dependence of TLR1 602S on MyD88.

*Q: To definitively rule out the contribution of endosomal-sorting adaptors traditionally linked to intracellular TLR signaling (such as TRAM/TRIF), the authors should include a parallel TRAM or TRIF siRNA knockdown as a negative control. Showing that TRAM/TRIF depletion does not impact 602S-mediated NF-κB activation is required to support their conclusion.*

We agree with the reviewer. Our initial goal was to evaluate TRAM/TRIF by siRNA knockdown in addition to MyD88. TRAM and TRIF are not negative controls per se, as we and others have published that Pam_3_CSK_4_ activates IL-6 and type I interferons (IFN) from macrophage endosomal compartments via TRAM/TRIF^1-4^. Sadly, the HEK cell system proved to be a limitation in this regard, as expression of both TRAM and TRIF was barely detectable or undetectable (Response Figure 1). Any minor expression observed for TRIF in HEK cells was too low (Ct values 29-32) to reliably measure amount of siRNA knockdown. TRAM expression was entirely undetectable and thus siRNA knockdowns were not possible. In addition, we were unable to measure type I IFN induction in response to Pam_3_CSK_4_, leading us to conclude that the TRAM/TRIF signaling platform was unable to transduce necessary signals in HEK cells. Therefore, we could not examine the role of TRAM/TRIF in TLR1 602S endosomal signaling in this system. We have added a brief clarification of this limitation to the discussion, and have included Response Figure 1 in the supplementary data for the manuscript as S2 Fig.

Response Figure 1: Relative expression of MyD88, TRAM, and TRIF in HEK Blue reporter cells after transfection. Vector transfected MyD88 expression was used as a reference. Beta actin was the housekeeping gene

*Q: Validation of the size-exclusion bead system (Figure 2): The use of 5 μm vs. 0.5 μm streptavidin beads to restrict ligand access to either the cell surface or the endosome is central to the paper’s premise. While the authors refer to confocal imaging in Supplemental Figure 2 (S2), these data should be integrated into the main text or expanded to unequivocally demonstrate that 5 μm beads are completely excluded from the intracellular space in this specific HEK293 reporter line.*

We thank the reviewer for the suggestion. We have included a new panel in Figure 2 composed of the images in the original Supplemental Figure 2 to illustrate the lack of 5µm bead internalization into HEK cells.

*Q: Furthermore, did the authors verify that the coating density and binding affinity of Pam3CSK4-biotin were identical between the 0.5 μm and 5 μm bead formats? This control is necessary to rule out differences in ligand dosage as a confounding factor.*

We thank the reviewer for pointing out this important consideration. We have not measured the amount of Pam_3_CSK_4_ on the beads, however, our primary comparisons are not between bead sizes, but rather between the allelic variants themselves. To more accurately reflect the comparisons we are making, we have altered the layouts in Figure 2C and 2E.

**Minor Comments**

**Reviewer 1:**

*Q: Methodological Caveat: The authors correctly acknowledge that the HEK293 system used in this study lacks the transcriptional machinery necessary for type I IFN induction. Given this substantial limitation, the discussion section should explicitly state that while MyD88 is shown here to mediate endosomal NF-κB signals, the broader physiological interplay between MyD88 and TRAM/TRIF pathways cannot be fully resolved within this specific in vitro model.*

We agree with the reviewer and have included this in our Discussion.

*Q: Typographical Error: The “κ” of NF-κB throughout the text is inconsistent. Please modify it to the correct expression*

We have changed the text of NF-κB to ensure that the “kappa” character is used.

**Reviewer 2:**

*Q: Line 41, TLRs sense widely PAMPs and DAMPs from invertebrates to mammals. A recent review can be mentioned [Toll-like receptor signaling in teleosts. Science China Life Sciences, 2025; 68(7):1889-1911.].*

We have included a citation for the published work at the suggested location.

*Q: Line 251, an example of TLR1 SNP can be supplemented. A TLR1 polymorphism in promoter region in grass carp significantly affect its expression and antibacterial function [Site-specific methylation of a TLR1 promoter SNP impedes CREB binding to impair antibacterial immunity in grass carp. The Journal of Immunology 2026; 215(5):vkag065.].*

We have included a citation for the published work at the suggested location.

*Q: In Table 1, could you supplement beta-actin primer?*

We have added the beta-actin primer oligo sequences.

*Q: In Figure 3A, image block the words, please adjust it.*

We have adjusted the image spacing.

*Q: In Figure 5, “MYD88” should be “MyD88”*

We have corrected the graph’s title.

**References**

1.	Petnicki-Ocwieja T, Chung E, Acosta DI, et al. TRIF mediates Toll-like receptor 2-dependent inflammatory responses to Borrelia burgdorferi. *Infect Immun.* 2013;81(2):402-410.

2.	Petnicki-Ocwieja T, Kern A, Killpack TL, Bunnell SC, Hu LT. Adaptor Protein-3-Mediated Trafficking of TLR2 Ligands Controls Specificity of Inflammatory Responses but Not Adaptor Complex Assembly. *J Immunol.* 2015;195(9):4331-4340.

3.	Nilsen NJ, Vladimer GI, Stenvik J, et al. A Role for the Adaptor Proteins TRAM and TRIF in Toll-like Receptor 2 Signaling. *J Biol Chem.* 2015;290(6):3209-3222.

4.	Stack J, Doyle SL, Connolly DJ, et al. TRAM is required for TLR2 endosomal signaling to type I IFN induction. *J Immunol.* 2014;193(12):6090-6102.
