# Supplementary material for: Endosome restricted human TLR1 polymorphism highlights a role for MyD88 in Pam3CSK4 detection
Source: PLoS One. 2026 Oct 5;21(10):e0358252. doi: 10.1371/journal.pone.0358252 (PMC13638051; doi:10.1371/journal.pone.0358252)
Supplement: Peer review history

# Peer review: Decision Letter 0

**DOI:** 10.1371/journal.pone.0358252.r001

**Authors:** Maria del Mar Ortega-Villaizan

16 Jun 2026

Dear Dr. Petnicki Ocwieja,

Following the reviewers’ comments and the indicated corrections, the manuscript is considered acceptable for publication after **Major Revisions** . Please**address all the issues raised**  and submit the revised manuscript for further evaluation.

Please submit your revised manuscript by Jul 31 2026 11:59PM. If you will need more time than this to complete your revisions, please reply to this message or contact the journal office at plosone@plos.org. When you're ready to submit your revision, log on to https://www.editorialmanager.com/pone/ and select the 'Submissions Needing Revision' folder to locate your manuscript file.

- A letter that responds to each point raised by the academic editor and reviewer(s). You should upload this letter as a separate file labeled 'Response to Reviewers'.
- A marked-up copy of your manuscript that highlights changes made to the original version. You should upload this as a separate file labeled 'Revised Manuscript with Track Changes'.
- An unmarked version of your revised paper without tracked changes. You should upload this as a separate file labeled 'Manuscript'.

If you would like to make changes to your financial disclosure, please include your updated statement in your cover letter. Guidelines for resubmitting your figure files are available below the reviewer comments at the end of this letter.

If applicable, we recommend that you deposit your laboratory protocols in protocols.io to enhance the reproducibility of your results. Protocols.io assigns your protocol its own identifier (DOI) so that it can be cited independently in the future. For instructions see: https://journals.plos.org/plosone/s/submission-guidelines#loc-laboratory-protocols. Additionally, PLOS ONE offers an option for publishing peer-reviewed Lab Protocol articles, which describe protocols hosted on protocols.io. Read more information on sharing protocols at https://plos.org/protocols?utm_medium=editorial-email&utm_source=authorletters&utm_campaign=protocols.

As the corresponding author, your ORCID iD is verified in the submission system and will appear in the published article. PLOS supports the use of ORCID, and we encourage all coauthors to register for an ORCID iD and use it as well. Please encourage your coauthors to verify their ORCID iD within the submission system before final acceptance, as unverified ORCID iDs will not appear in the published article. *Only*  the individual author can complete the verification step; PLOS staff *cannot*  verify ORCID iDs on behalf of authors.

We look forward to receiving your revised manuscript.

Kind regards,

Maria del Mar Ortega-Villaizan

Academic Editor

PLOS One

Journal Requirements:

When submitting your revision, we need you to address these additional requirements.

1.Please ensure that your manuscript meets PLOS ONE's style requirements, including those for file naming. The PLOS ONE style templates can be found at

https://journals.plos.org/plosone/s/file?id=wjVg/PLOSOne_formatting_sample_main_body.pdf and

https://journals.plos.org/plosone/s/file?id=ba62/PLOSOne_formatting_sample_title_authors_affiliations.pdf

2. We note that the grant information you provided in the ‘Funding Information’ and ‘Financial Disclosure’ sections do not match.

When you resubmit, please ensure that you provide the correct grant numbers for the awards you received for your study in the ‘Funding Information’ section.

3. Thank you for stating the following financial disclosure:

“R01AI150157”

Please state what role the funders took in the study.  If the funders had no role, please state: "The funders had no role in study design, data collection and analysis, decision to publish, or preparation of the manuscript."

If this statement is not correct you must amend it as needed.

Please include this amended Role of Funder statement in your cover letter; we will change the online submission form on your behalf.

4. Please provide a complete Data Availability Statement in the submission form, ensuring you include all necessary access information or a reason for why you are unable to make your data freely accessible. If your research concerns only data provided within your submission, please write "All data are in the manuscript and/or supporting information files" as your Data Availability Statement.

5. PLOS ONE now requires that authors provide the original uncropped and unadjusted images underlying all blot or gel results reported in a submission’s figures or Supporting Information files. This policy and the journal’s other requirements for blot/gel reporting and figure preparation are described in detail at https://journals.plos.org/plosone/s/figures#loc-blot-and-gel-reporting-requirements and https://journals.plos.org/plosone/s/figures#loc-preparing-figures-from-image-files. When you submit your revised manuscript, please ensure that your figures adhere fully to these guidelines and provide the original underlying images for all blot or gel data reported in your submission. See the following link for instructions on providing the original image data: https://journals.plos.org/plosone/s/figures#loc-original-images-for-blots-and-gels.

In your cover letter, please note whether your blot/gel image data are in Supporting Information or posted at a public data repository, provide the repository URL if relevant, and provide specific details as to which raw blot/gel images, if any, are not available. Email us at plosone@plos.org if you have any questions.

6. If the reviewer comments include a recommendation to cite specific previously published works, please review and evaluate these publications to determine whether they are relevant and should be cited. There is no requirement to cite these works unless the editor has indicated otherwise.

Additional Editor Comments:

Following the reviewers comments and the indicated corrections, the manuscript is considered acceptable for publication after Major Revisions. Please address all the issues raised and submit the revised manuscript for further evaluation.

[Note: HTML markup is below. Please do not edit.]

Reviewer's Responses to Questions

**Comments to the Author**

1. Is the manuscript technically sound, and do the data support the conclusions?

Reviewer #1: Yes

Reviewer #2: Yes

**********

2. Has the statistical analysis been performed appropriately and rigorously?

Reviewer #1: Yes

Reviewer #2: Yes

**********

3. Have the authors made all data underlying the findings in their manuscript fully available?

The PLOS Data policy

Reviewer #1: Yes

Reviewer #2: Yes

**********

4. Is the manuscript presented in an intelligible fashion and written in standard English?

Reviewer #1: Yes

Reviewer #2: Yes

**********

Reviewer #1: General Comments: This manuscript investigate how the common human TLR1 polymorphism (rs5743618/I602S) affects receptor localization and downstream signaling kinetics, with a specific focus on the role of MyD88 in endosomal TLR2/1 signaling. By combining a modified HEK293 reporter system with size-restricted ligand-coated beads, the authors provide data showing that the endosomally restricted 602S variant activates NF-κB via a MyD88-dependent mechanism. While the spatial isolation setup is interesting, several mechanistic interpretations and experimental controls must be strengthened before publication can be considered.

Major Concerns:

1. Lack of negative controls for MyD88 dependency (Figure 5): The authors claim that NF-κB activation by the endosomal TLR1 602S variant is strictly MyD88-dependent based on the siRNA knockdown data in Figure 5B. However, given the generally lower signaling amplitude of the 602S variant compared to 602I (Fig 1D), the ablation of the SEAP signal under MyD88 siRNA conditions might be an artifact of hitting the assay's lower detection limit rather than a complete absence of signaling. To definitively rule out the contribution of endosomal-sorting adaptors traditionally linked to intracellular TLR signaling (such as TRAM/TRIF), the authors should include a parallel TRAM or TRIF siRNA knockdown as a negative control. Showing that TRAM/TRIF depletion does not impact 602S-mediated NF-κB activation is required to support their conclusion.

2. Validation of the size-exclusion bead system (Figure 2): The use of 5 μm vs. 0.5 μm streptavidin beads to restrict ligand access to either the cell surface or the endosome is central to the paper’s premise. While the authors refer to confocal imaging in Supplemental Figure 2 (S2), these data should be integrated into the main text or expanded to unequivocally demonstrate that 5 μm beads are completely excluded from the intracellular space in this specific HEK293 reporter line. Furthermore, did the authors verify that the coating density and binding affinity of Pam3CSK4-biotin were identical between the 0.5 μm and 5 μm bead formats? This control is necessary to rule out differences in ligand dosage as a confounding factor.

Minor Points:

1. Methodological Caveat: The authors correctly acknowledge that the HEK293 system used in this study lacks the transcriptional machinery necessary for type I IFN induction. Given this substantial limitation, the discussion section should explicitly state that while MyD88 is shown here to mediate endosomal NF-κB signals, the broader physiological interplay between MyD88 and TRAM/TRIF pathways cannot be fully resolved within this specific in vitro model.

2. Typographical Error: The “κ” of NF-κB throughout the text is inconsistent. Please modify it to the correct expression

Reviewer #2: Authors investigated the rs5743618 polymorphism’s effects on TLR1 localization and compartment-specific NF-κB activation. The polymorphism is excluded from the cell surface and retained intracellularly, whereas the ancestral allele localizes to both compartments. Functionally, the ancestral allele signaled from both compartments, while the derived allele signaled exclusively from endosomes. Spatially restricted signaling exhibited influence on IkBα degradation and NF-κB activation compared with the ancestral allele. This activation was dependent on MyD88 localized at ligand-containing endosomes, establishing a role for this adapter in intracellular TLR2/1 signaling. The findings are interesting. The following issues should be clarified.

Minor concerns:

1. Line 41, TLRs sense widely PAMPs and DAMPs from invertebrates to mammals. A recent review can be mentioned [Toll-like receptor signaling in teleosts. Science China Life Sciences, 2025; 68(7):1889-1911.].

2. Line 251, an example of TLR1 SNP can be supplemented. A TLR1 polymorphism in promoter region in grass carp significantly affect its expression and antibacterial function [Site-specific methylation of a TLR1 promoter SNP impedes CREB binding to impair antibacterial immunity in grass carp. The Journal of Immunology 2026; 215(5):vkag065.].

3. In Table 1, could you supplement beta-actin primer?

4. In Figure 3A, image block the words, please adjust it.

5. In Figure 5, “MYD88” should be “MyD88”

**********

what does this mean?). If published, this will include your full peer review and any attached files.

If you choose “no”, your identity will remain anonymous but your review may still be made public.

**Do you want your identity to be public for this peer review?**  For information about this choice, including consent withdrawal, please see our Privacy Policy

Reviewer #1: **Yes:** Jin Zhang

Reviewer #2: No

**********

[NOTE: If reviewer comments were submitted as an attachment file, they will be attached to this email and accessible via the submission site. Please log into your account, locate the manuscript record, and check for the action link "View Attachments". If this link does not appear, there are no attachment files.]

To ensure your figures meet our technical requirements, please review our figure guidelines: https://journals.plos.org/plosone/s/figures

You may also use PLOS’s free figure tool, NAAS, to help you prepare publication quality figures: https://journals.plos.org/plosone/s/figures#loc-tools-for-figure-preparation.

NAAS will assess whether your figures meet our technical requirements by comparing each figure against our figure specifications.

# Author comment: Author response to Decision Letter 1

**DOI:** 10.1371/journal.pone.0358252.r002

23 Jul 2026

RESPONSE TO REVIEWERS

Major Comments

Reviewer 1:

Q: Lack of negative controls for MyD88 dependency (Figure 5): The authors claim that NF-κB activation by the endosomal TLR1 602S variant is strictly MyD88-dependent based on the siRNA knockdown data in Figure 5B. However, given the generally lower signaling amplitude of the 602S variant compared to 602I (Fig 1D), the ablation of the SEAP signal under MyD88 siRNA conditions might be an artifact of hitting the assay's lower detection limit rather than a complete absence of signaling.

We thank the reviewer for their comment and have amended our manuscript to reflect the limitations of the study, tempering our statement regarding the strict dependence of TLR1 602S on MyD88.

Q: To definitively rule out the contribution of endosomal-sorting adaptors traditionally linked to intracellular TLR signaling (such as TRAM/TRIF), the authors should include a parallel TRAM or TRIF siRNA knockdown as a negative control. Showing that TRAM/TRIF depletion does not impact 602S-mediated NF-κB activation is required to support their conclusion.

We agree with the reviewer. Our initial goal was to evaluate TRAM/TRIF by siRNA knockdown in addition to MyD88. TRAM and TRIF are not negative controls per se, as we and others have published that Pam3CSK4 activates IL-6 and type I interferons (IFN) from macrophage endosomal compartments via TRAM/TRIF1-4. Sadly, the HEK cell system proved to be a limitation in this regard, as expression of both TRAM and TRIF was barely detectable or undetectable (Response Figure 1). Any minor expression observed for TRIF in HEK cells was too low (Ct values 29-32) to reliably measure amount of siRNA knockdown. TRAM expression was entirely undetectable and thus siRNA knockdowns were not possible. In addition, we were unable to measure type I IFN induction in response to Pam3CSK4, leading us to conclude that the TRAM/TRIF signaling platform was unable to transduce necessary signals in HEK cells. Therefore, we could not examine the role of TRAM/TRIF in TLR1 602S endosomal signaling in this system. We have added a brief clarification of this limitation to the discussion, and have included Response Figure 1 in the supplementary data for the manuscript as S2 Fig.

Response Figure 1: Relative expression of MyD88, TRAM, and TRIF in HEK Blue reporter cells after transfection. Vector transfected MyD88 expression was used as a reference. Beta actin was the housekeeping gene

Q: Validation of the size-exclusion bead system (Figure 2): The use of 5 μm vs. 0.5 μm streptavidin beads to restrict ligand access to either the cell surface or the endosome is central to the paper’s premise. While the authors refer to confocal imaging in Supplemental Figure 2 (S2), these data should be integrated into the main text or expanded to unequivocally demonstrate that 5 μm beads are completely excluded from the intracellular space in this specific HEK293 reporter line.

We thank the reviewer for the suggestion. We have included a new panel in Figure 2 composed of the images in the original Supplemental Figure 2 to illustrate the lack of 5µm bead internalization into HEK cells.

Q: Furthermore, did the authors verify that the coating density and binding affinity of Pam3CSK4-biotin were identical between the 0.5 μm and 5 μm bead formats? This control is necessary to rule out differences in ligand dosage as a confounding factor.

We thank the reviewer for pointing out this important consideration. We have not measured the amount of Pam3CSK4 on the beads, however, our primary comparisons are not between bead sizes, but rather between the allelic variants themselves. To more accurately reflect the comparisons we are making, we have altered the layouts in Figure 2C and 2E.

Minor Comments

Reviewer 1:

Q: Methodological Caveat: The authors correctly acknowledge that the HEK293 system used in this study lacks the transcriptional machinery necessary for type I IFN induction. Given this substantial limitation, the discussion section should explicitly state that while MyD88 is shown here to mediate endosomal NF-κB signals, the broader physiological interplay between MyD88 and TRAM/TRIF pathways cannot be fully resolved within this specific in vitro model.

We agree with the reviewer and have included this in our Discussion.

Q: Typographical Error: The “κ” of NF-κB throughout the text is inconsistent. Please modify it to the correct expression

We have changed the text of NF-κB to ensure that the “kappa” character is used.

Reviewer 2:

Q: Line 41, TLRs sense widely PAMPs and DAMPs from invertebrates to mammals. A recent review can be mentioned [Toll-like receptor signaling in teleosts. Science China Life Sciences, 2025; 68(7):1889-1911.].

We have included a citation for the published work at the suggested location.

Q: Line 251, an example of TLR1 SNP can be supplemented. A TLR1 polymorphism in promoter region in grass carp significantly affect its expression and antibacterial function [Site-specific methylation of a TLR1 promoter SNP impedes CREB binding to impair antibacterial immunity in grass carp. The Journal of Immunology 2026; 215(5):vkag065.].

We have included a citation for the published work at the suggested location.

Q: In Table 1, could you supplement beta-actin primer?

We have added the beta-actin primer oligo sequences.

Q: In Figure 3A, image block the words, please adjust it.

We have adjusted the image spacing.

Q: In Figure 5, “MYD88” should be “MyD88”

We have corrected the graph’s title.

References

1. Petnicki-Ocwieja T, Chung E, Acosta DI, et al. TRIF mediates Toll-like receptor 2-dependent inflammatory responses to Borrelia burgdorferi. Infect Immun. 2013;81(2):402-410.

2. Petnicki-Ocwieja T, Kern A, Killpack TL, Bunnell SC, Hu LT. Adaptor Protein-3-Mediated Trafficking of TLR2 Ligands Controls Specificity of Inflammatory Responses but Not Adaptor Complex Assembly. J Immunol. 2015;195(9):4331-4340.

3. Nilsen NJ, Vladimer GI, Stenvik J, et al. A Role for the Adaptor Proteins TRAM and TRIF in Toll-like Receptor 2 Signaling. J Biol Chem. 2015;290(6):3209-3222.

4. Stack J, Doyle SL, Connolly DJ, et al. TRAM is required for TLR2 endosomal signaling to type I IFN induction. J Immunol. 2014;193(12):6090-6102.

Attachment Submitted filename: 7-8-26 Response to Reviewers.docx

# Peer review: Decision Letter 1

**DOI:** 10.1371/journal.pone.0358252.r003

**Authors:** Maria del Mar Ortega-Villaizan

31 Aug 2026

Endosome restricted human TLR1 polymorphism highlights a role for MyD88 in Pam3CSK4 detection

PONE-D-26-19809R1

Dear Dr. Petnicki Ocwieja,

We’re pleased to inform you that your manuscript has been judged scientifically suitable for publication and will be formally accepted for publication once it meets all outstanding technical requirements.

Within one week, you’ll receive an e-mail detailing the required amendments. When these have been addressed, you’ll receive a formal acceptance letter and your manuscript will be scheduled for publication.

An invoice will be generated when your article is formally accepted. Please note, if your institution has a publishing partnership with PLOS and your article meets the relevant criteria, all or part of your publication costs will be covered. Please make sure your user information is up-to-date by logging into Editorial Manager at Editorial Manager® and clicking the ‘Update My Information' link at the top of the page. For questions related to billing, please contact billing support.

If your institution or institutions have a press office, please notify them about your upcoming paper to help maximize its impact. If they’ll be preparing press materials, please inform our press team as soon as possible -- no later than 48 hours after receiving the formal acceptance. Your manuscript will remain under strict press embargo until 2 pm Eastern Time on the date of publication. For more information, please contact onepress@plos.org.

Kind regards,

Maria del Mar Ortega-Villaizan

Academic Editor

PLOS One

Additional Editor Comments (optional):

Following reviewers revision, the authors have addressed all the concerns and the article is now acceptable for publication.

Reviewers' comments:

Reviewer's Responses to Questions

**Comments to the Author**

Reviewer #1: (No Response)

Reviewer #2: All comments have been addressed

**********

2. Is the manuscript technically sound, and do the data support the conclusions?

Reviewer #1: (No Response)

Reviewer #2: Yes

**********

3. Has the statistical analysis been performed appropriately and rigorously?

Reviewer #1: (No Response)

Reviewer #2: Yes

**********

4. Have the authors made all data underlying the findings in their manuscript fully available?

The PLOS Data policy

Reviewer #1: (No Response)

Reviewer #2: Yes

**********

5. Is the manuscript presented in an intelligible fashion and written in standard English?

Reviewer #1: (No Response)

Reviewer #2: Yes

**********

Reviewer #1: (No Response)

Reviewer #2: (No Response)

**********

what does this mean?). If published, this will include your full peer review and any attached files.

If you choose “no”, your identity will remain anonymous but your review may still be made public.

**Do you want your identity to be public for this peer review?**  For information about this choice, including consent withdrawal, please see our Privacy Policy

Reviewer #1: No

Reviewer #2: No

**********

# Editor report: Acceptance letter

**DOI:** 10.1371/journal.pone.0358252.r004

**Authors:** Maria del Mar Ortega-Villaizan

PONE-D-26-19809R1

PLOS One

Dear Dr. Petnicki Ocwieja,

I'm pleased to inform you that your manuscript has been deemed suitable for publication in PLOS One. Congratulations! Your manuscript is now being handed over to our production team.

At this stage, our production department will prepare your paper for publication. This includes ensuring the following:

* All references, tables, and figures are properly cited

* All relevant supporting information is included in the manuscript submission,

* There are no issues that prevent the paper from being properly typeset

You will receive further instructions from the production team, including instructions on how to review your proof when it is ready. Please keep in mind that we are working through a large volume of accepted articles, so please give us a few days to review your paper and let you know the next and final steps.

Lastly, if your institution or institutions have a press office, please let them know about your upcoming paper now to help maximize its impact. If they'll be preparing press materials, please inform our press team within the next 48 hours. Your manuscript will remain under strict press embargo until 2 pm Eastern Time on the date of publication. For more information, please contact onepress@plos.org.

You will receive an invoice from PLOS for your publication fee after your manuscript has reached the completed accept phase. If you receive an email requesting payment before acceptance or for any other service, this may be a phishing scheme. Learn how to identify phishing emails and protect your accounts at https://explore.plos.org/phishing.

If we can help with anything else, please email us at customercare@plos.org.

Thank you for submitting your work to PLOS One and supporting open access.

Kind regards,

PLOS One Editorial Office Staff

on behalf of

Dr. Maria del Mar Ortega-Villaizan

Academic Editor

PLOS One
